# 1,8-Bis(dimethylamino)naphthyl-2-ketimines: Inside vs outside protonation

**DOI:** 10.3762/bjoc.14.273

**Published:** 2018-11-28

**Authors:** A S Antonov, A F Pozharskii, P M Tolstoy, A Filarowski, O V Khoroshilova

**Affiliations:** 1 Department of Organic Chemistry, Southern Federal University, Zorge str. 7, 344090 Rostov-on-Don, Russian Federation; 2 Institute of Chemistry, St. Petersburg State University, Universitetskii pr. 26, 198504 St. Petersburg, Russian Federation; 3 Faculty of Chemistry, University of Wroclaw, F. Joliot-Curie 14, 50-383 Wroclaw, Poland; 4 Department of Physics, Industrial University of Tyumen, Volodarskogo 38, 625-000 Tyumen, Russian Federation

**Keywords:** hydrogen bond, imine, NMR, proton sponge, superbase

## Abstract

The structure and protonation behaviour of four *ortho*-arylketimines of 1,8-bis(dimethylamonio)naphthalene with a different number of methoxy groups in an aromatic substituent were investigated in solution by NMR (acetone, DMSO, MeCN), in solid state by X-ray analysis and in the gas phase by DFT calculations. Both mono- and diprotonated species were considered. It has been shown that *E*-isomers of neutral imines can be stabilised by an intramolecular C=N−H···OMe hydrogen bond with a neighbouring methoxy group. Electron-donating OMe groups dramatically increase the basicity of the imino nitrogen, forcing the latter to abstract a proton from the proton sponge moiety in monoprotonated forms. The participation of the *out*-inverted and protonated 1-NMe_2_ group in the Me_2_N−H···NH=C hydrogen bond is experimentally demonstrated. It was shown that the number and position of OMe groups in the aromatic substituents strongly affects the rate of the internal hindered rotation of the NH_2_^+^ fragment in dications.

## Introduction

**Scheme 1:**
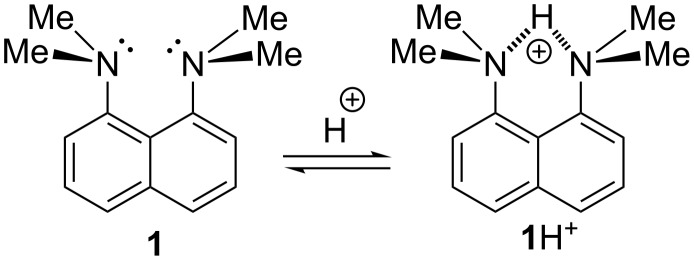
Protonation of DMAN.

**Scheme 2:**
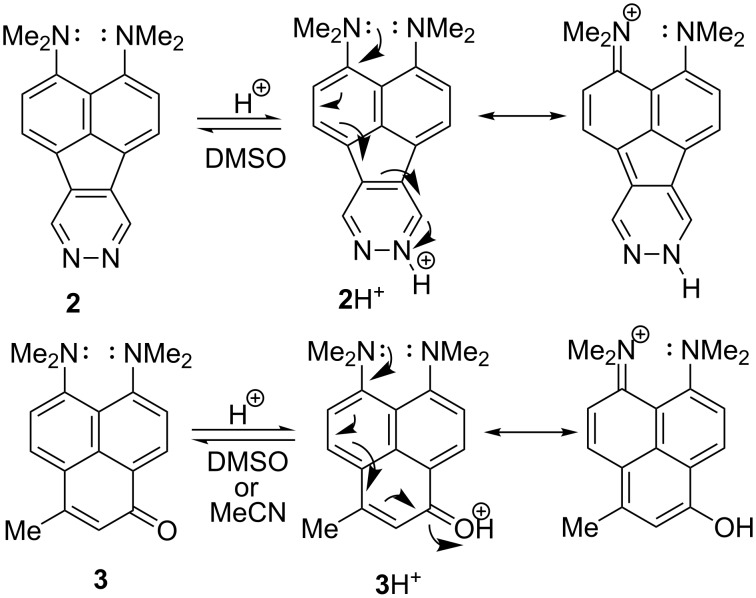
Protonation equilibria for **2** and **3**.

It is well known that the extremely high basicity of 1,8-bis(dimethylamino)naphthalene (**1**, DMAN; p*K*_a_ = 12.1 in H_2_O [1–2], 18.62 in MeCN [3], 7.5 in DMSO [4]) and its derivatives originates from the electrostatic repulsion between the unshared electron pairs of nitrogen atoms, which strongly destabilises the neutral base. This repulsion is additionally fortified by the steric inhibition of resonance (both NMe_2_ groups cannot be conjugated to the aromatic system at the same time) preventing charge delocalisation. The protonation results in the formation of the N–H···N bonded cation **1**H^+^, the removal of the electrostatic and steric strain and thus a considerable free energy gain (Scheme 1) [5]. This is also a reason why the vast majority of DMAN derivatives are protonated to the internitrogen space, even if other centres of basicity are present in the molecule. The only exceptions are compounds **2** and **3** which are protonated to aza- and carbonyl groups, respectively (Scheme 2) [6]. This unusual protonation site originates from the conjugation between the C=N (C=O) and the NMe_2_ groups, leading to the formation of stabilised cations.

**Scheme 3:**
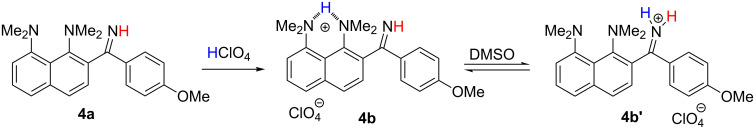
Protonation of imine **4a** with perchloric acid.

**Scheme 4:**
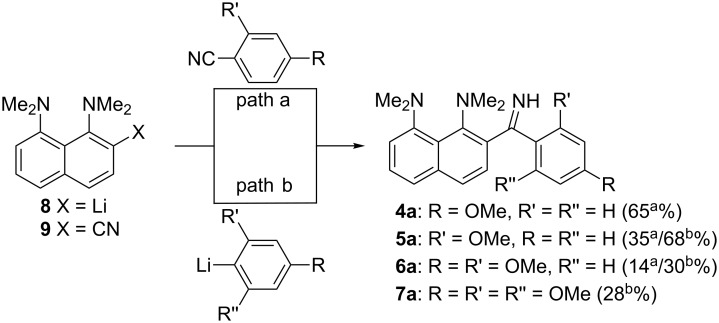
Synthesis of investigated substrates (yields obtained by paths a and b are denoted by a corresponding superscript).

In our recent work, we observed for the first time that the *ortho*-ketimino group in **4a** can compete with the proton sponge moiety for the proton, which results in the formation of equilibria between the forms **4b** (protonated to the proton sponge fragment) and **4b’** (protonated to the imino group) in the DMSO solution (Scheme 3) [7]. The aim of the present work is to investigate the major factors influencing the effective basicity of the imino functionality. As primary objects for this study, we consider a series of imines **4a**–**7a** (Scheme 4), which differ by the number and position of an OMe group in an aryl substituent. We hope that increasing the number of strong electron-donating groups will result in a significant transfer of basicity to the imino function. Selected series of neutral molecules **4a**–**7a**, as well as their mono- (**4b**–**7b**) and diprotonated (**4c**–**7c**) species were investigated by ^1^H NMR spectroscopy in DMSO-*d*_6_, CD_3_CN and acetone-*d*_6_ in a wide range of temperatures. Results obtained in solutions were compared to single crystal X-ray structures and calculated equilibrium geometries of isolated molecules in a vacuum.

## Results and Discussion

### Synthesis of DMAN-*ortho*-ketimines and their cations

**Scheme 5:**
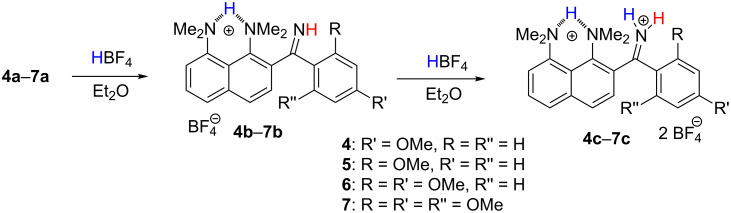
Preparation of protonated forms of imines **4**–**7**.

Target compounds **4a**–**7a** were synthesised by techniques previously developed in our laboratory (Scheme 4) [7–8]. Compounds **4a**–**6a** were obtained by the treatment of the *ortho*-lithium derivative **8** with the corresponding nitrile (path a). Unfortunately, the strong election-donating effect of two and more OMe groups makes this approach not effective for the synthesis of compounds **6a** and **7a**. To overcome this difficulty, we used a reversed method based on the treatment of *ortho*-cyanide **9** with the corresponding aryllithiums, which produces the compounds **5a**–**7a** with good to moderate yields (path b). The monocations **4b**–**7b** and dications **4c**–**7c** were prepared by the treatment of the corresponding imines with one or two equivalents of HBF_4_ in Et_2_O (Scheme 5). The so obtained tetrafluoroborates were used for NMR measurements after recrystallisation from EtOH.

### Structural investigations

**Figure 1:**
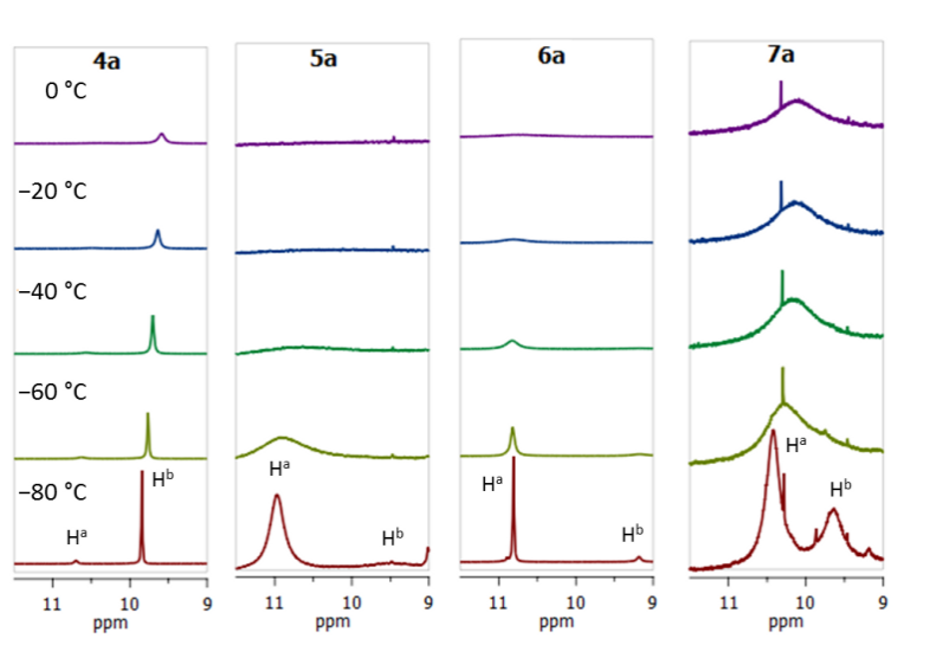
C=NH Signal regions of temperature-depending ^1^H NMR spectra for compounds **4a**–**7a**, acetone-*d*_6_.

**Scheme 6:**
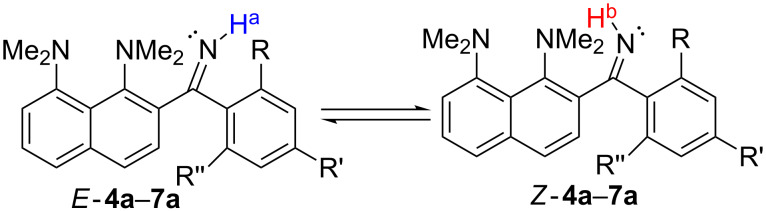
*E/Z*-Isomerisation of ketimines **4a**–**7a**.

**Figure 2:**
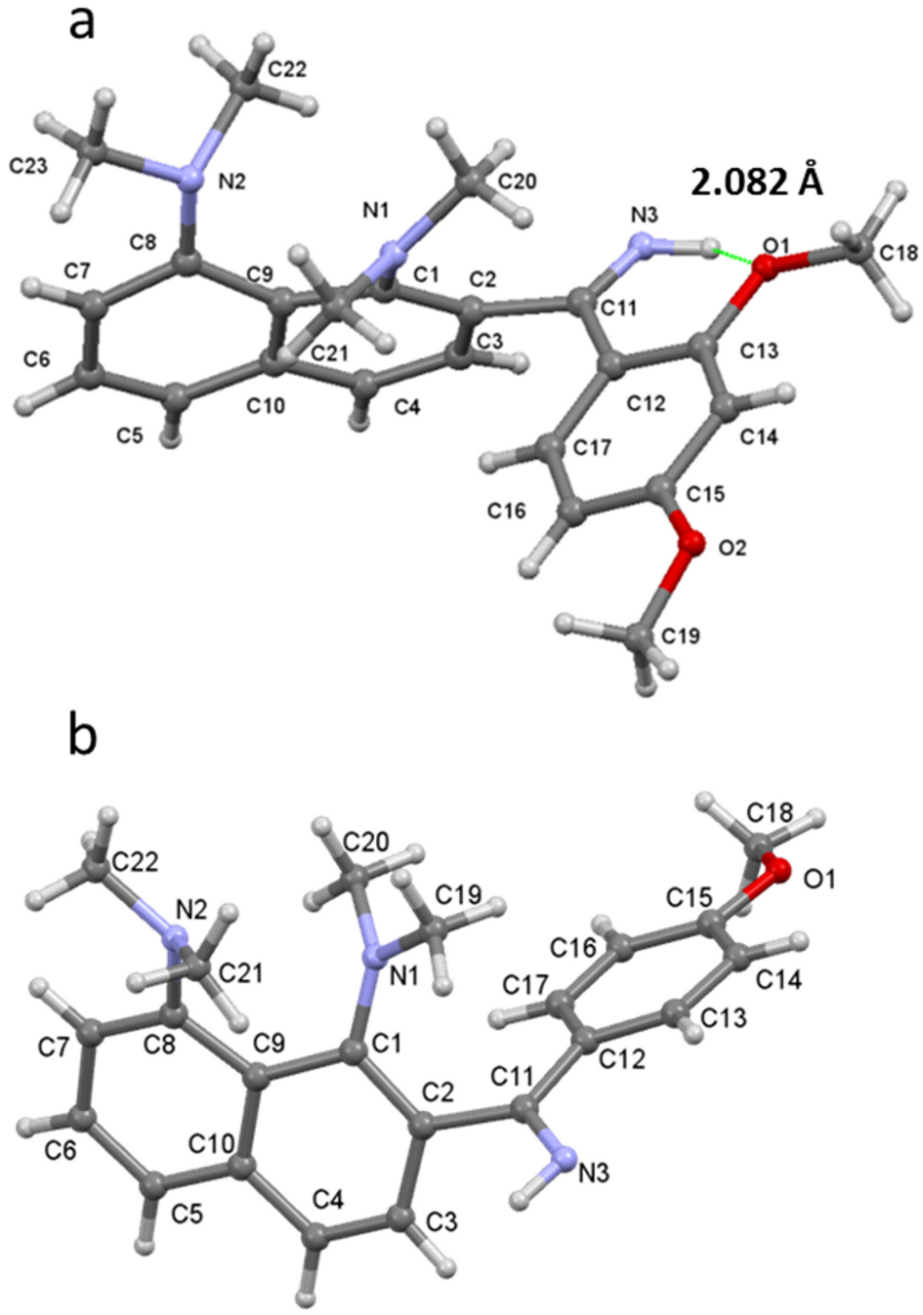
Molecular structure of imines **6a** (a) and **4a** (b). The H···O distance for **6a** is shown.

**Figure 3:**
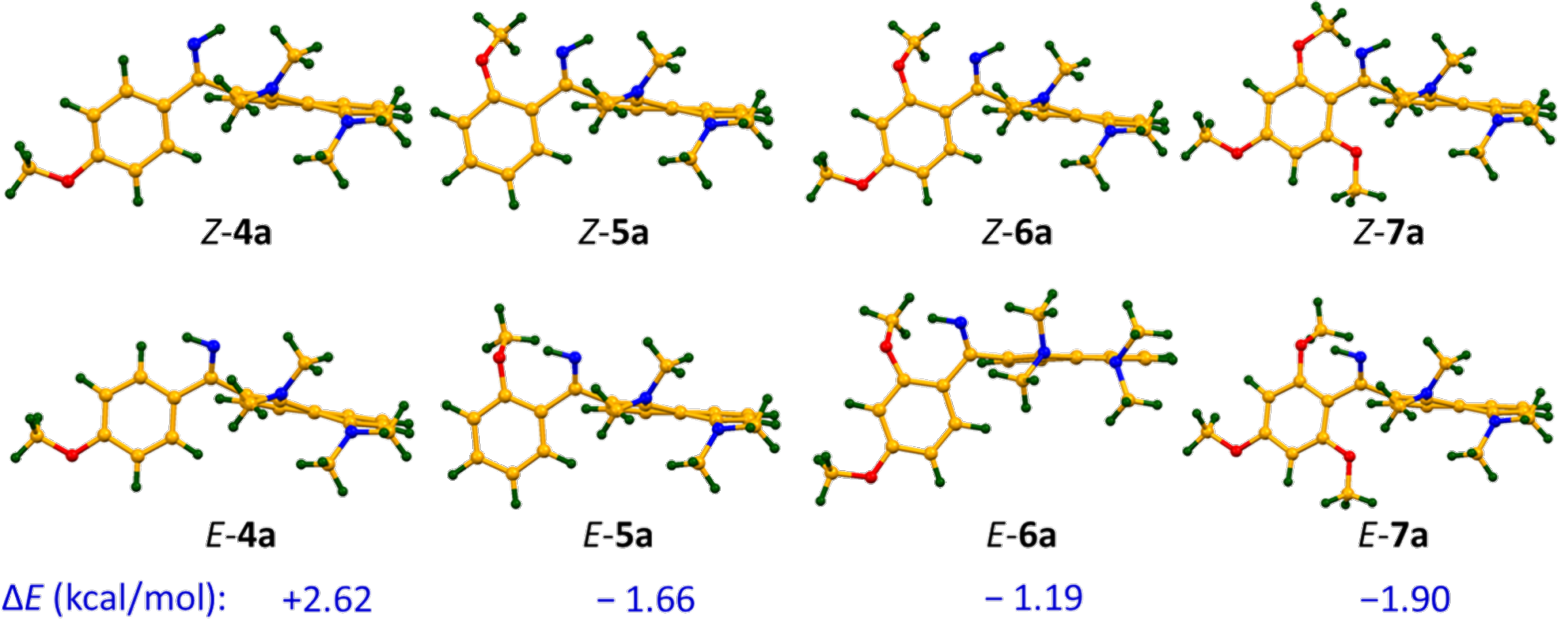
Optimised geometry and energy difference Δ*E* = *E_E_* – *E_Z_* (kcal/mol) for imines **4a**–**7a** (B3LYP/6-311+G(d,p)).

The structural investigations started with a ^1^H NMR analysis of the imines **4a**–**7a** in acetone-*d*_6_, CD_3_CN and DMSO-*d*_6_ in a wide range of temperatures. The main attention was paid to the chemical shift and the shape of the C=NH and NMe_2_ groups’ signals. Upon temperature decrease, the NH signal splits into two peaks of unequal intensity, which is best seen in the case of the acetone-*d*_6_ solution (Figure 1). For example, for compound **4a**, the C=NH proton signals are at δ = 9.9 ppm and 10.7 ppm at −80 °C, with the major signal being in the higher field. In contrast, in the spectra of **5a**–**7a**, bearing OMe groups in *ortho*-position to the imino group, the major NH signal is located in the lower field (Figures S2–S4 in Supporting Information File 1). We assigned these signals to the NH^a^ and NH^b^ protons of *Z*- and *E*-isomers, as shown in Scheme 6. We believe that in solution the *Z*-form is more stable for **4a**, while the *E*-form is more stable for **5a**–**7a**. Indeed, the X-ray analysis shows that compound **6a** in the solid state exists in the *E*-form stabilised by the N−H···O intramolecular hydrogen bond (IHB) with an *ortho*-OMe group (Figure 2a), while compound **4a**, as it was revealed in our previous work [7], exists exclusively as a *Z*-isomer (Figure 2b). Moreover, gas phase quantum chemical calculations show that the *E*-form is preferable for **5a** (Δ*E* = *E_Z_* − *E_E_* = 1.66 kcal/mol), **6a** (Δ*E* = 1.19 kcal/mol), and **7a** (Δ*E* = 1.90 kcal/mol), while the *Z*-form dominates for **4a** (Δ*E* = −2.62 kcal/mol, Figure 3). The contribution of the N−H···O IHB to the stabilisation of the *E*-forms of **5a**–**7a** appears to be small: in the ^1^H NMR spectra, the NH^a^ signal is shifted to the low field by less than 1 ppm in comparison with *Z*-**4a** (Figure 1) [9]; in a DFT calculation, the mutual orientation of C=NH and the OMe groups is not optimal for IHB formation.

The rate and equilibrium constants of this uncatalysed isomerisation [10] depend on the solvent and temperature. By comparing the results obtained in different solvents at equal temperatures, one can see that the H^a^ 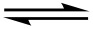 H^b^ exchange rate is noticeably lower in DMSO-*d*_6_ (Figures S9–S12 in Supporting Information File 1) than in acetone-*d*_6_ and CD_3_CN (Figures S5–S8 in Supporting Information File 1), while the chemical shift differences between the H^a^ and H^b^ signals remain similar in all cases (ca. 1 ppm). The increased energy barrier for the isomerisation could be attributed to the stabilisation of the *Z*-form through formation of a stronger hydrogen bond between the DMSO molecule and the NH^b^ proton [11], which is sterically unhindered and accessible for the solvent. Indeed, X-ray data and quantum chemical calculations show that phenyl and naphthalene rings are almost perpendicular (Figure 2 and Figure 3).

**Figure 4:**
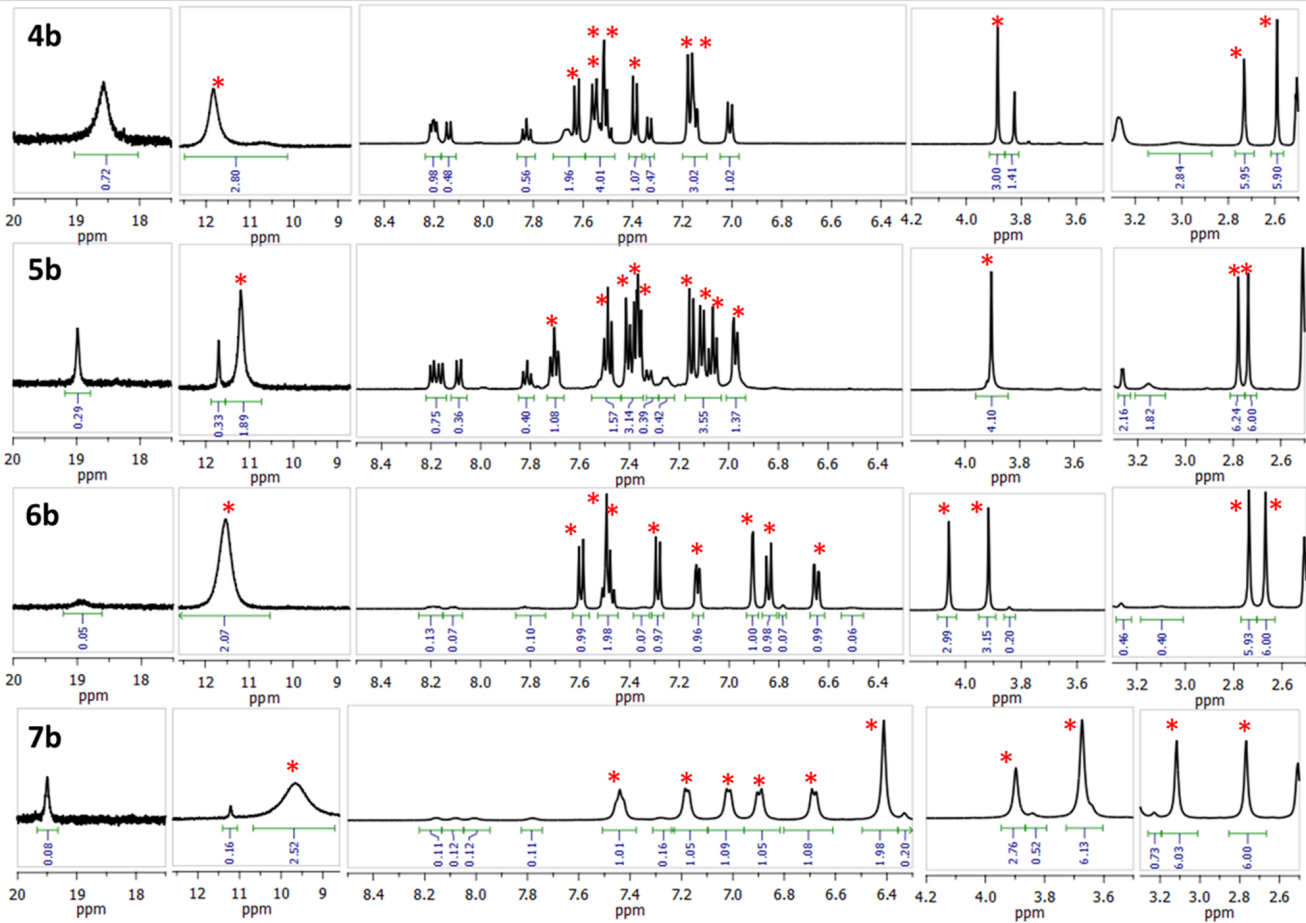
^1^H NMR spectra of imines **4b**–**7b** (**4b’**–**7b’**) in DMSO-*d*_6_, 25 °C (the signals of the forms protonated at the imino function are marked with an asterisk).

**Scheme 7:**
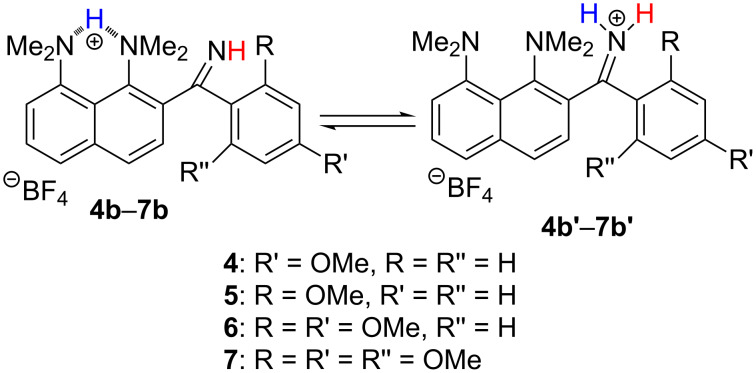
Switching of protonation sites in imines **4b**–**7b**.

After the first protonation of imines **4a**–**7a** with HBF_4_, the ^1^H NMR spectra display two sets of signals (Figure 4). One of the sets corresponds to the monocations **4b**–**7b** protonated at the proton sponge site: the NHN bridging proton resonates at ≈19 ppm and the positions of the CH signals of the dimethylaminonaphthalene fragment are typical and characteristic for protonated proton sponges [5]. In the other set of signals, the chemical shifts of the NMe_2_ groups are practically the same as for the neutral bases **4a**–**7a**. This means that protonation occurs at another basic site, which can only be imine nitrogen, i.e., the structure corresponds to monocations **4b’**–**7b’** (Scheme 7). Indeed, around 10 ppm, a new signal appears which corresponds to two NH protons. To our knowledge, this is the first example of an imine to be basic enough to successfully compete for a proton with the proton sponge moiety: for example, the basicity of benzophenone imine (p*K*_a_ = 6.82) and even that of cyclohexanone imine (p*K*_a_ = 9.15) [12] is much lower than that of DMAN (p*K*_a_ = 12.1) [1–2]. Moreover, as it was already mentioned in the introduction, this is the third known example of a DMAN derivative with a substituent that is more basic than the *peri*-dimethylaminonaphthalene core. Such an imine basicity boost can originate from the fact that upon protonation, NMe_2_ groups lose conjugation with the ring whereas the C=N moiety does not. As a result, the imino nitrogen saves the electron density supply from both OMe and NMe_2_ groups, which increases the s-character of nitrogen hybridisation thus fortifying the basicity [13].

**Figure 5:**
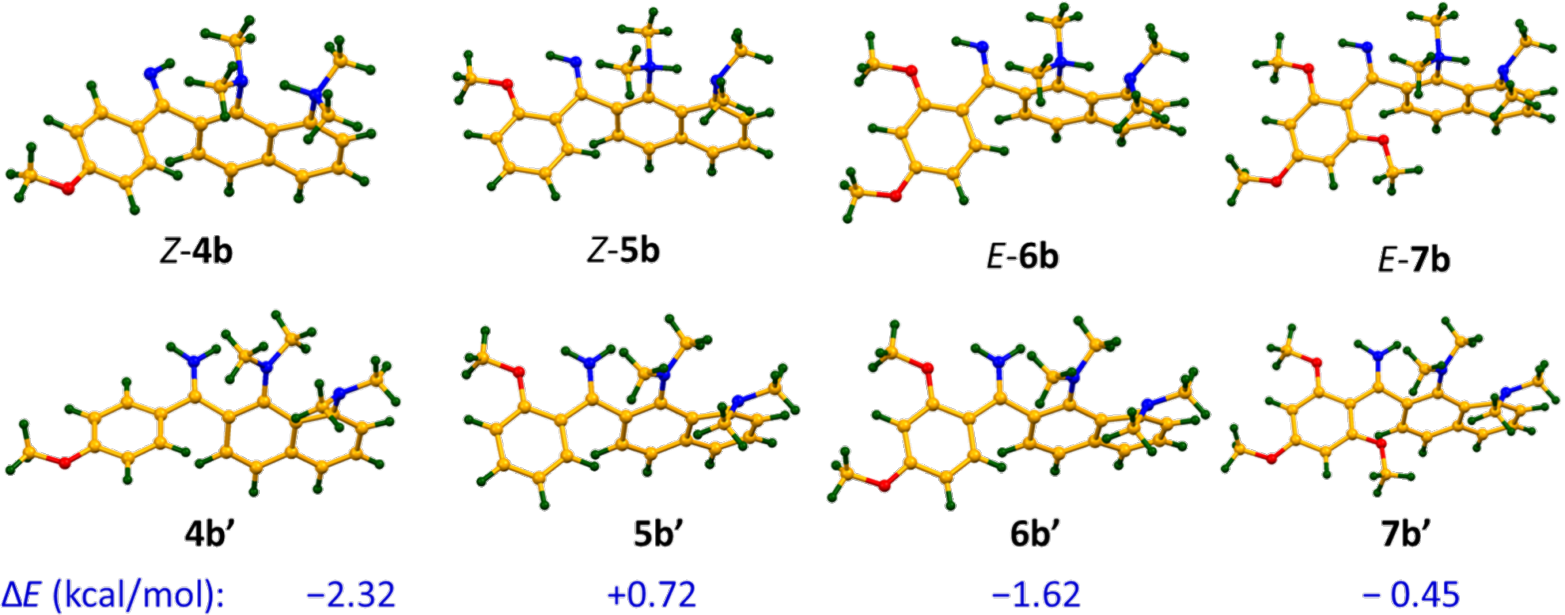
Optimised geometries and energy differences Δ*E* = *E*_b’_ – *E*_b_ (kcal/mol) for different sites of protonation of the studied imines (B3LYP/6-311+G(d,p)).

**Figure 6:**
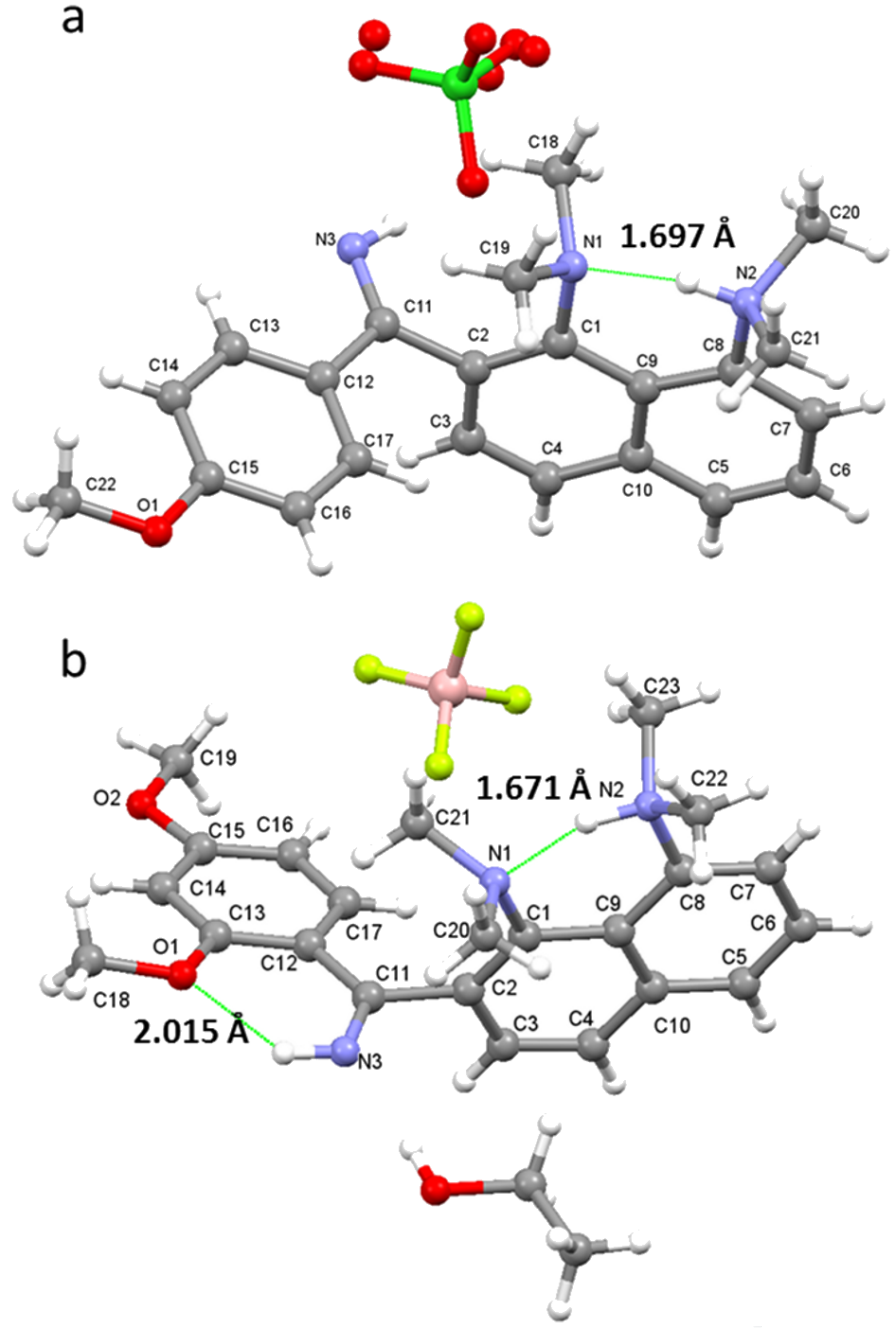
Molecular structure of protonated imines **4a**·HClO_4_ (a) and **6b**·EtOH (b).

The ratio of two monocations, estimated from ^1^H NMR spectra at room temperature, depends on the position and number of the OMe groups and the solvent (Table 1; the values are practically temperature independent). One could expect that strong electron-donating OMe groups being placed in positions 2, 4 or 6 of a phenylimino substituent should increase the basicity of imine nitrogen. Thus, in CD_3_CN, both **4b** and **5b**, containing only one OMe group, show no proton transfer towards the imino function (Figure S13 in Supporting Information File 1). The addition of a second OMe group in the case of **6** leads to the formation of 25% **6b’**. Upon the insertion of a third OMe group in **7**, the amount of **7b’** expectedly increases to 33%. Switching of the solvent to acetone-*d*_6_ facilitates the proton transfer away from the proton sponge moiety (Figure S14, Supporting Information File 1). For example, the relative amount of **7b’** increases from 33% to 66%. Using DMSO-*d*_6_ leads to the dramatic shift of the equilibrium, resulting in the formation of 90% **7b’** (Figure 4). We believe that the forms protonated at the imino function are additionally stabilised by hydrogen bonds with solvent molecules and thus the amount of **b’** forms correlates with the proton accepting ability of the medium [11]. The gas phase calculations show that without any additional interaction with the medium, the forms protonated at the imino group are lowest in energy for the imines **4**, **6**, **7** (Figure 5). In the solid state, no proton transfer to the imino group was observed: compounds **4a**·HClO_4_ and **6b**·EtOH crystallise in forms protonated at the proton sponge moiety (Figure 6).

**Table 1:** Influence of the solvent on the proton transfer to imino nitrogen in **4b**–**7b** solutions at 25 °C.

solvent	amount of protonated form, %							
	
	**4b**	**4b’**	**5b**	**5b’**	**6b**	**6b’**	**7b**	**7b’**

CD_3_CN	100	0	>99	<1	75	25	70	30
acetone-*d*_6_	>99	<1	75	25	30	70	30	70
DMSO-*d*_6_	30	70	30	70	10	90	10	90

**Scheme 8:**
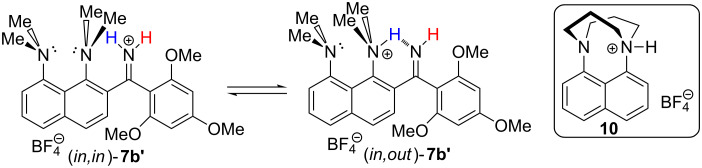
Protonation of the *out*-inverted dialkylamino group.

**Scheme 9:**
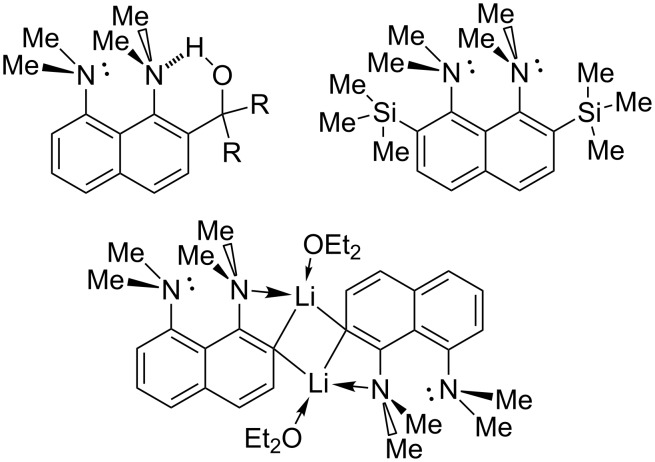
Examples of proton sponges with stabilised *in,out*-conformation.

We expected that the proton transfer to the C=N group will force the nearest proton sponge moiety to occupy the *in,out*-conformation [14] with the 1-NMe_2_ group participating in NHN intramolecular hydrogen bonding with the C=NH_2_^+^ group (Scheme 8). Indeed, in the case of **7b’**, which contains three OMe groups, we have found direct evidence of this process: the 1-NMe_2_ group signal shifts from 2.8 to 3.3 ppm – the characteristic region for protonated dimethylamino groups in proton sponges [5] – while the 8-NMe_2_ group signals stay at 2.8 ppm. Thus, only the 1-NMe_2_ group is protonated, which is only possible if it is *out*-inverted. Similar chemical shifts were observed for the cation **10** (Scheme 8, right), which is *out*-protonated due to steric reasons [15]. In refs. [15–17] it was shown that hydrogen bonding to an *out*-inverted NMe_2_ group without the proton transfer does not noticeably change the chemical shift of methyl protons. Thus, in the case of **7b’**, the chemical shift at 3.3 ppm proves the proton transfer to the amino nitrogen, as shown in Scheme 8 for (*in,out*)-**7b’**. In other words, in the case of **7b’**, steric pressure of the trimethoxyphenyl substituent facilitates the 1-NMe_2_ group’s *out*-inversion which is stabilised by an IHB, forming N(sp^3^)/N(sp^2^) type of proton sponges, which were recently reported in our paper [18]. It was previously shown that the *in,out*-conformation can be stabilised by placing the following functionalities in position 2(7): tertiary alcoholic groups which are able to form the O–H···N IHB [16–17,19], bulky substituents rendering a steric pressure onto the 1-NMe_2_ group [7,20], or a metal atom coordinating the NMe_2_ group as a ligand causing its inversion [21] (Scheme 9). A possibility of *out*-protonation of 1,8-bis(dimethylamino)naphthalene was discussed in our recent paper [15], while it is experimentally demonstrated here for the first time.

**Figure 7:**
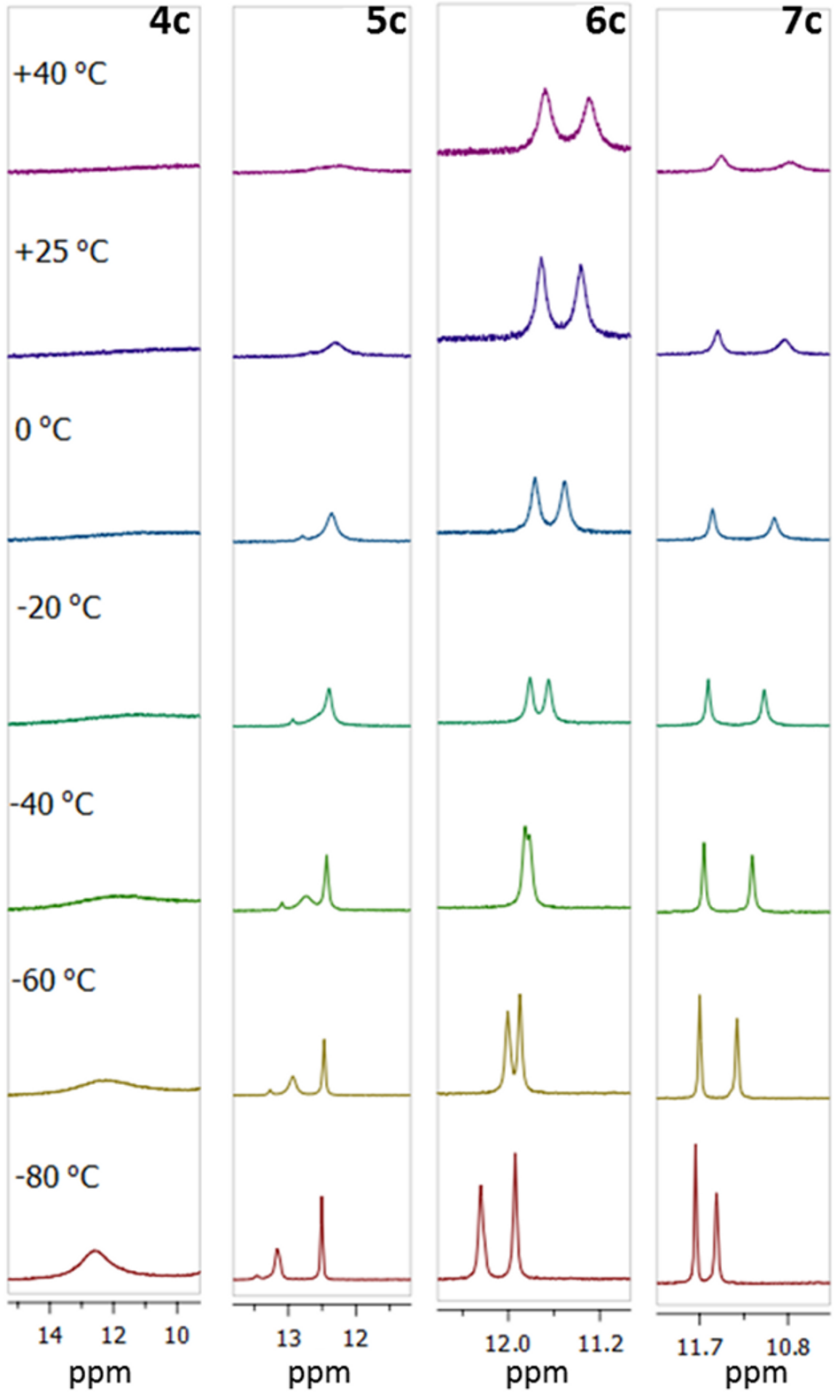
C=NH_2_^+^ Signal regions of temperature-depending spectra for compounds **4c**–**7c**, acetone-*d*_6_.

**Figure 8:**
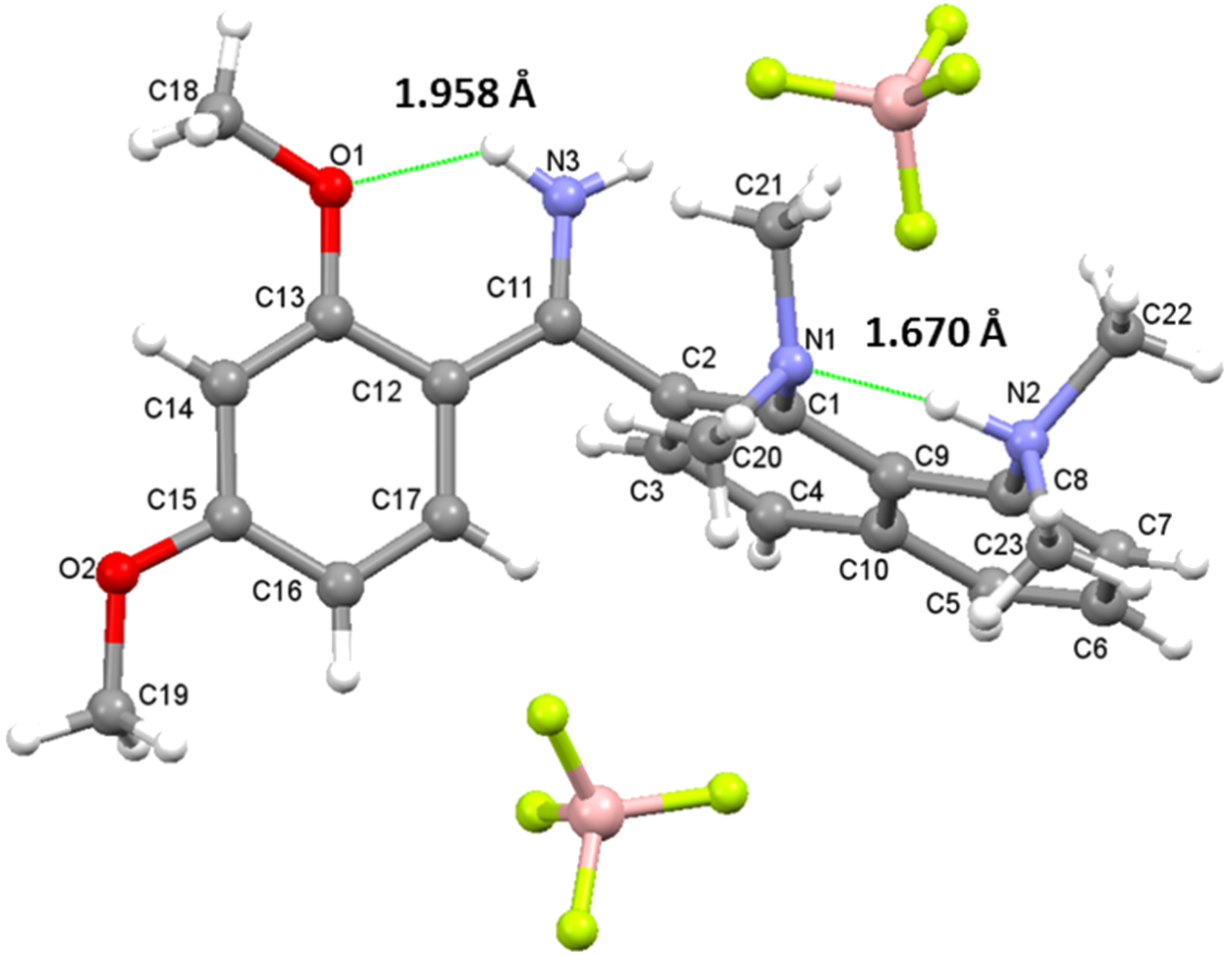
Molecular structure of dication **6с**.

We also prepared dications **4c**–**7c** (Scheme 5) and investigated their structure in solution in CD_3_CN, DMSO-*d*_6_ and acetone-*d*_6_. It was found that the exchange rate between protons in the C=NH_2_^+^ group strongly depends on the position and number of OMe groups and the temperature. In the case of **4c**, signals are coalescent even at −80 °C (Figure 7). The presence of an OMe group in the *ortho-*position to C=N groups in **5c** slows down the exchange and results in NH_2_^+^ signals splitting at −40 °C. This can be attributed to the formation of the weak NHO intramolecular hydrogen bond, which becomes stronger upon inserting an additional OMe group. Such a NHO bond is clearly visible in the X-ray structure of **6c** (Figure 8). As a result, in solutions of **6c** and **7c**, NH_2_^+^ proton signals are observed separately up to 40 °C (for spectra in CD_3_CN see also Figures S20–S23 in Supporting Information File 1).

## Conclusion

In summary, we succeeded in preparing a new series of 1,8-bis(dimethylamino)naphthalene-*ortho*-ketimines with aromatic substituents containing strong electron donating OMe groups. Neutral compounds as well as their mono- and diprotonated species were studied. The obtained compounds demonstrated increased basicity of the imino nitrogen, which was able to effectively compete for the proton with the proton sponge core. Increasing the number of OMe groups facilitated the proton transfer to the imino function by up to 90%. As a result, studied compounds can be considered as superbasic imines. In one case, we were able to experimentally observe the simultaneous *out*-inversion and protonation of the 1-NMe_2_ group for the first time.

## Supporting Information

File 1 Experimental procedures and analytical data, copies of ^1^H NMR spectra of all studied compounds, ^1^H and ^13^C NMR spectra confirming the structure of new compounds, crystallographic data for **6a**, **6b**, **6c** and **4a**·HClO_4_.

File 2 CIF file for compound **6a–c** and **4a**·HClO_4_ (CCDC 1867639-1867642).

## References

[1] Alder R W, Bowman P S, Steele W R S, Winterman D R (1968). Chem Commun.

[2] Hibbert F (1974). J Chem Soc, Perkin Trans 2.

[3] Kaljurand I, Kütt A, Sooväli L, Rodima T, Mäemets V, Leito I, Koppel I A (2005). J Org Chem.

[4] Benoit R L, Lefebvre D, Fréchette M (1987). Can J Chem.

[5] Pozharskii A F, Ozeryanskii V A, Rappoport Z (2007). Proton Sponges. The Chemistry of Anilines.

[6] Pozharskii A F, Ozeryanskii V A, Vistorobskii N V (2003). Russ Chem Bull.

[7] Antonov A S, Mikshiev V Y, Pozharskii A F, Ozeryanskii V A (2014). Synthesis.

[8] Povalyakhina M A, Antonov A S, Dyablo O V, Ozeryanskii V A, Pozharskii A F (2011). J Org Chem.

[9] Afonin A V, Vashchenko A V, Sigalov M V (2016). Org Biomol Chem.

[10] Curtin D Y, Grubbs E J, McCarty C G (1966). J Am Chem Soc.

[11] Laurence C, Graton J, Berthelot M, Besseau F, Le Questel J-Y, Luçon M, Ouvrard C, Planchat A, Renault E (2010). J Org Chem.

[12] Albert A, Serjeant E P (1984). The Ionization Constants of Typical Acids and Bases. The Determination of Ionization Constants.

[13] Alabugin I V, Bresch S, dos Passos Gomes G (2015). J Phys Org Chem.

[14] Szemik-Hojniak A, Zwier J M, Buma W J, Bursi R, van der Waals J H (1998). J Am Chem Soc.

[15] Ozeryanskii V A, Pozharskii A F, Antonov A S, Filarowski A (2014). Org Biomol Chem.

[16] Pozharskii A F, Degtyarev A V, Ryabtsova O V, Ozeryanskii V A, Kletskii M E, Starikova Z A, Sobczyk L, Filarowski A (2007). J Org Chem.

[17] Pozharskii A F, Degtyarev A V, Ozeryanskii V A, Ryabtsova O V, Starikova Z A, Borodkin G S (2010). J Org Chem.

[18] Pozharskii A F, Ozeryanskii V A, Mikshiev V Y, Antonov A S, Chernyshev A V, Metelitsa A V, Borodkin G S, Fedik N S, Dyablo O V (2016). J Org Chem.

[19] Pozharskii A F, Ryabtsova O V, Ozeryanskii V A, Degtyarev A V, Starikova Z A, Sobczyk L, Filarowski A (2005). Tetrahedron Lett.

[20] Pozharskii A F, Ryabtsova O V, Ozeryanskii V A, Degtyarev A V, Kazheva O N, Alexandrov G G, Dyachenko O A (2003). J Org Chem.

[21] Antonov A S, Pozharskii A F, Ozeryanskii V A, Filarowski A, Suponitsky K Y, Tolstoy P M, Vovk M A (2015). Dalton Trans.

