# Supplementary material for: 1,8-Bis(dimethylamino)naphthyl-2-ketimines: Inside vs outside protonation
Source: Beilstein J Org Chem. 2018 Nov 28;14:2940–8. doi: 10.3762/bjoc.14.273 (PMC6278759; doi:10.3762/bjoc.14.273)
Supplement: File 1 — Experimental procedures and analytical data, copies of 1H NMR spectra of all studied compounds, 1H and 13C NMR spectra confirming the structure of new compounds, crystallographic data for 6a, 6b, 6c and 4a·HClO4. [file Beilstein_J_Org_Chem-14-2940-s001.pdf]

BEILSTEIN JOURNAL OF ORGANIC CHEMISTRY

Supporting Information

for

1,8-Bis(dimethylamino)naphthyl-2-ketimines: Inside vs outside protonation

A. S. Antonov, A. F. Pozharskii, P. M. Tolstoy, A. Filarowski and O. V. Khoroshilova

Beilstein J. Org. Chem. **2018,** *14,* 2940–2948. doi:10.3762/bjoc.14.273

Experimental procedures and analytical data, copies of ^{1}H NMR spectra of all studied compounds, ^{1}H and ^{13}C NMR spectra confirming the structure of new compounds, crystallographic data for 6a, 6b, 6c and 4a·$HClO_4$

License and Terms: This is a supporting information file under the terms of the Creative Commons Attribution License (http://creativecommons.org/licenses/by/4.0). Please note that the reuse, redistribution and reproduction in particular requires that the authors and source are credited.
The license is subject to the *Beilstein Journal of Organic Chemistry* terms and conditions: (https://www.beilstein-journals.org/bjoc)

Table of contents

Experimental **S2**

Copies of ^{1}H and ^{13}C NMR spectra **S8**

Crystal data and structure refinement **S29**

Experimental

General

Liquid-state NMR experiments were performed at 500 MHz for ^{1}H and 126 MHz for ^{13}C (Centre for Magnetic Resonance, St. Petersburg State University). Chemical shifts are referred to TMS for ^{1}H and ^{13}C.

The HR-ESI mass-spectra were obtained on a BRUKER maXis spectrometer equipped with an electrospray ionization (ESI) source; methanol was used as the solvent (Chemical Analysis and Materials Research Centre, St. Petersburg State University). The instrument was operated in positive mode using an m/z range of 50–1200. The capillary voltage of the ion source was set at 4000 V. The nebulizer gas pressure was 1.0 bar, and the drying gas flow was set to 4.0 L/min.

Melting points were determined in glass capillaries on a Stuart SMP30 device and are uncorrected.

The quantum mechanical simulations were carried out using the Gaussian 09 suite of program [1]. The energy minimization was performed using DFT with the B3LYP functional [2,3]. The 6-311+G(d,p) basis set was applied during the simulations [4–6]. Harmonic frequencies calculations were performed to confirm that the obtained structures correspond to the minimum on the potential energy surface (PES) and for obtaining *zero-point* Energies (ZPE) values.

For the single crystal X-ray diffraction experiment, crystals were fixed on a micro mount and placed on a SuperNova, Single source at offset/far, HyPix3000 (**5b**, **5c**) and Agilent Technologies Excalibur Eos (**5a**) or Bruker APEX-II CCD (**4a**·$HClO_4$) diffractometers using CuKα and MoKα monochromated radiation, respectively (Centre for X-ray Diffraction Studies, St. Petersburg State University). The crystals were measured at a temperature of 100 K for all samples, except **4a**·$HClO_4$, which was measured at a temperature of 120 K. The structures have been solved by the ShelXS [7] and ShelXT [8] structure solution programs using Direct Methods and Intrinsic Phasing, respectively, and were refined by means of the ShelXL program [8] incorporated in the OLEX2 program package [9]. The carbon and nitrogen-bound H atoms were placed in calculated positions and were included in the refinement in the ‘riding’ model approximation, with $U_{iso}(H)$ set to $1.5U_{eq}(C)$ and C–H 0.96 Å for the CH_3 groups, $U_{iso}(H)$ set to $1.2U_{eq}(C)$ and C–H 0.97 Å for the CH_2 groups, $U_{iso}(H)$ set to $1.2U_{eq}(C)$ and C–H 0.93 Å for the CH groups and $U_{iso}(H)$ set to $1.2U_{eq}(N)$ and N–H 0.86 Å for the NH_2 and NH groups. Supplementary crystallographic data for this paper have been deposited at the Cambridge Crystallographic Data Centre (CCDC 1867639-1867642) and can be obtained free of charge via www.ccdc.cam.ac.uk/data_request/cif.

Preparation of reagents

2-Lithioanisole

To a solution of 2-bromoanisole (1571 mg; 8.4 mmol) in 10 mL of dry Et_2O cooled to −20 °C a 1.6 M a solution of *n*-BuLi in hexanes (5.3 mL; 8.4 mmol) was added; the reaction mixture was kept at −20 °C under an argon atmosphere for 60 min.

2,4-Dimethoxyphenyllithium

To a solution of 2,4-dimethoxybromobensene (1883 mg; 8.4 mmol) in 10 mL of dry Et_2O cooled to −20 °C a 1.6 M a solution of *n*-BuLi in hexanes (5.3 mL; 8.4 mmol) was added; the reaction mixture was kept at −20 °C under an argon atmosphere for 30 min.

2,4,6-Trimethoxyphenyllithium

To a solution of 2,4,6-trimethoxybromobensene (2075 mg; 8.4 mmol) in 10 mL of dry Et_2O cooled to −20 °C a 1.6 M a solution of *n*-BuLi in hexanes (5.3 mL; 8.4 mmol) was added; the reaction mixture was kept at −20 °C under an argon atmosphere for 30 min.

Synthesis of imines 4a–7a starting from 2-lithium-1,8-bis(dimethylamino)naphthalene (path A)

In a similar manner as described before [10], to a solution of 2-lithium-1,8-bis(dimethylamino)naphthalene (**2**) obtained by a standard technique [11] from 1000 mg (3.4 mmol) of 2-bromo-1,8-bis(dimethylamino)naphthalene (**1b**) a solution of the corresponding nitrile in 10 mL of dry Et_2O was added via syringe under an argon atmosphere at −20 °C. The red-coloured reaction mixture was kept at room temperature for 24–96 h and treated with 10 mL of distilled water. The yellow ether solution was separated, and the aqueous phase was extracted three times with chloroform (10 mL). The organic fractions were combined, evaporated and chromatographed on alumina.

Synthesis of imines 4a–7a starting from 2-cyano-1,8-bis(dimethylamino)naphthalene (path B)

To a solution of the corresponding aryllithium (see above) 1000 mg (4.2 mmol) of 2-cyanonaphthalene [12] in 20 mL of absolute Et_2O was added via syringe under an argon atmosphere at –20 °C. The red-coloured reaction mixture was kept at room temperature for 24 h and treated with 10 mL of water. The yellow ether solution was separated and the aqueous phase was extracted with chloroform (30 mL). The organic fractions were combined, evaporated and chromatographed on alumina.

*2-(Imino(4-methoxyphenyl)methyl)-1,8-bis(dimethylamino)naphthalene (**4a**)*

Obtained by path A from 4-methoxybenzonitrile. Yield: 769 mg (65%). Characterization data were consistent with those reported in the literature [12]. HRMS (ESI): 348.2056 [M+H$^+$]; calculated for $C_{22}H_{25}N_2O_3$ [M+H$^+$] 348.2070.

*2-(Imino(2-methoxyphenyl)methyl)-1,8-bis(dimethylamino)naphthalene (**5a**)*

Obtained by path A from 2-methoxybenzonitrile with yield: 414 mg (35%), or by path B from 2-methoxyphenyllithium with yield: 994 mg (68%). Dark orange oil. ^{1}H NMR (500 MHz, CD_3CN) δ 10.29 (brs, 1H), 7.49 – 7.32 (m, 4H), 7.24 (d, *J* = 8.2 Hz, 1H), 7.12 – 7.02 (m, 3H), 6.90 (t, *J* = 7.4 Hz, 1H), 3.73 (s, 3H), 2.75 (s, 6H), 2.65 (s, 6H). ^{13}C NMR (126 MHz, CD_3CN) δ 176.95, 158.14, 152.30, 146.96, 138.30, 134.45, 131.05, 130.48, 128.40, 127.71, 126.11, 123.17, 122.33, 121.94, 120.07, 113.91, 111.97, 55.22, 44.38, 43.52. HRMS (ESI): 348.2069 [M+H$^+$]; calculated for $C_{22}H_{25}N_2O_3$ [M+H$^+$] 348.2070.

*2-(Imino(2,4-dimethoxyphenyl)methyl)-1,8-bis(dimethylamino)naphthalene (**6a**)*

Obtained by path A from 2,4-dimethoxybenzonitrile with yield: 180 mg (14%), or by path B from 2,4-dimethoxyphenyllithium with yield: 476 mg (30%). Yellow crystals, m.p. 125–126 °C (n-hexane) ^{1}H NMR (500 MHz, CD_3CN) δ 10.43 (brs, 1H), 7.48 – 7.39 (m, 2H), 7.34 (t, *J* = 7.7 Hz, 1H), 7.20 (d, *J* = 7.9 Hz, 1H), 7.06 (d, *J* = 7.4 Hz, 1H), 6.63 (d, *J* = 1.2 Hz, 1H), 6.44 (d, *J* = 7.8 Hz, 1H), 3.81 (s, 3H), 3.78 (brs, 3H), 2.76 (s, 6H), 2.68 (s, 6H). ^{13}C NMR (126 MHz, CD_3CN) δ 176.37, 162.22, 159.89, 152.19, 146.80, 138.18, 134.47, 132.22, 127.75, 125.98, 122.93, 122.18, 121.79, 120.87, 113.71, 104.53, 98.79, 55.34, 55.16, 44.26, 43.60. HRMS (ESI): 378.2183 [M+H$^+$]; calculated for $C_{22}H_{25}N_2O_3$ [M+H$^+$] 378.2176. For X-Ray measurements, the crystals were obtained from *n*-hexane. The details of data collection and crystal structure refinement are summarized in Table SI-1 (Supporting Info). CCDC reference number 1867639.

*2-(Imino(2,4,6-trimethoxyphenyl)methyl)-1,8-bis(dimethylamino)naphthalene (**7a**)*

Obtained by path B from 2,4,5-trimethoxyphenyllithium with yield: 480 mg (28%). Dark orange oil. ^{1}H NMR (500 MHz, CD_3CN) δ 10.04 (s, 1H), 7.39 – 7.29 (m, 3H), 7.12 (d, *J* = 8.2 Hz, 1H), 7.07 (d, *J* = 7.3 Hz, 1H), 6.27 (s, 2H), 3.85 (s, 3H), 3.62 (s, 6H), 2.80 (s, 6H), 2.78 (s, 6H). ^{13}C NMR (126 MHz, CD_3CN) δ 172.32, 161.71, 158.98, 152.64, 146.93, 138.37, 127.53, 127.28, 126.70, 125.98, 123.52, 121.83, 121.77, 113.74, 112.49, 55.37, 55.16, 44.54, 43.50. HRMS (ESI): 408.2297 [M+H$^+$]; calculated for $C_{22}H_{25}N_2O_3$ [M+H$^+$] 408.2282.

Synthesis of imines monocations

To a solution of the corresponding imine **4a–7a** in 5 mL of Et_2O one equivalent of tetrafluoroboric acid solution was added. The precipitate was filtered, washed with Et_2O and recrystallised from EtOH to give pure **4b–7b**.

*2-(Imino(4-methoxyphenyl)methyl)-1,8-bis(dimethylamino)naphthalene tetrafluoroborate (**4b/4b'**).* Colorless crystals, 189 °C decomp.

4b: ^{1}H NMR (500 MHz, CD_3CN, 25 °C) δ 19.20 (s, 1H), 10.43 (s, 1H), 8.14 (d, *J* = 8.2 Hz, 1H), 8.09 (d, *J* = 8.5 Hz, 1H), 8.00 (dd, *J* = 7.6, 0.8 Hz, 1H), 7.80 (t, *J* = 7.9 Hz, 1H), 7.67 (d, *J* = 8.5 Hz, 2H), 7.35 (d, *J* = 8.4 Hz, 1H), 7.00 (d, *J* = 9.1 Hz, 2H), 3.86 (s, 3H), 3.22 (d, *J* = 3.2 Hz, 6H), 3.04 (s, 6H).

4b': ^{1}H NMR (500 MHz, DMSO 25 °C) δ 11.83 (s, 2H), 7.63 (d, *J* = 8.5 Hz, 1H), 7.58 – 7.48 (m, 4H), 7.39 (d, *J* = 8.4 Hz, 1H), 7.20 – 7.11 (m, 3H), 3.89 (s, 3H), 2.73 (s, 6H), 2.59 (s, 6H).

*2-(Imino(2-methoxyphenyl)methyl)-1,8-bis(dimethylamino)naphthalene tetrafluoroborate (**5b/5b'**).* Colorless crystals, 148 °C decomp.

5b: ^{1}H NMR (500 MHz, CD_3CN, −40 °C) δ 19.27 (s, 1H), 11.70 (s, 1H), 8.12 (d, *J* = 8.1 Hz, 1H), 8.06 (d, *J* = 8.5 Hz, 1H), 8.02 (d, *J* = 7.4 Hz, 1H), 7.80 (t, *J* = 7.9 Hz, 1H), 7.59 – 7.54 (m, 1H), 7.34 (d, *J* = 8.5 Hz, 1H), 7.26 (d, *J* = 8.4 Hz, 1H), 7.10 (d, *J* = 7.6 Hz, 1H), 6.93 (t, *J* = 7.5 Hz, 1H), 4.01 (s, 3H), 3.19 (d, *J* = 2.9 Hz, 6H), 3.09 (s, 6H).

5b': ^{1}H NMR (500 MHz, DMSO, 25 °C) δ 11.19 (s, 2H), 7.73 – 7.68 (m, 1H), 7.49 (t, *J* = 7.8 Hz, 1H), 7.42 – 7.34 (m, 3H), 7.15 (d, *J* = 8.5 Hz, 1H), 7.11 (d, *J* = 7.4 Hz, 1H), 7.06 (t, *J* = 7.6 Hz, 1H), 6.97 (dd, *J* = 7.7, 1.3 Hz, 1H), 3.90 (s, 3H), 2.78 (s, 6H), 2.74 (s, 6H).

*2-(Imino(2,4-dimethoxyphenyl)methyl)-1,8-bis(dimethylamino)naphthalene tetrafluoroborate (**6b**).* Colorless crystals, 190 °C decomp.

6b: ^{1}H NMR (500 MHz, CD_3CN, −40 °C) δ 18.92 (s, 1H), 11.35 (s, 1H), 8.14 (d, *J* = 8.0 Hz, 1H), 8.08 (d, *J* = 8.5 Hz, 1H), 8.04 (d, *J* = 7.5 Hz, 1H), 7.82 (t, *J* = 7.9 Hz, 1H), 7.35 (d, *J* = 8.5 Hz, 1H), 7.04 (d, *J* = 8.9 Hz, 1H), 6.77 (d, *J* = 2.1 Hz, 1H), 6.49 (dd, *J* = 8.9, 2.2 Hz, 1H), 4.07 (s, 3H), 3.87 (s, 3H), 3.21 (d, *J* = 2.9 Hz, 6H), 3.03 (s, 6H). For X-ray measurements, the crystals were obtained from EtOH. The details of data collection and crystal structure refinement are summarized in Table SI-1. CCDC reference number 1867640.

6b': ^{1}H NMR (500 MHz, DMSO, 25 °C) δ 11.54 (s, 2H), 7.60 (d, *J* = 8.5 Hz, 1H), 7.52 – 7.45 (m, 2H), 7.29 (d, *J* = 8.4 Hz, 1H), 7.13 (dd, *J* = 6.9, 1.5 Hz, 1H), 6.91 (d, *J* = 2.1 Hz, 1H), 6.84 (d, *J* = 9.0 Hz, 1H), 6.65 (dd, *J* = 9.0, 2.2 Hz, 1H), 4.06 (s, 3H), 3.92 (s, 3H), 2.74 (s, 6H), 2.67 (s, 6H).

*2-(Imino(2,4,6-dimethoxyphenyl)methyl)-1,8-bis(dimethylamino)naphthalene tetrafluoroborate (**7b**).* Colorless crystals, 85 °C decomp.

7b: ^{1}H NMR (500 MHz, CD_3CN, 25 °C) δ 19.80 (s, 1H), 10.84 (s, 1H), 8.02 (d, *J* = 8.1 Hz, 1H), 7.95 (d, *J* = 8.7 Hz, 2H), 7.75 (t, *J* = 7.9 Hz, 1H), 7.35 (d, *J* = 8.6 Hz, 1H), 6.29 (s, 2H), 3.87 (s, 3H), 3.66 (s, 6H), 3.39 (d, *J* = 2.4 Hz, 6H), 3.16 (d, *J* = 1.5 Hz, 6H).

7b': ^{1}H NMR (500 MHz, DMSO, 25 °C) δ 9.66 (s, 2H), 7.47 – 7.39 (m, 1H), 7.18 (d, *J* = 6.5 Hz, 1H), 7.02 (d, *J* = 6.8 Hz, 1H), 6.90 (d, *J* = 7.9 Hz, 1H), 6.68 (d, *J* = 8.0 Hz, 1H), 6.41 (s, 2H), 3.90 (s, 3H), 3.67 (s, 6H), 3.12 (s, 6H), 2.77 (s, 6H).

2-(Imino(4-methoxyphenyl)methyl)-1,8-bis(dimethylamino)naphthalene perchlorate (**4a**·$HClO_4$) was obtained as it was described previously [10]. For X-ray measurements, the crystals were obtained from EtOH. The details of data collection and crystal structure refinement are summarized in Table SI-1. CCDC reference number 1867642.

Synthesis of imines dications

To a solution of the corresponding imine **4a–7a** in 5 mL of Et_2O 2 equivalents of tetrafluoroboric acid solution was added. The precipitate was filtered, washed with Et_2O and recrystallised from EtOH to give pure **4c–7c**.

*2-(Imino(4-methoxyphenyl)methyl)-1,8-bis(dimethylamino)naphthalene ditetrafluoroborate (**4c**).* Colorless crystals, 175 °C decomp.

^{1}H NMR (500 MHz, CD_3CN, 25 °C) δ 17.62 (s, 1H), 8.25 (d, *J* = 8.2 Hz, 1H), 8.21 (d, *J* = 8.5 Hz, 1H), 8.11 (d, *J* = 7.6 Hz, 1H), 7.95 – 7.85 (m, 1H), 7.49 (d, *J* = 8.5 Hz, 1H), 7.19 (d, *J* = 9.1 Hz, 1H), 3.98 (s, 1H), 3.33 (d, *J* = 4.0 Hz, 6H), 2.92 (s, 6H).

*2-(Imino(2-methoxyphenyl)methyl)-1,8-bis(dimethylamino)naphthalene ditetrafluoroborate (**5c**).* Colorless crystals, 194 °C decomp.

^{1}H NMR (500 MHz, CD_3CN, 25 °C) δ 17.28 (s, 1H), 11.57 (s, 2H), 8.27 (d, *J* = 8.1 Hz, 1H), 8.23 (d, *J* = 8.6 Hz, 1H), 8.14 (d, *J* = 7.6 Hz, 1H), 8.01 – 7.90 (m, 2H), 7.53 – 7.46 (m, 2H), 7.38 (dd, *J* = 8.1, 1.5 Hz, 1H), 7.13 (t, *J* = 7.7 Hz, 1H), 4.29 (s, 3H), 3.36 (d, *J* = 4.1 Hz, 6H), 2.91 (s, 6H).

*2-(Imino(2,4-dimethoxyphenyl)methyl)-1,8-bis(dimethylamino)naphthalene ditetrafluoroborate (**6c**).* Colorless crystals, 194 °C decomp.

^{1}H NMR (500 MHz, CD_3CN, 25 °C) δ 17.49 (s, 1H), 10.98 (s, 1H), 10.16 (s, 1H), 8.26 (d, *J* = 8.2 Hz, 1H), 8.22 (d, *J* = 8.5 Hz, 1H), 8.13 (d, *J* = 7.6 Hz, 1H), 7.92 (t, *J* = 8.0 Hz, 1H), 7.48 (d, *J* = 8.5 Hz, 1H), 7.27 (d, *J* = 9.2 Hz, 1H), 6.90 (d, *J* = 1.7 Hz, 1H), 6.66 (dd, *J* = 9.2, 1.8 Hz, 1H), 4.28 (s,

3H), 4.03 (s, 3H), 3.39 (s, 3H), 3.30 (s, 3H), 3.09 (s, 3H), 2.78 (s, 3H). For X-ray measurements, the crystals were obtained from EtOH. The details of data collection and crystal structure refinement are summarized in Table SI-1. CCDC reference number 1867641.

2-(Imino(2,4,6-dimethoxyphenyl)methyl)-1,8-bis(dimethylamino)naphthalene tetrafluoroborate (**7c**). Colorless crystals, 148 °C decomp.

^{1}H NMR (500 MHz, CD_3CN, 60 °C) δ 17.78 (s, 1H), 10.75 (s, 1H), 9.59 (s, 1H), 8.21 (d, *J* = 8.2 Hz, 1H), 8.16 (d, *J* = 8.6 Hz, 1H), 8.07 (d, *J* = 7.6 Hz, 1H), 7.87 (t, *J* = 8.0 Hz, 1H), 7.45 (d, *J* = 8.6 Hz, 1H), 6.39 (s, 2H), 4.05 (s, 3H), 3.78 (s, 6H), 3.34 (d, *J* = 4.1 Hz, 6H), 2.95 (s, 5H).

References

1. Frisch, M. J. . Gaussian 09. Gaussian, Inc.: Wallingford CT, 2009.
2. Becke, A. D. *J. Chem. Phys.* **1993**, *98*, 5648–5652.
3. Lee, C.; Yang, W.; Parr, R. G. *Phys. Rev. B* **1988**, *37*, 785–789.
4. Krishnan, R.; Binkley, J. S.; Seeger, R.; Pople, J. A. *J. Chem. Phys.* **1980**, *72*, 650–654.
5. Clark, T.; Chandrasekhar, J.; Spitznagel, G. W.; Schleyer, P. V. R. *J. Comput. Chem.* **1983**, *4*, 294–301.
6. Frisch, M. J.; Pople, J. A.; Binkley, J. S. *J. Chem. Phys.* **1984**, *80*, 3265–3269.
7. Sheldrick, G. M. A Short History of SHELX. *Acta Crystallogr. Sect. A Found. Crystallogr.* **2008**, *64*, 112–122.
8. Sheldrick, G. M. SHELXT - Integrated Space-Group and Crystal-Structure Determination. *Acta Crystallogr. Sect. A Found. Crystallogr.* **2015**, *71*, 3–8.
9. Dolomanov, O. V.; Bourhis, L. J.; Gildea, R. J.; Howard, J. A. K.; Puschmann, H. *J. Appl. Crystallogr.* **2009**, *42*, 339–341.
10. Antonov, A. S.; Mikshiev, V. Y.; Pozharskii, A. F.; Ozeryanskii, V. A. *Synth.* **2014**, *46*, 3273–3282.
11. Antonov, A. S.; Pozharskii, A. F.; Ozeryanskii, V. A.; Filarowski, A.; Suponitsky, K. Y.; Tolstoy, P. M.; Vovk, M. *Dalt. Trans.* **2015**, *44*, 17756–17766.
12. Povalyakhina, M. A.; Antonov, A. S.; Dyablo, O. V.; Ozeryanskii, V. A.; Pozharskii, A. F. *J. Org. Chem.* **2011**, *76*, 7157–7166.

Copies of ^{1}H and ^{13}C NMR spectra

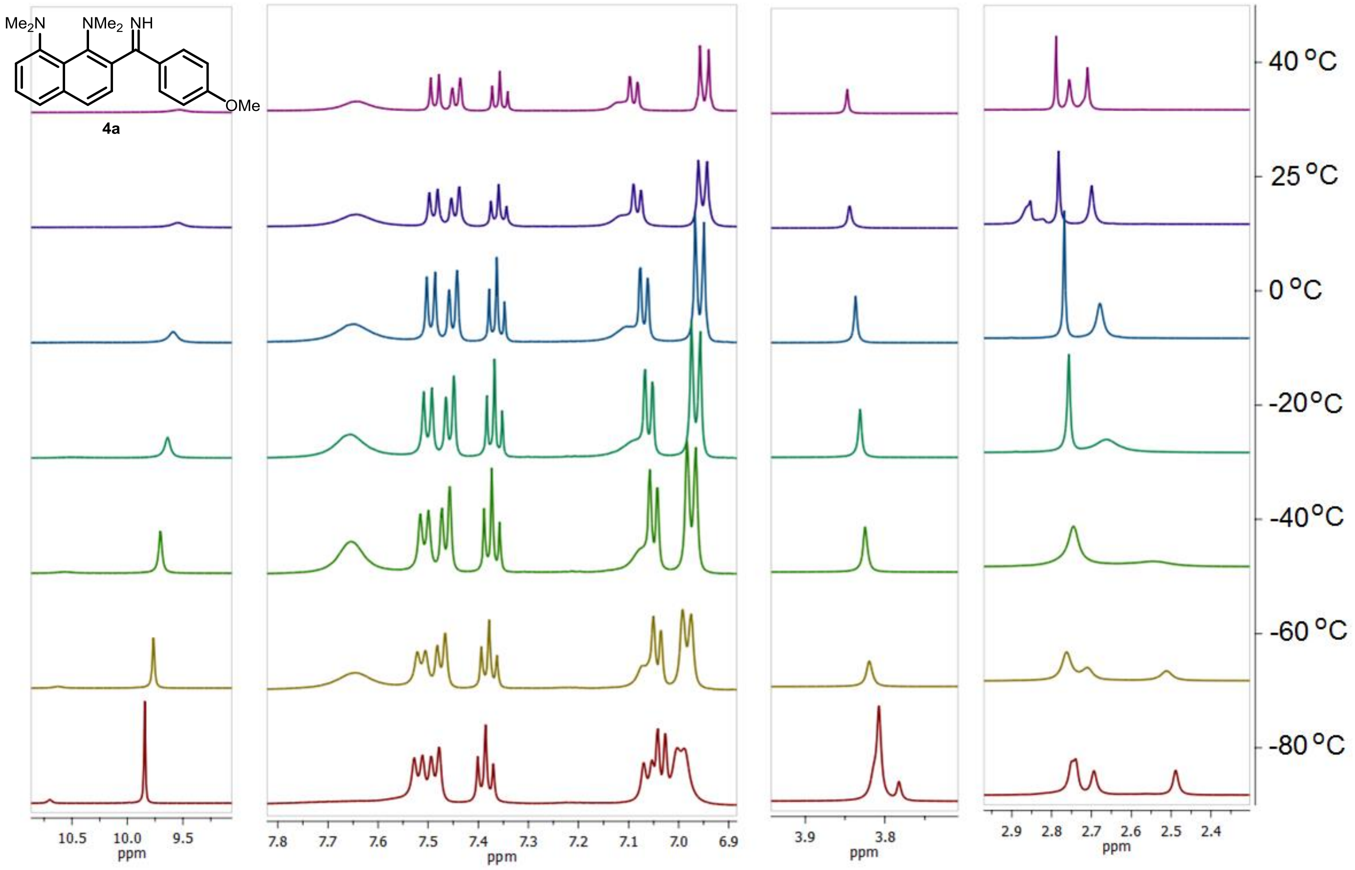

Figure S1. Temperature depending ^{1}H NMR spectra for compound **4a**, acetone-d_6, 500 MHz.

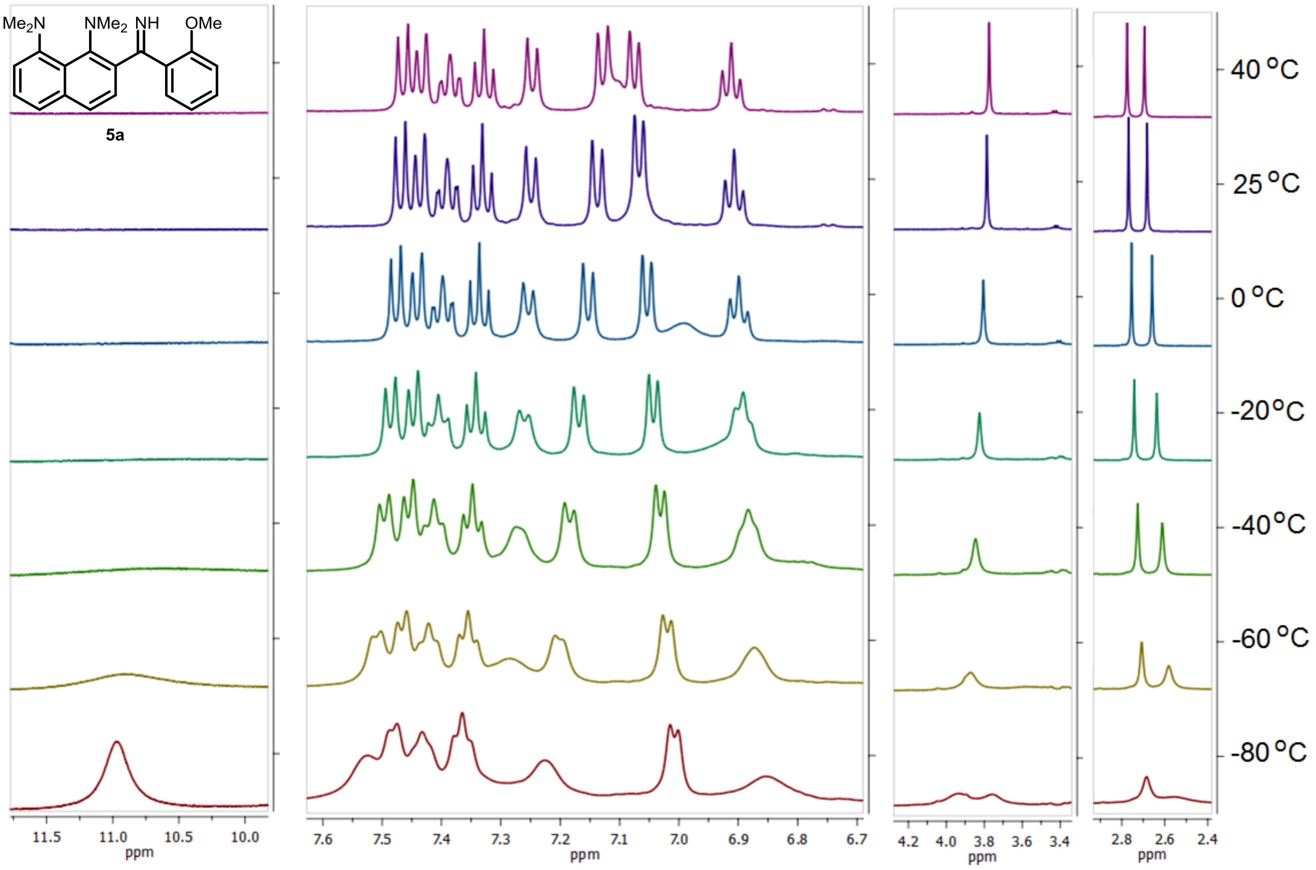

Figure S2. Temperature depending ^{1}H NMR spectra for compound **5a**, acetone-d_6, 500 MHz.

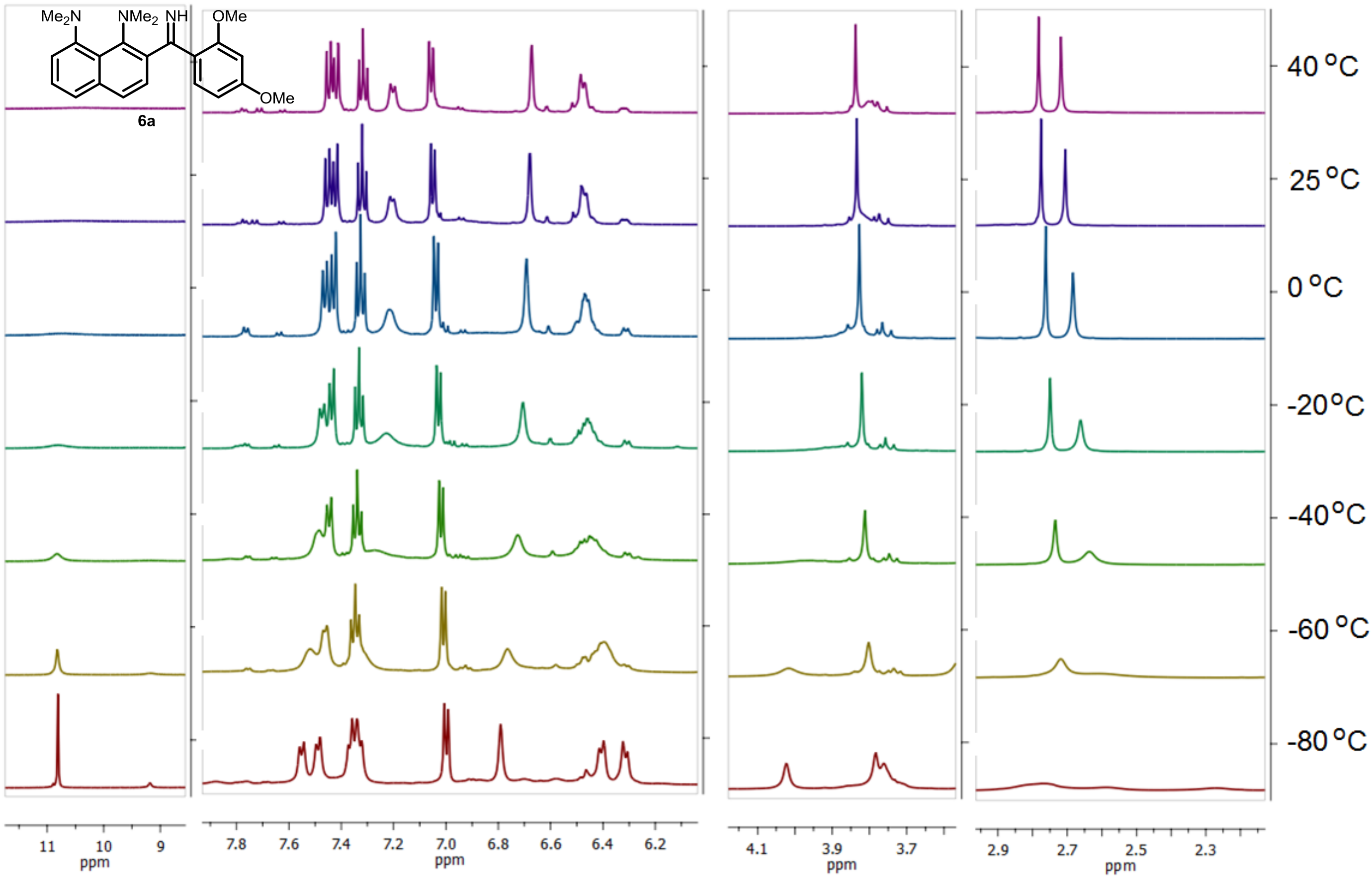

Figure S3. Temperature depending ^{1}H NMR spectra for compound **6a**, acetone-d_6, 500 MHz.

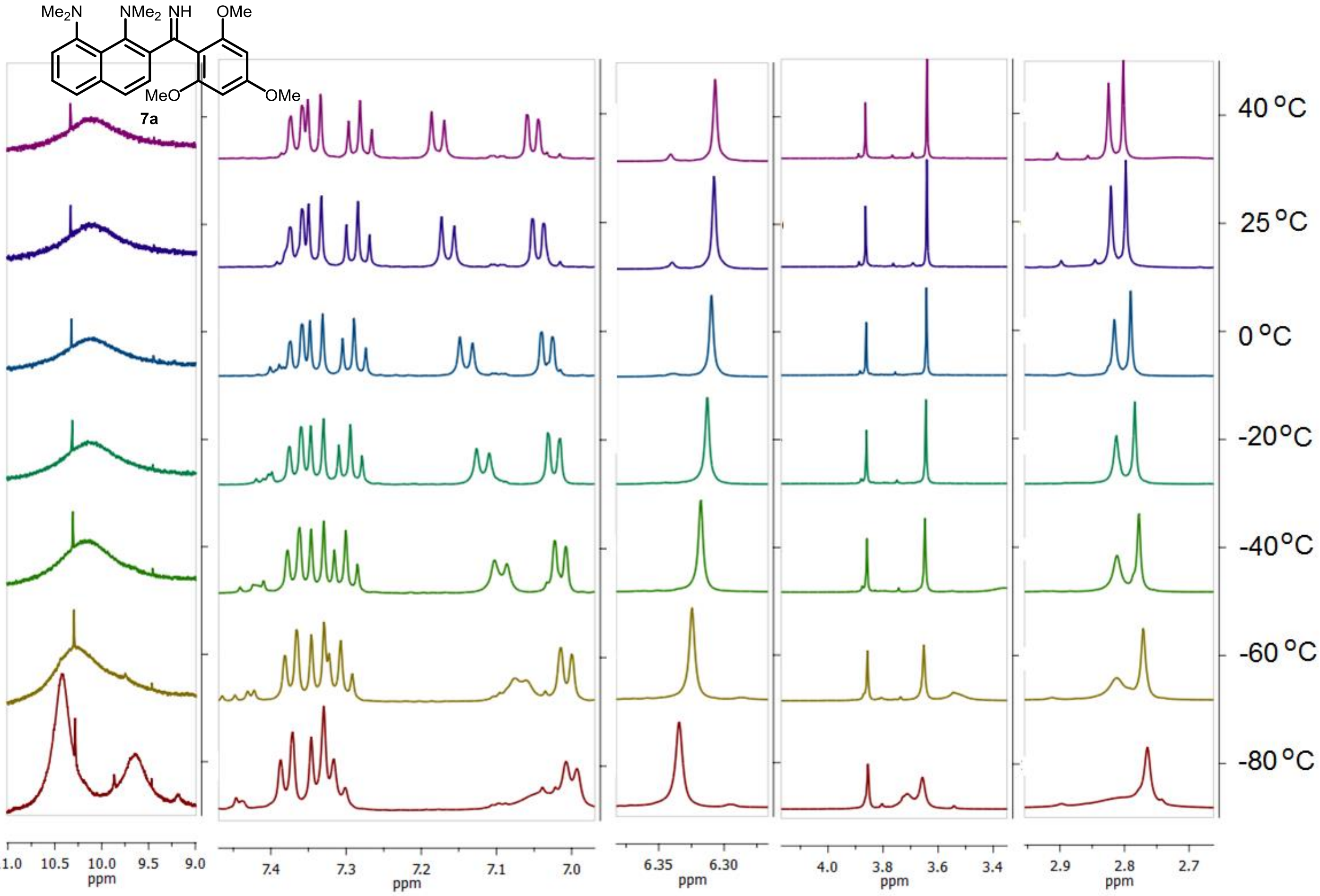

Figure S4. Temperature depending ^{1}H NMR spectra for compound **7a**, acetone-d_6, 500 MHz.

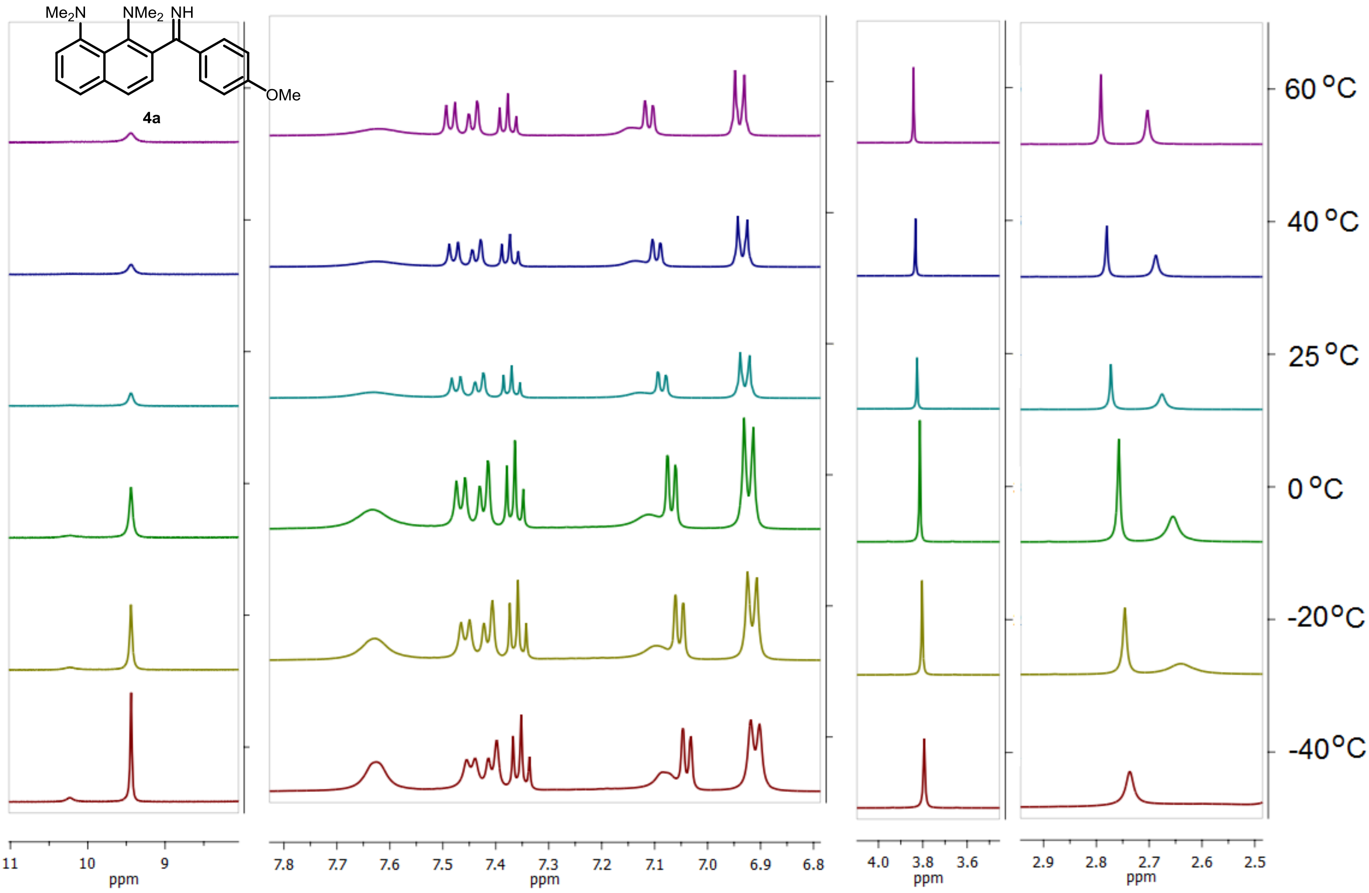

Figure S5. Temperature depending ^{1}H NMR spectra for compound **4a**, CD_3CN, 500 MHz.

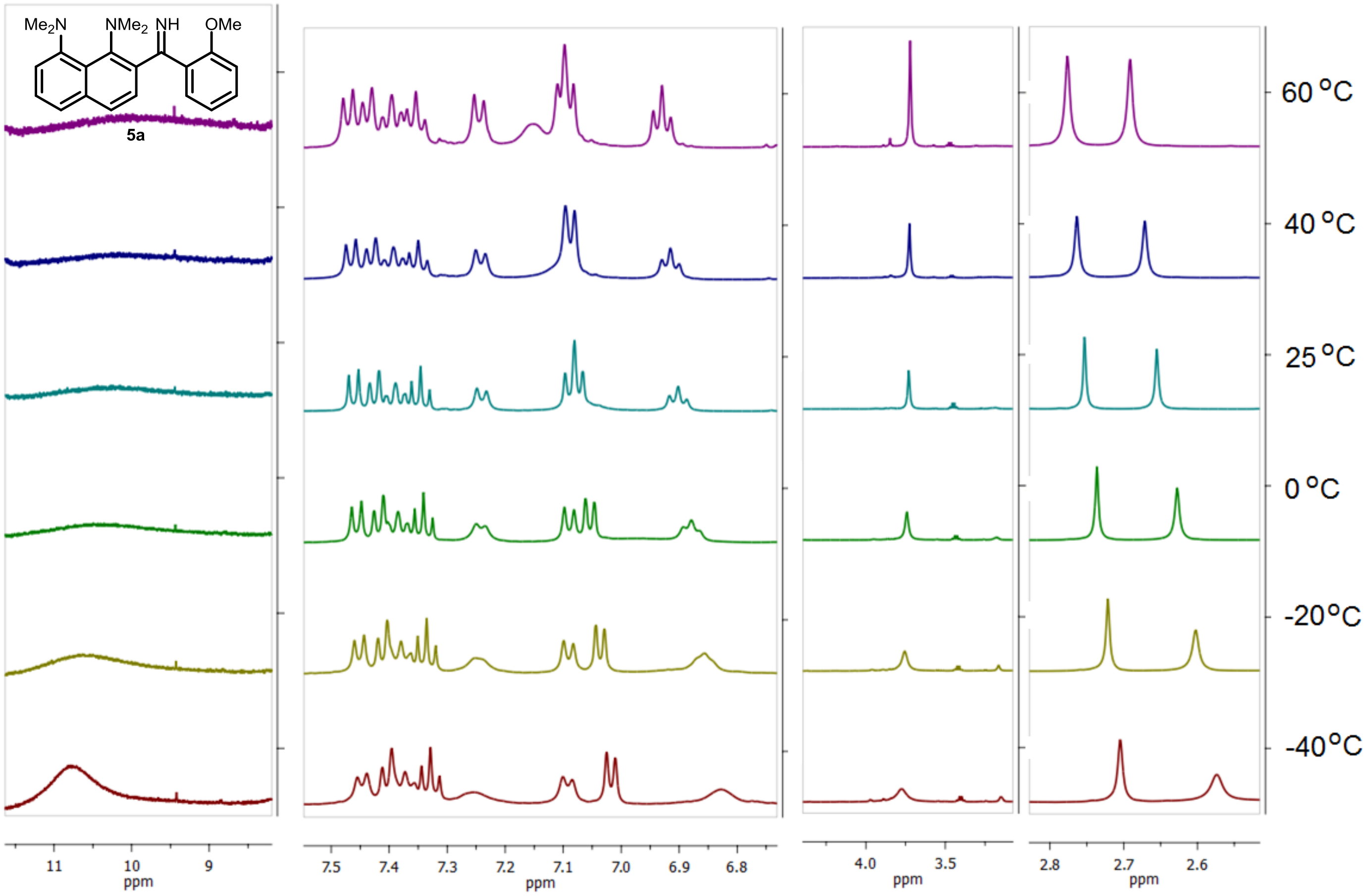

Figure S6. Temperature depending ^{1}H NMR spectra for compound **5a**, CD_3CN, 500 MHz.

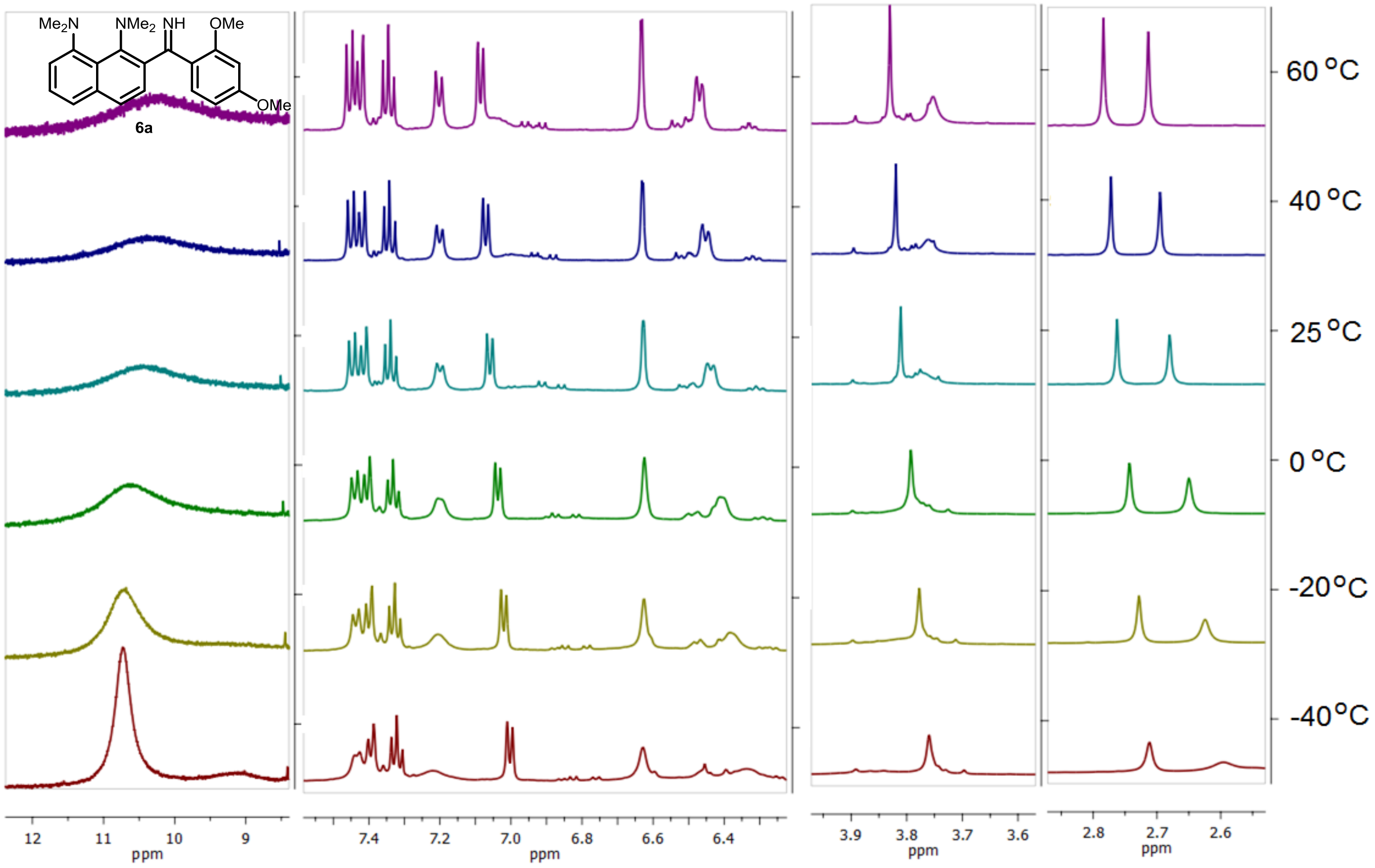

Figure S7. Temperature depending ^{1}H NMR spectra for compound **6a**, CD_3CN, 500 MHz.

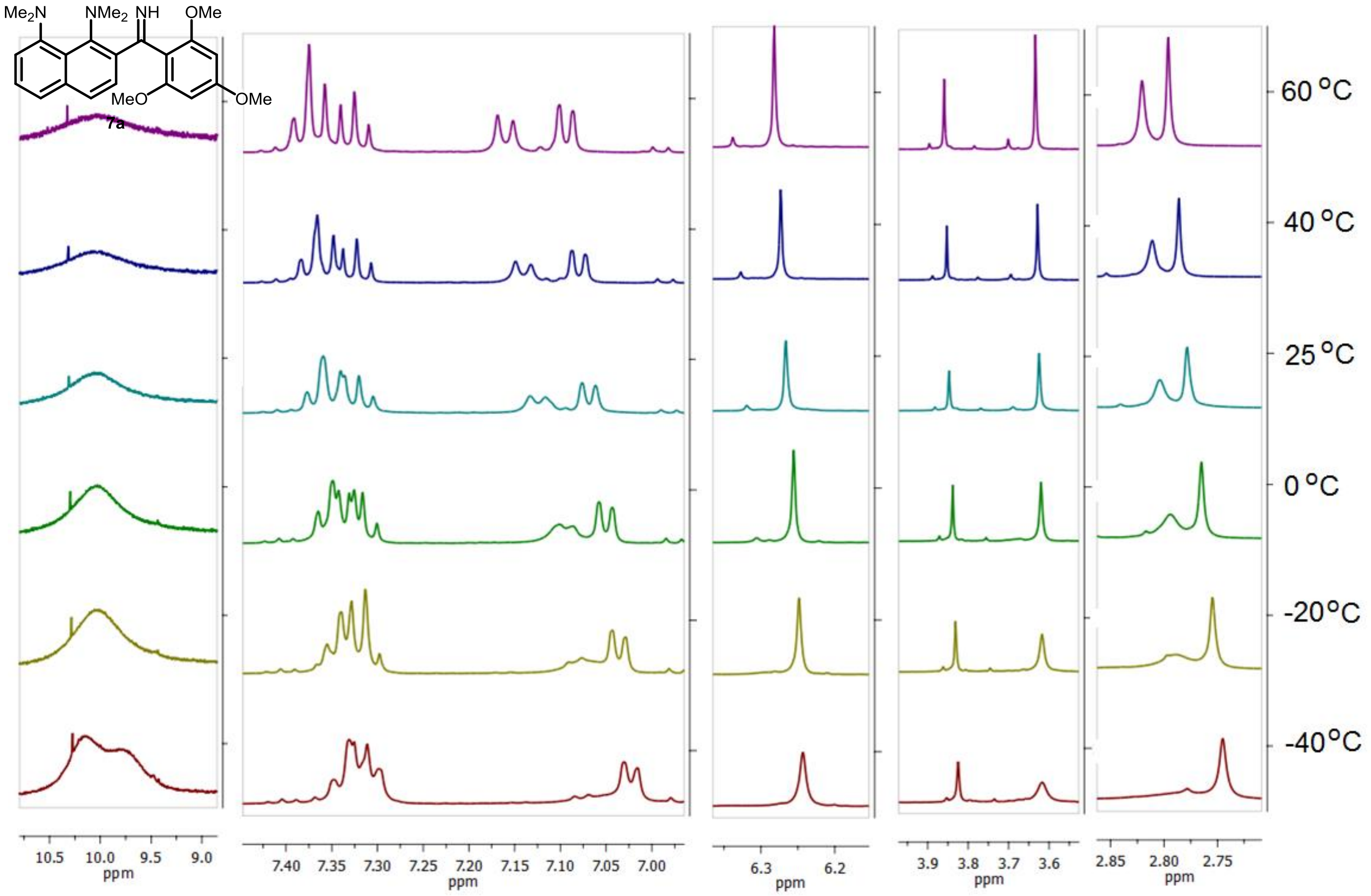

Figure S8. Temperature depending ^{1}H NMR spectra for compound **7a**, CD_3CN, 500 MHz.

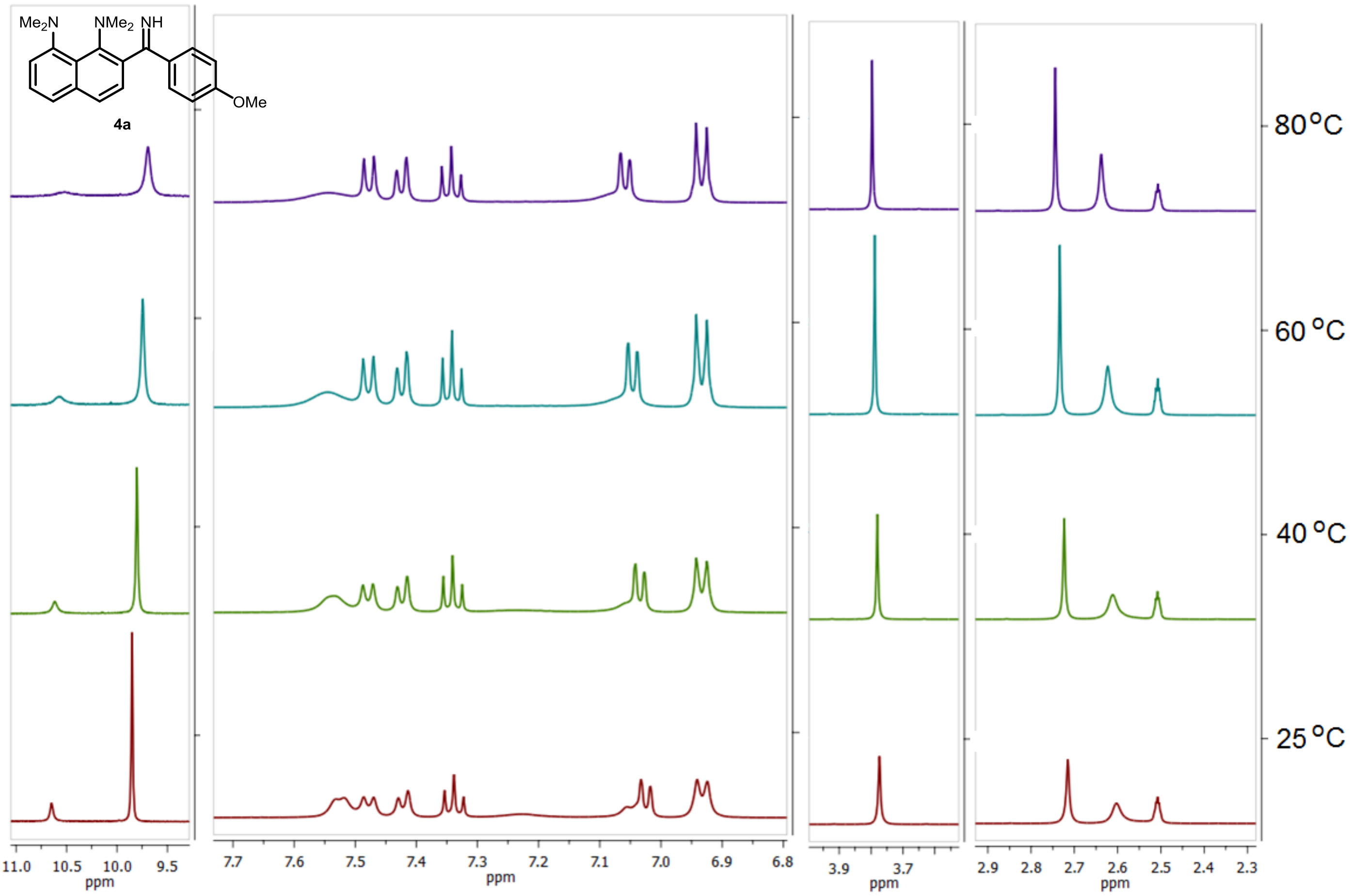

Figure S9. Temperature depending ^{1}H NMR spectra for compound **4a**, DMSO-d_6, 500 MHz.

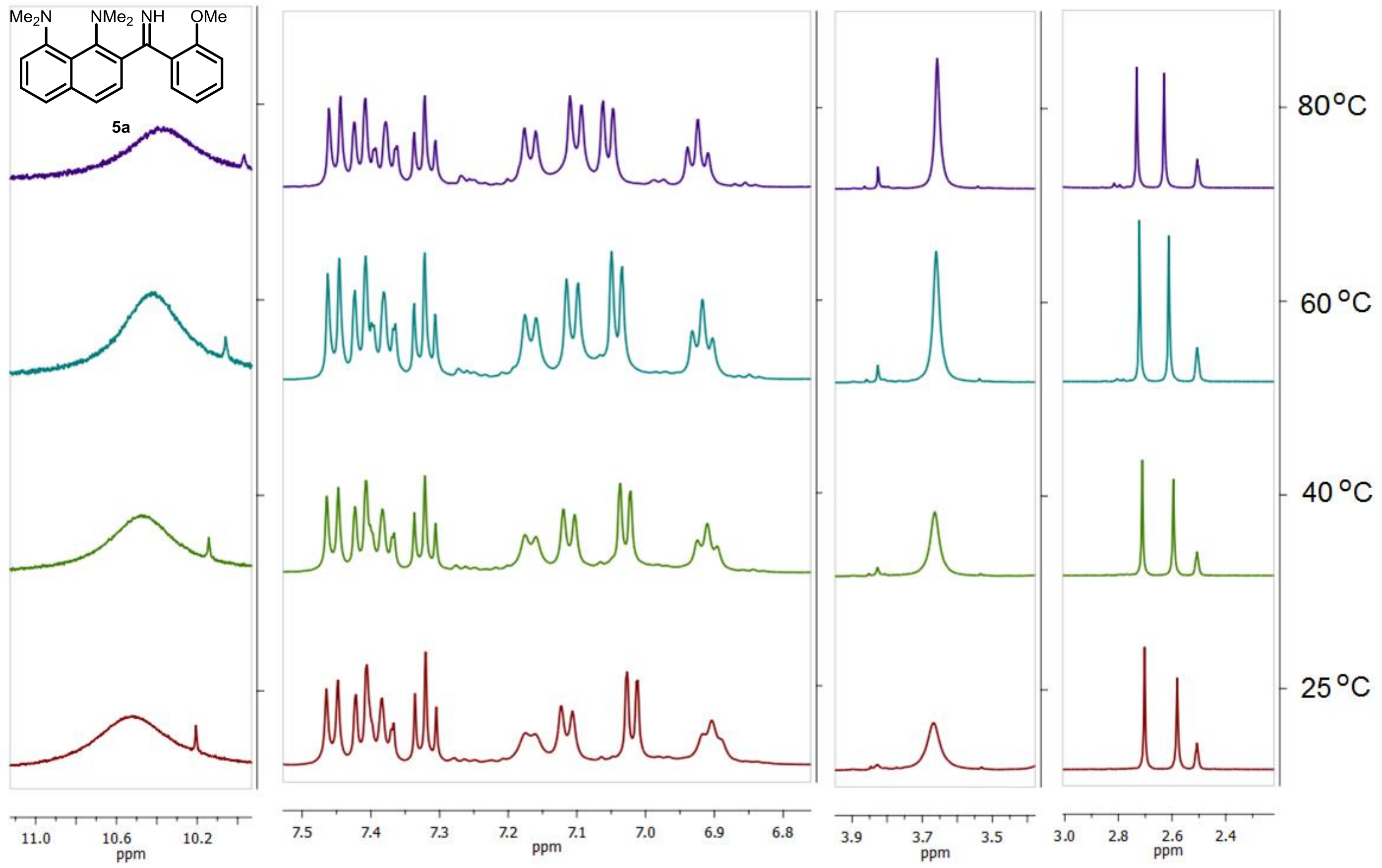

Figure S10. Temperature depending ^{1}H NMR spectra for compound **5a**, DMSO-d_6, 500 MHz.

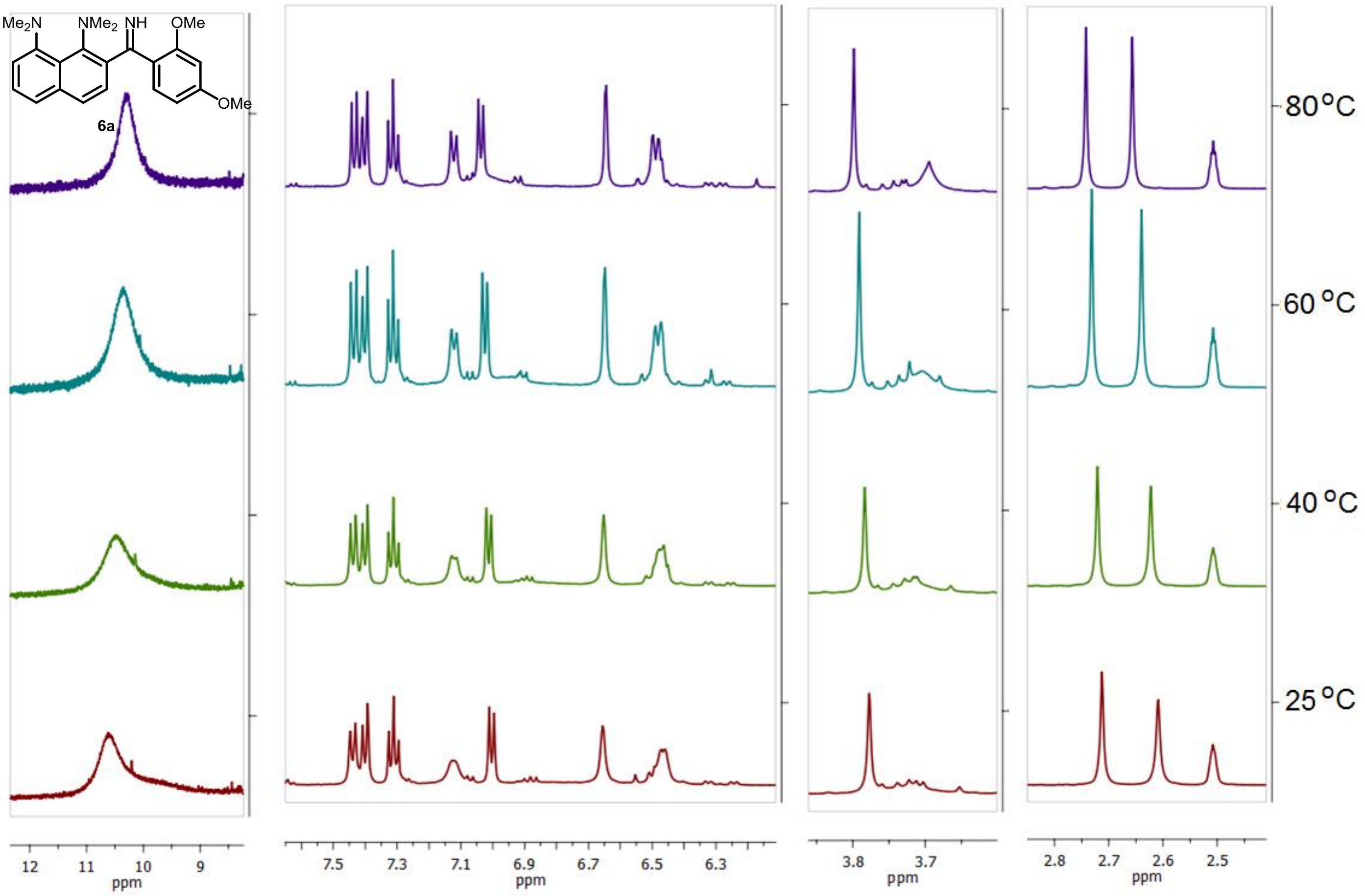

Figure S11. Temperature depending ^{1}H NMR spectra for compound **6a**, DMSO-d_6, 500 MHz.

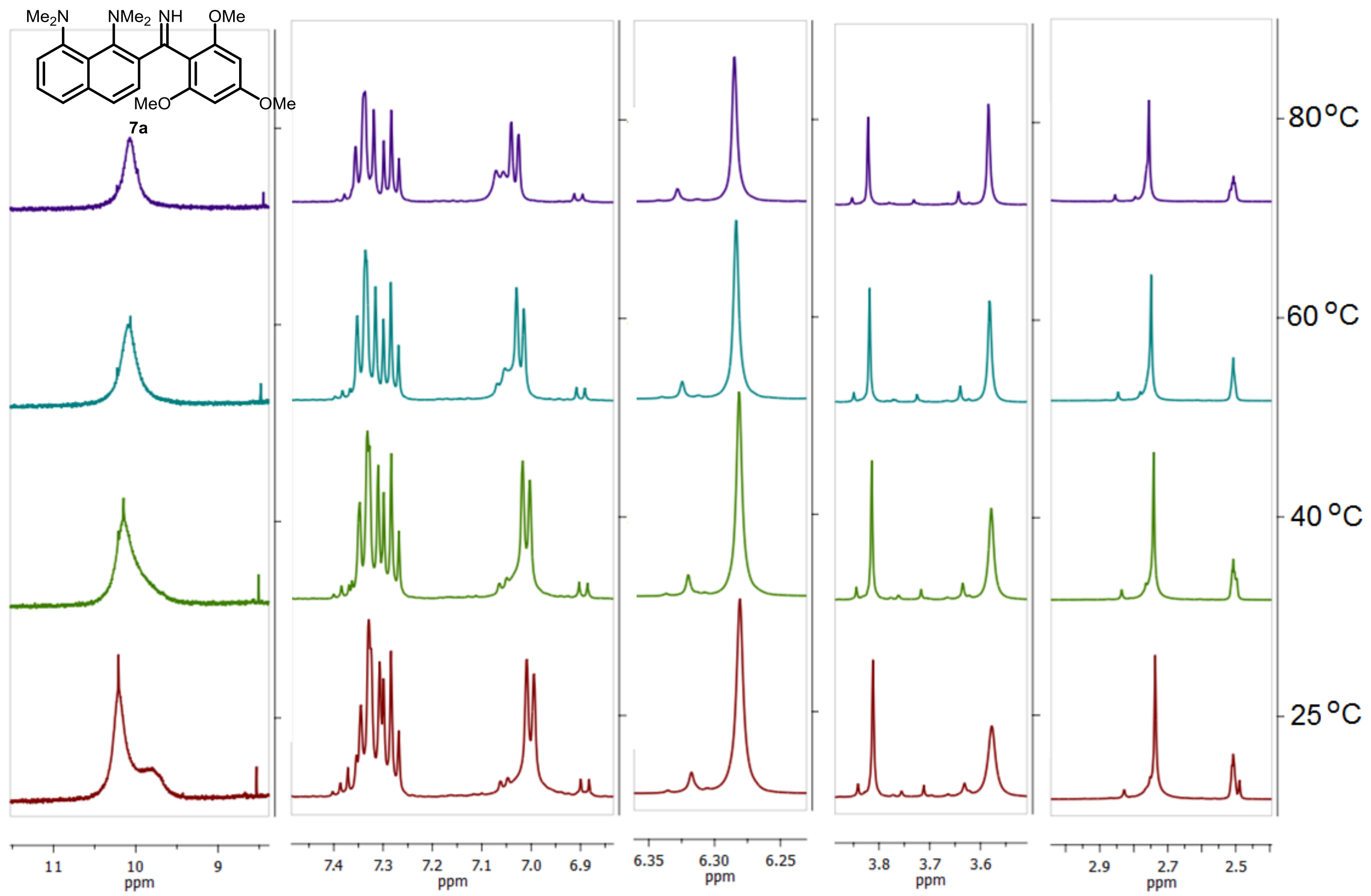

Figure S12. Temperature depending ^{1}H NMR spectra for compound **7a**, DMSO-d_6, 500 MHz.

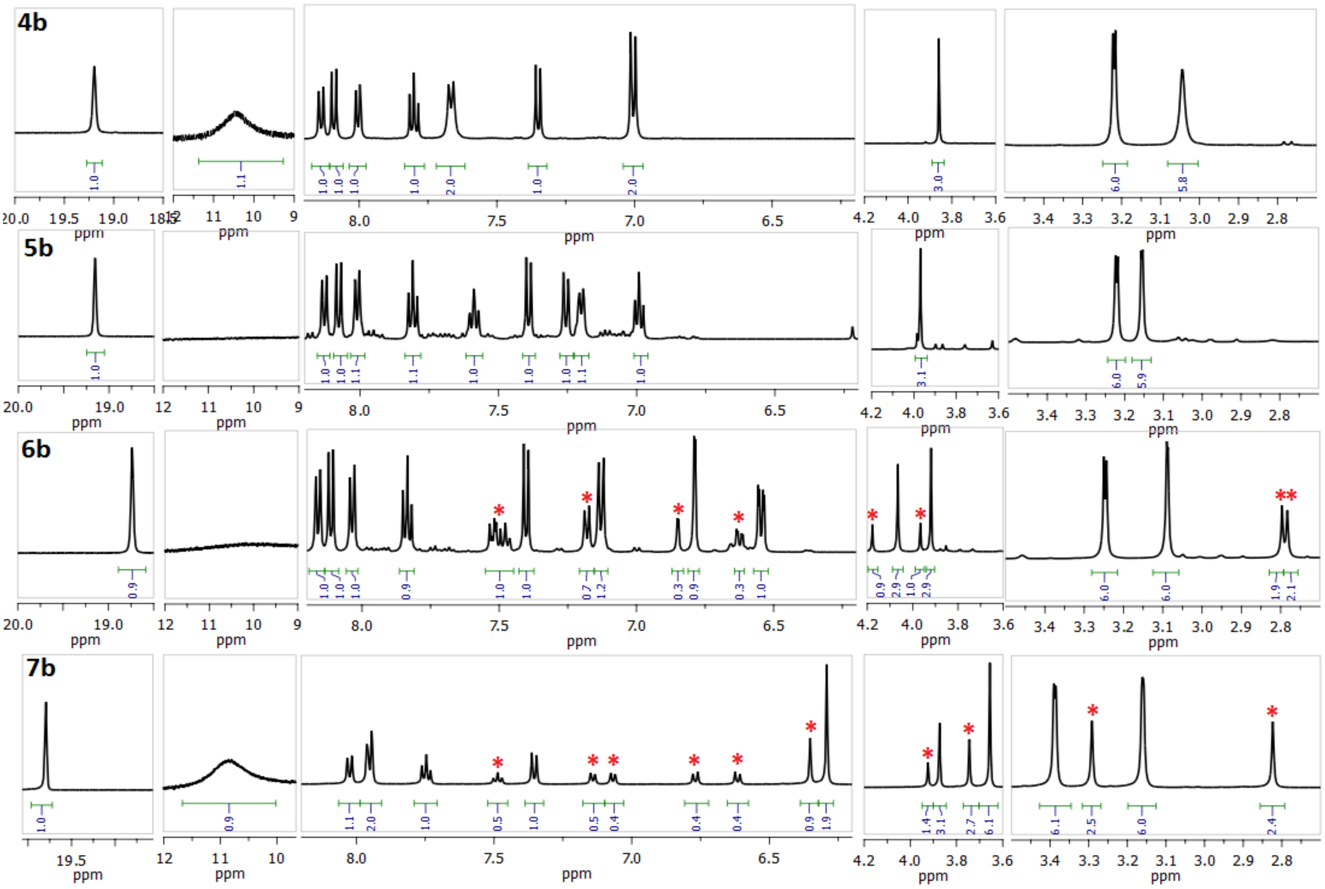

Figure S13. ^{1}H NMR spectra of imines **4b-7b** in CD_3CN, 500 MHz, 25 °C (signals of forms protonated to imino function are marked with *).

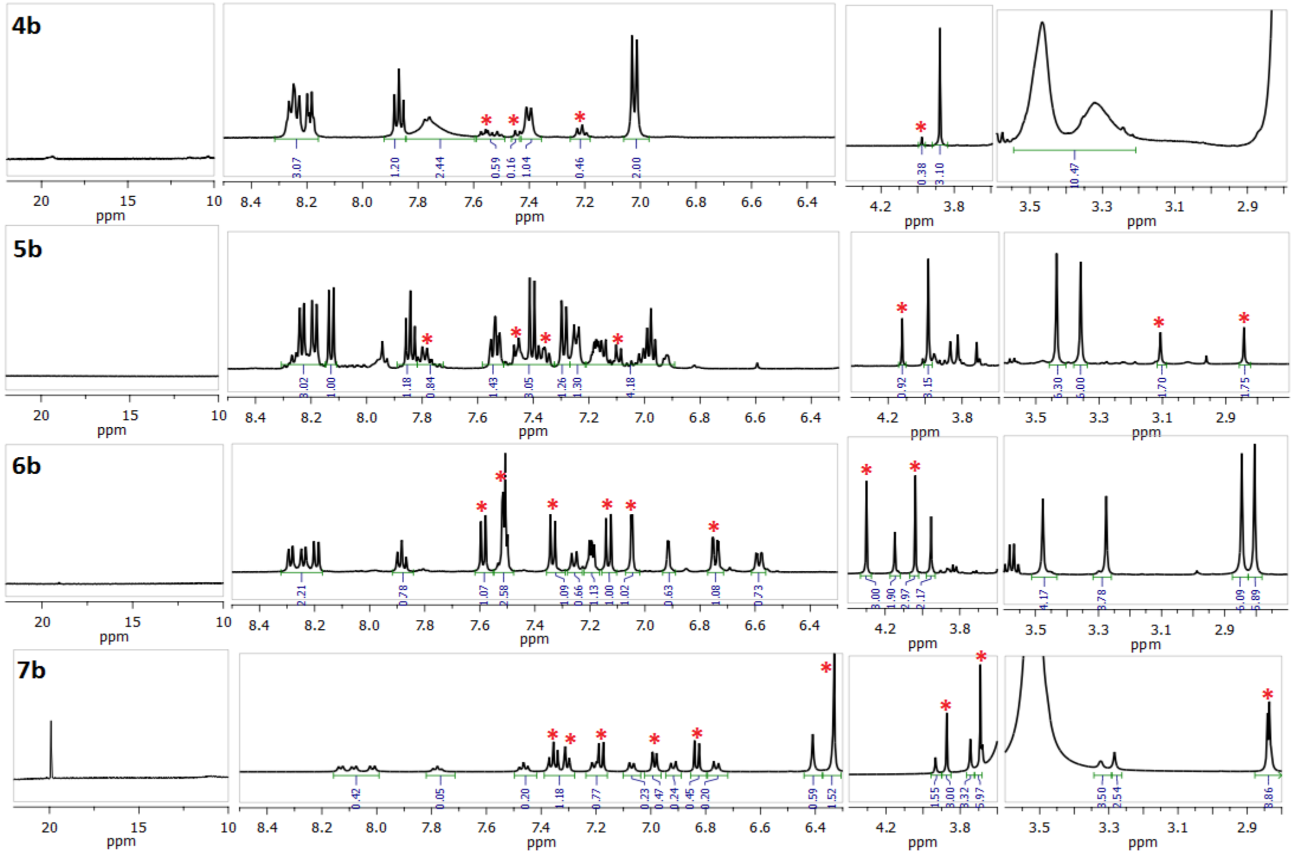

Figure S14. ^{1}H NMR spectra of imines **4b-7b** in acetone-*d*6, 25 °C, 500 MHz (signals of forms protonated to imino function are marked with *)

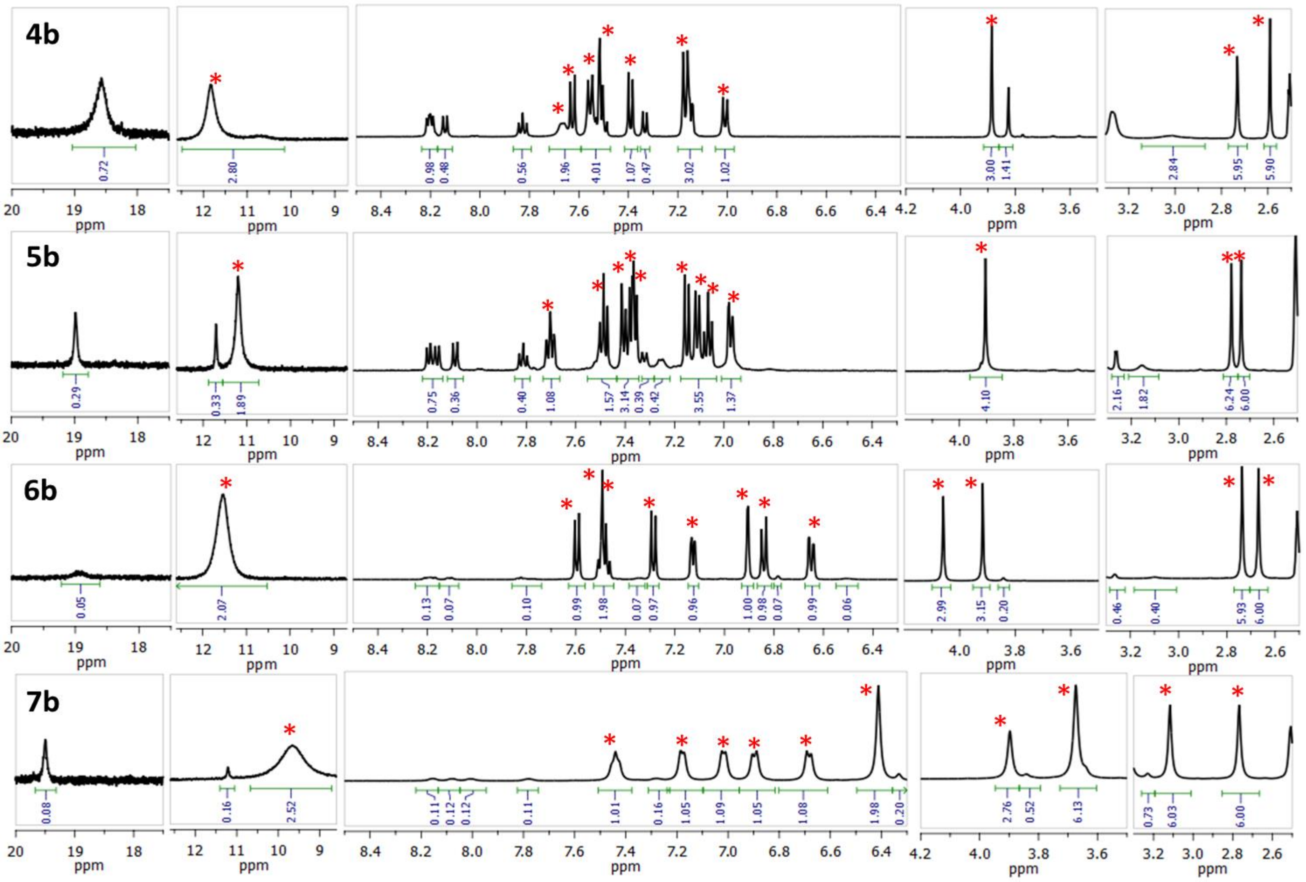

Figure S15. ^{1}H NMR spectra of imines **4b-7b** in DMSO-d_6, 500 MHz, 25 °C(signals of forms protonated to imino function are marked with *).

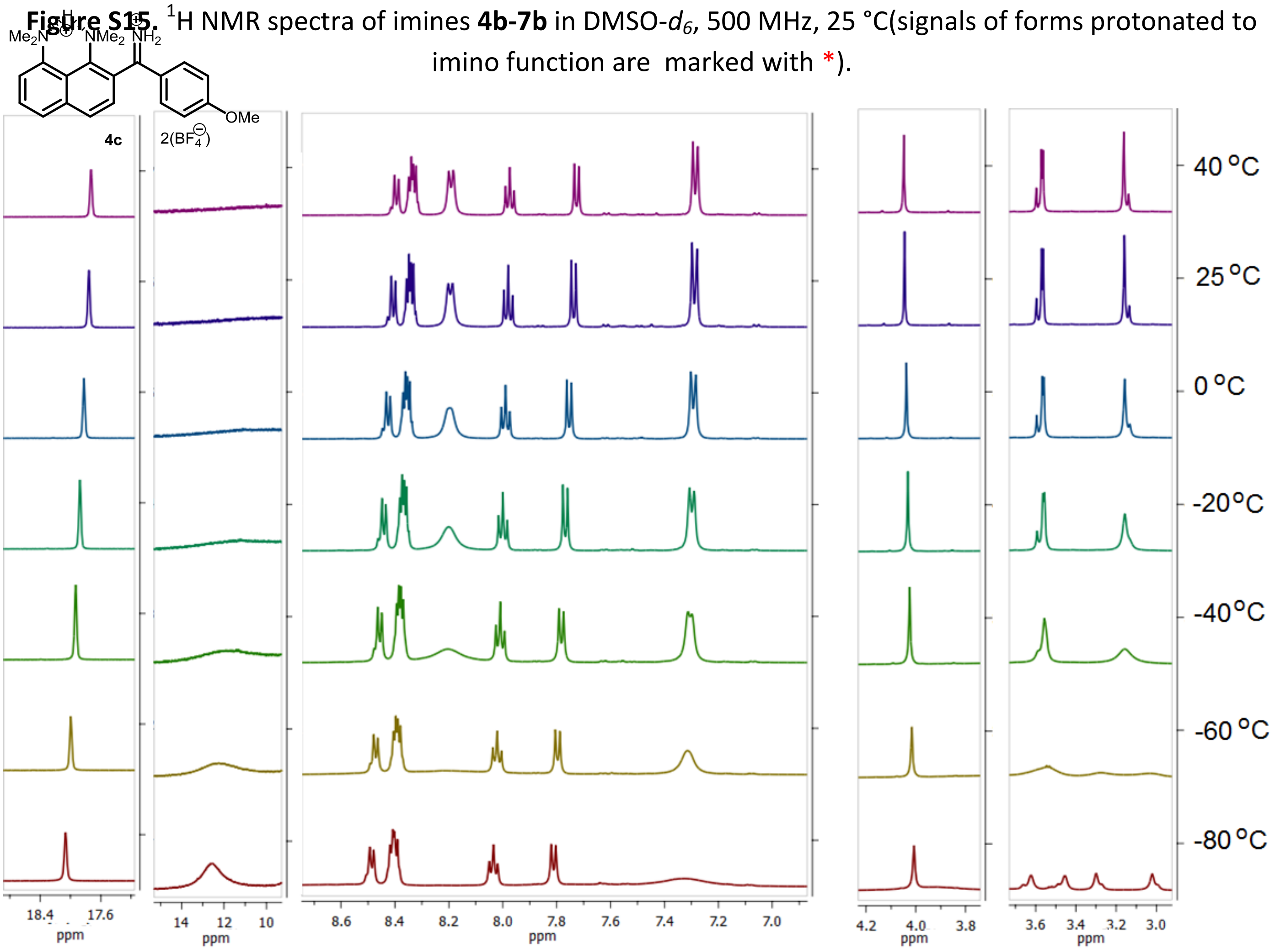

Figure S16. Temperature depending ^{1}H NMR spectra for compound **4c**, acetone-d_6, 500 MHz.

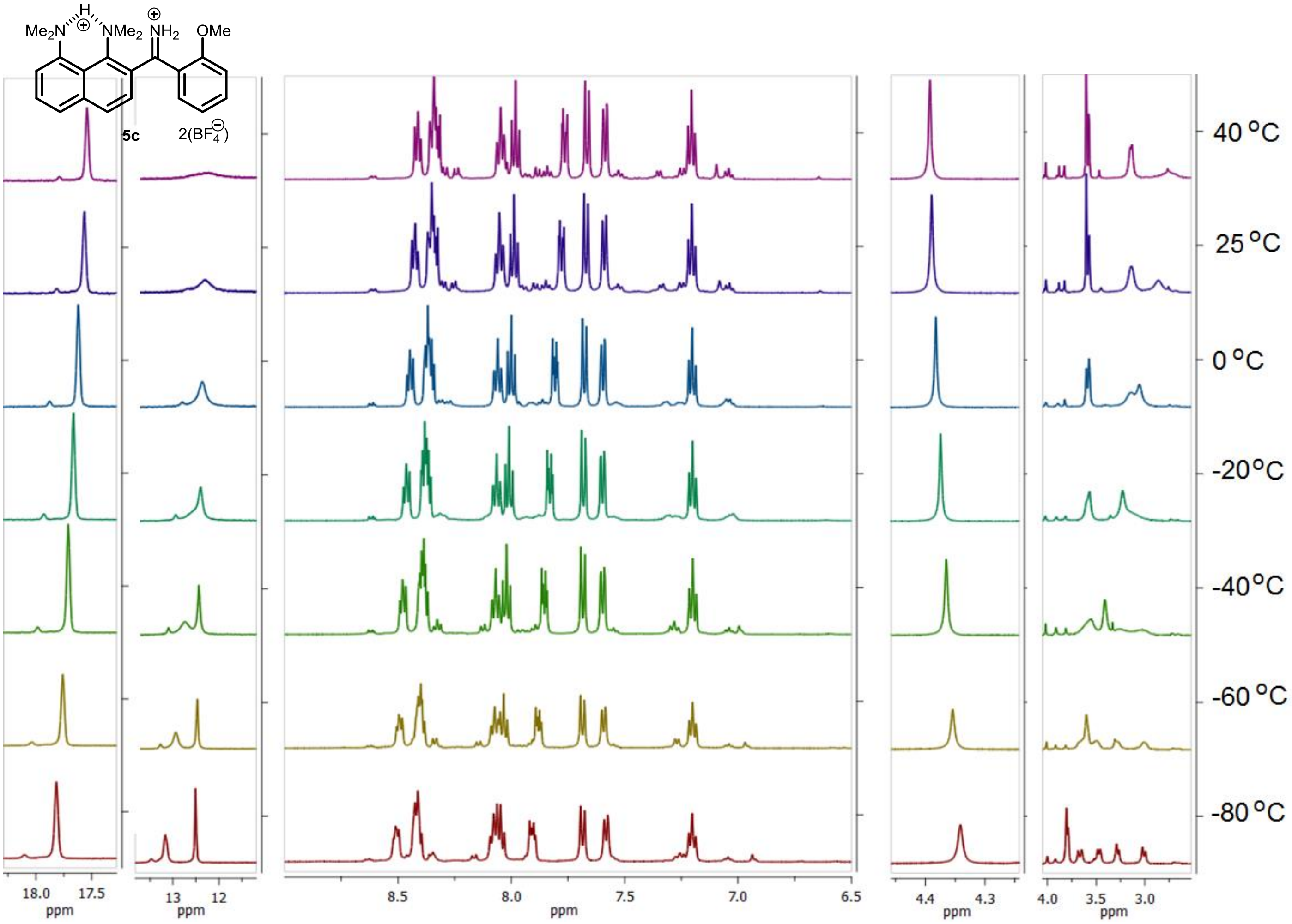

Figure S17. Temperature depending ^{1}H NMR spectra for compound **5c**, acetone-d_6, 500 MHz.

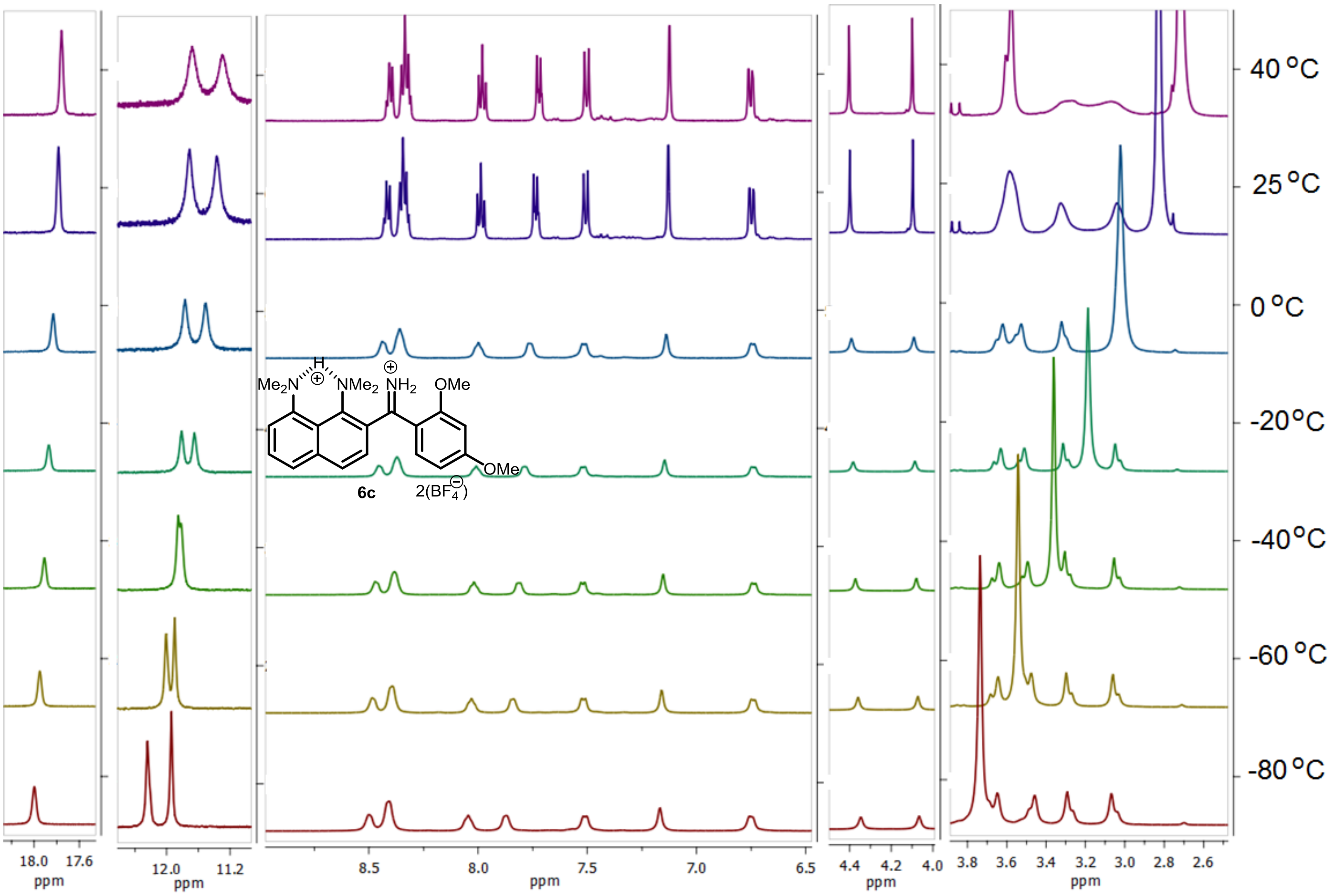

Figure S18. Temperature depending ^{1}H NMR spectra for compound **6c**, acetone-d_6, 500 MHz.

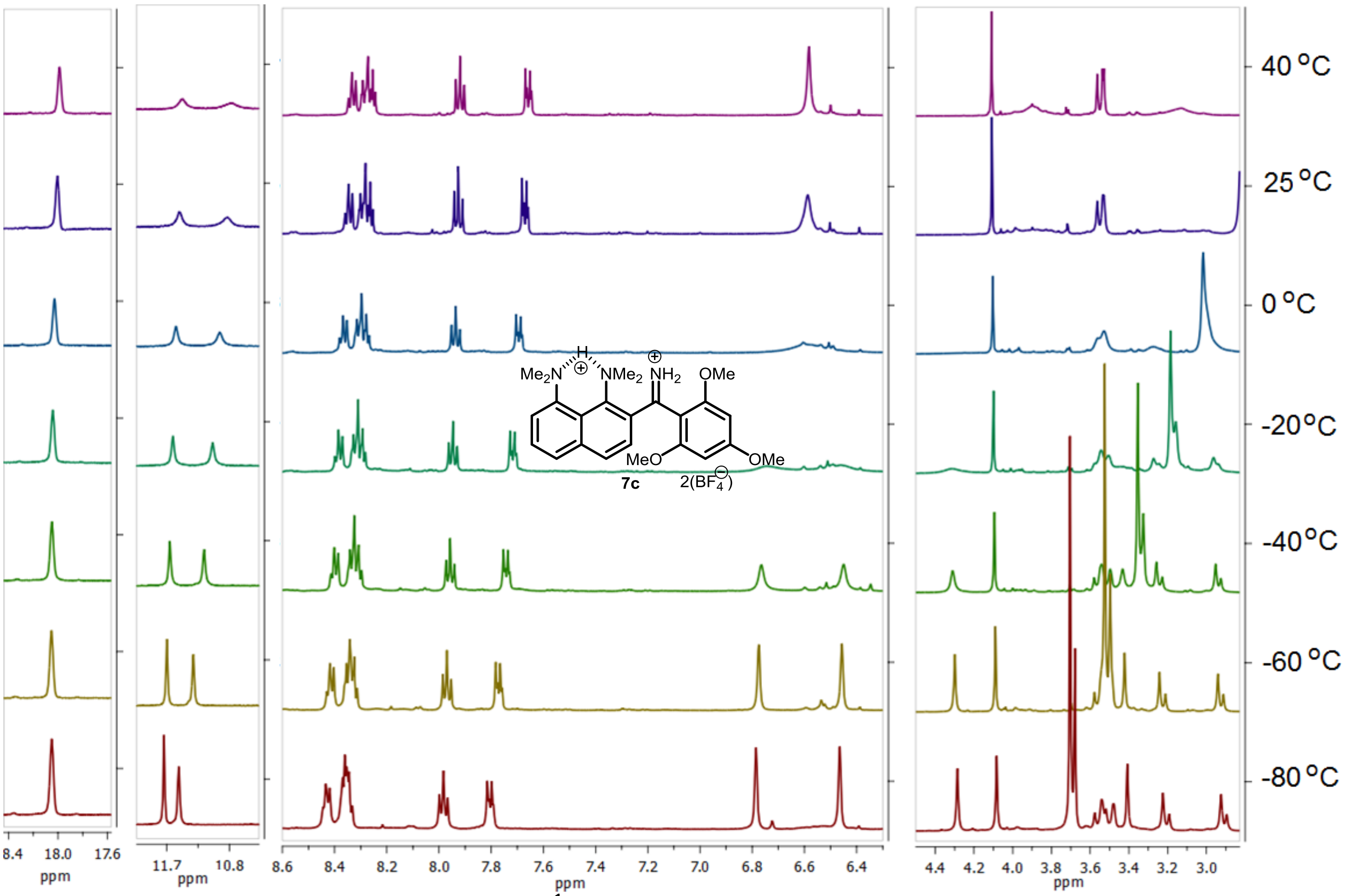

Figure S19. Temperature depending ^{1}H NMR spectra for compound **7c**, acetone-d_6, 500 MHz.

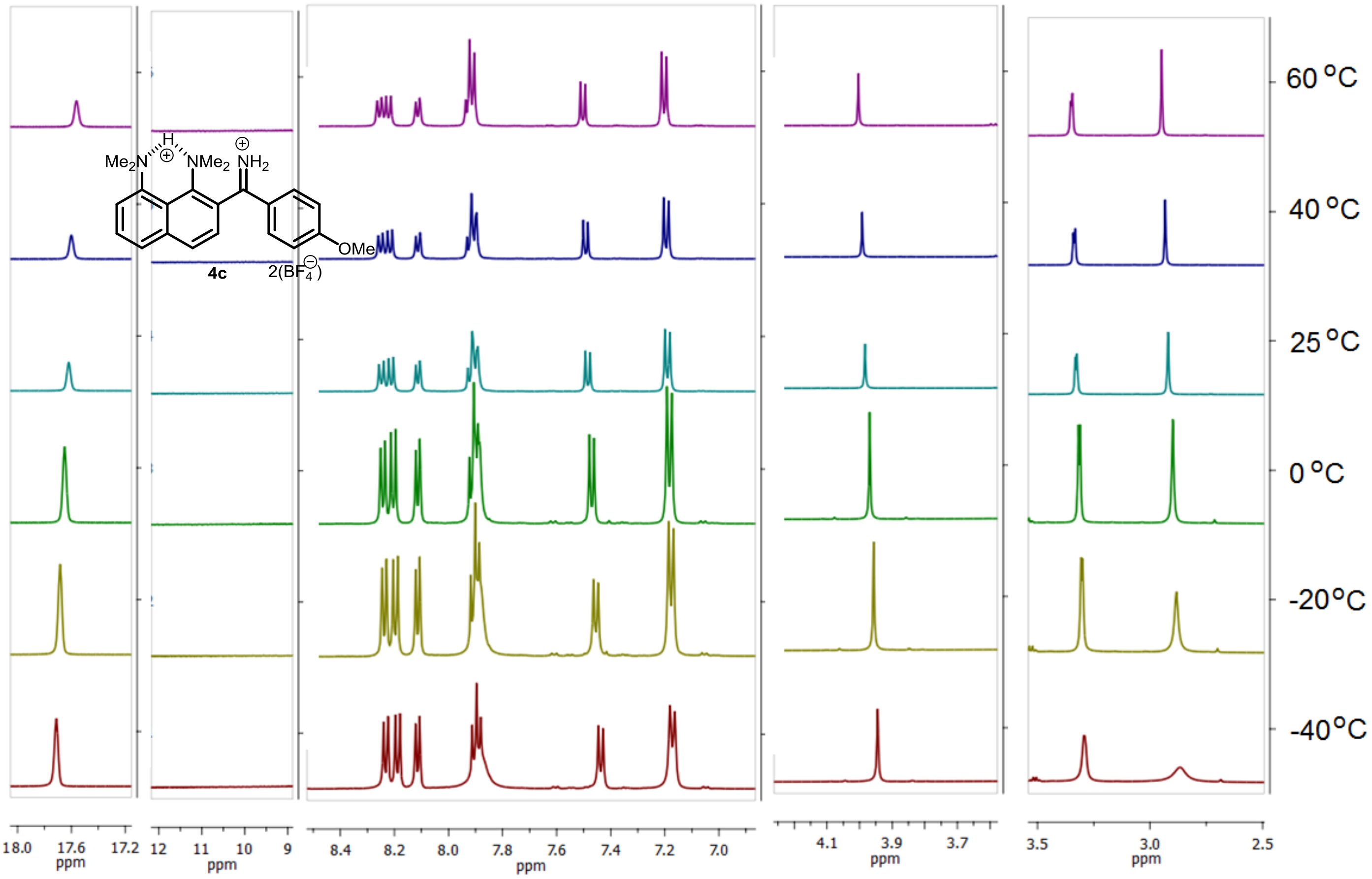

Figure S20. Temperature depending ^{1}H NMR spectra for compound **4c**, CD_3CN, 500 MHz.

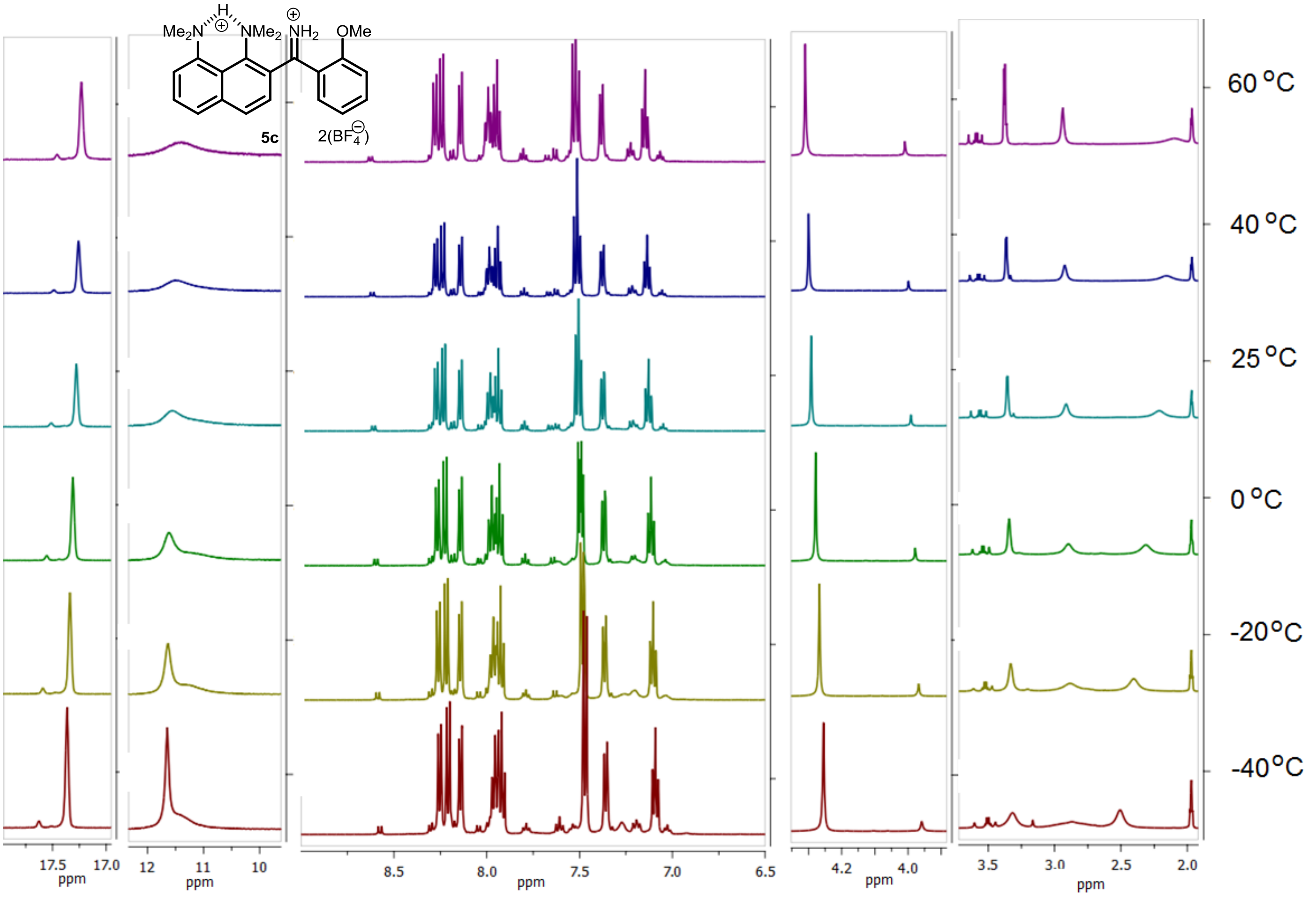

Figure S21. Temperature depending ^{1}H NMR spectra for compound **5c**, CD_3CN, 500 MHz.

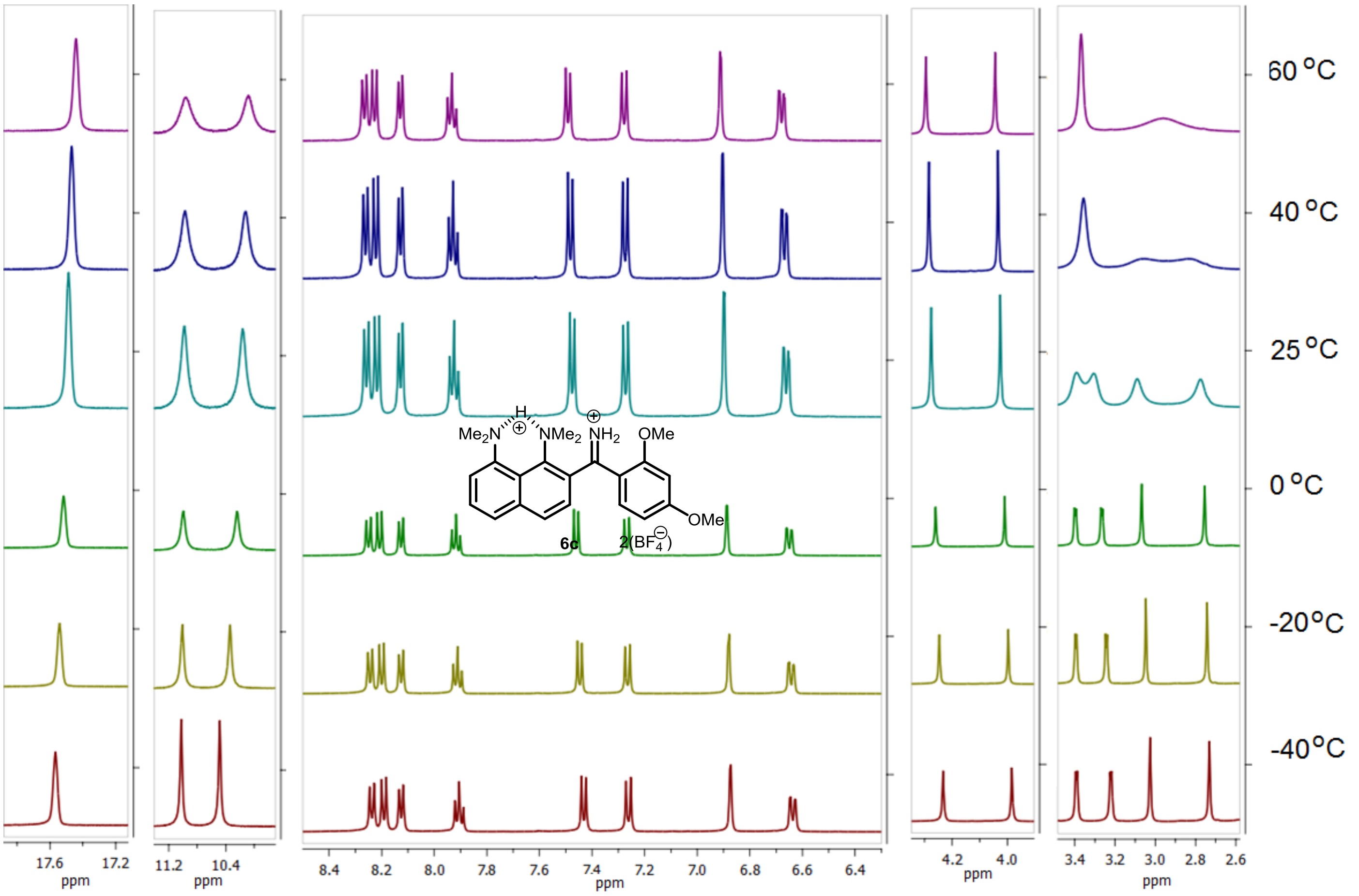

Figure S22. Temperature depending ^{1}H NMR spectra for compound **6c**, CD_3CN, 500 MHz.

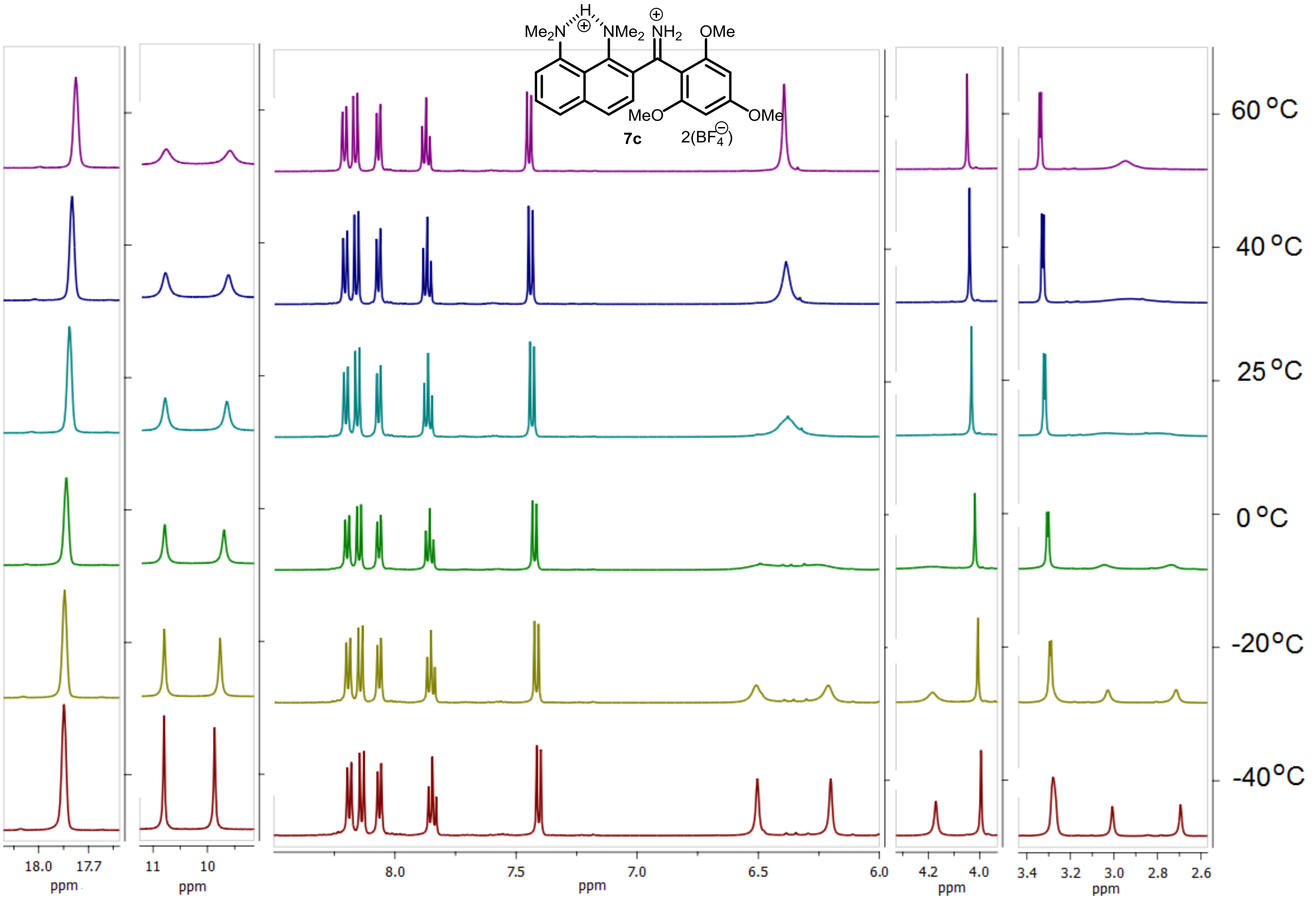

Figure S23. Temperature depending ^{1}H NMR spectra for compound **7c**, CD_3CN, 500 MHz, 25 °C.

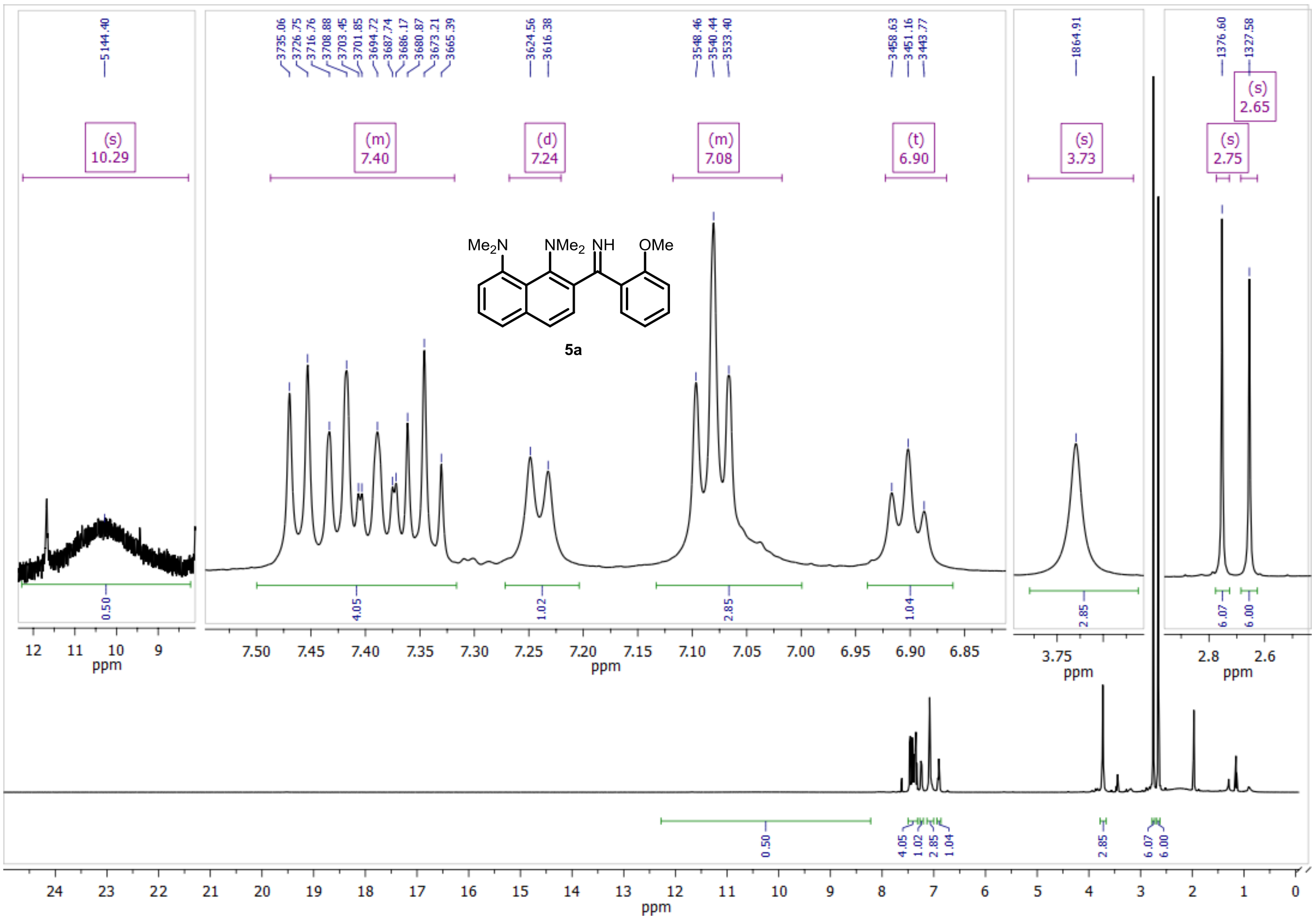

Figure S24. ^{1}H NMR spectrum of **5a**, CD_3CN, 500 MHz, 25 °C.

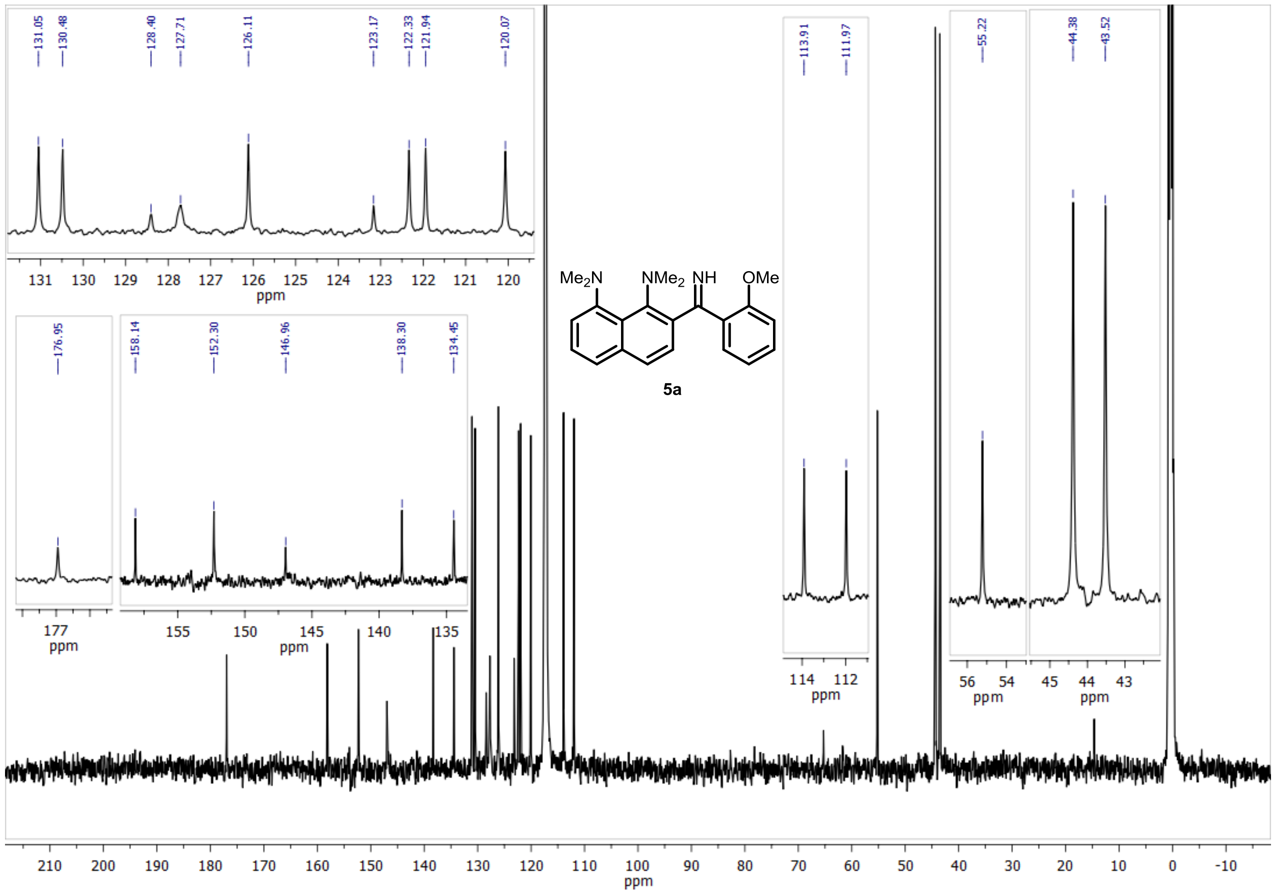

Figure S25. ^{13}C NMR spectrum of **5a**, CD_3CN, 126 MHz, 25 °C.

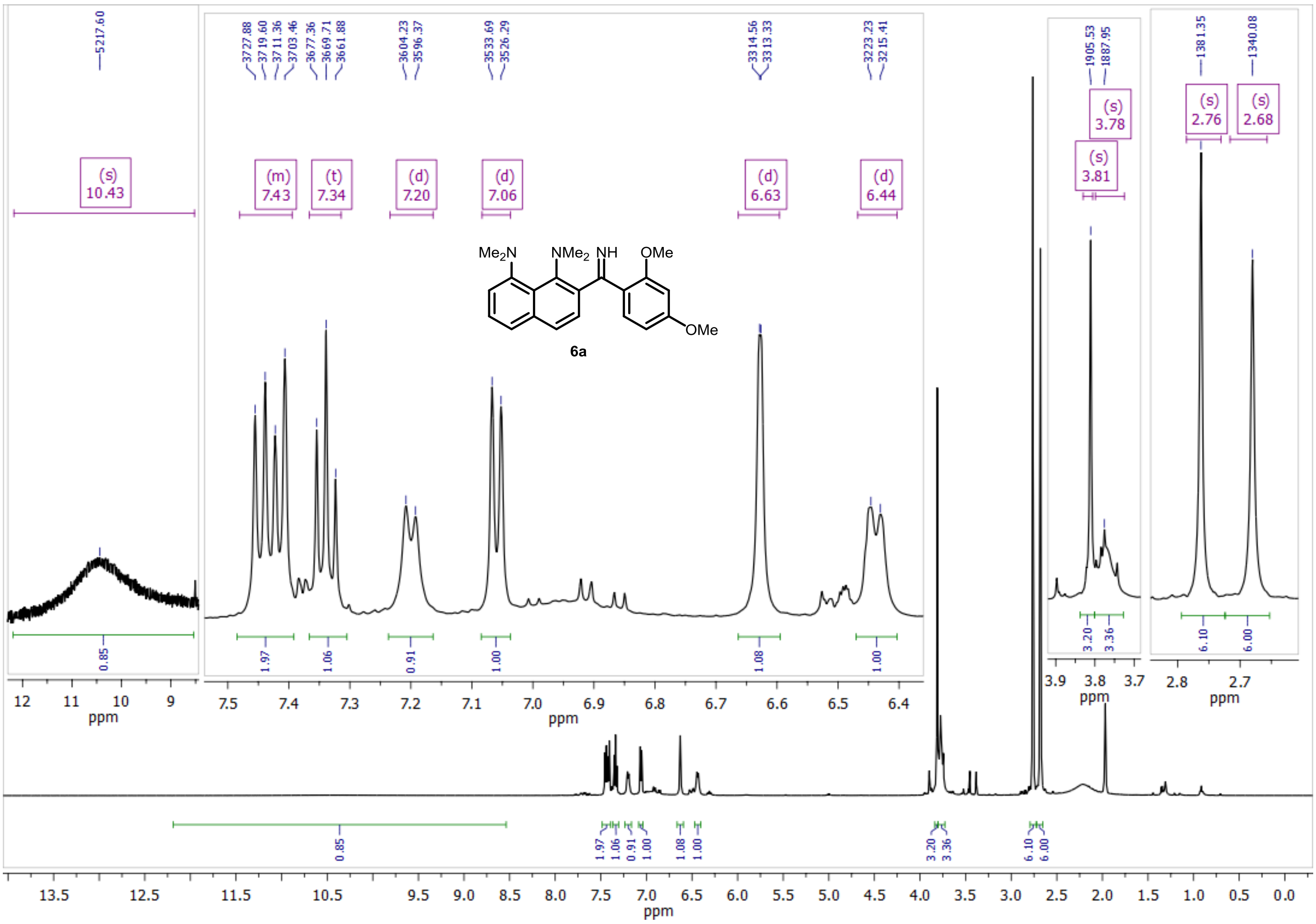

Figure S26. ^{1}H NMR spectrum of **6a**, CD_3CN, 500 MHz, 25 °C.

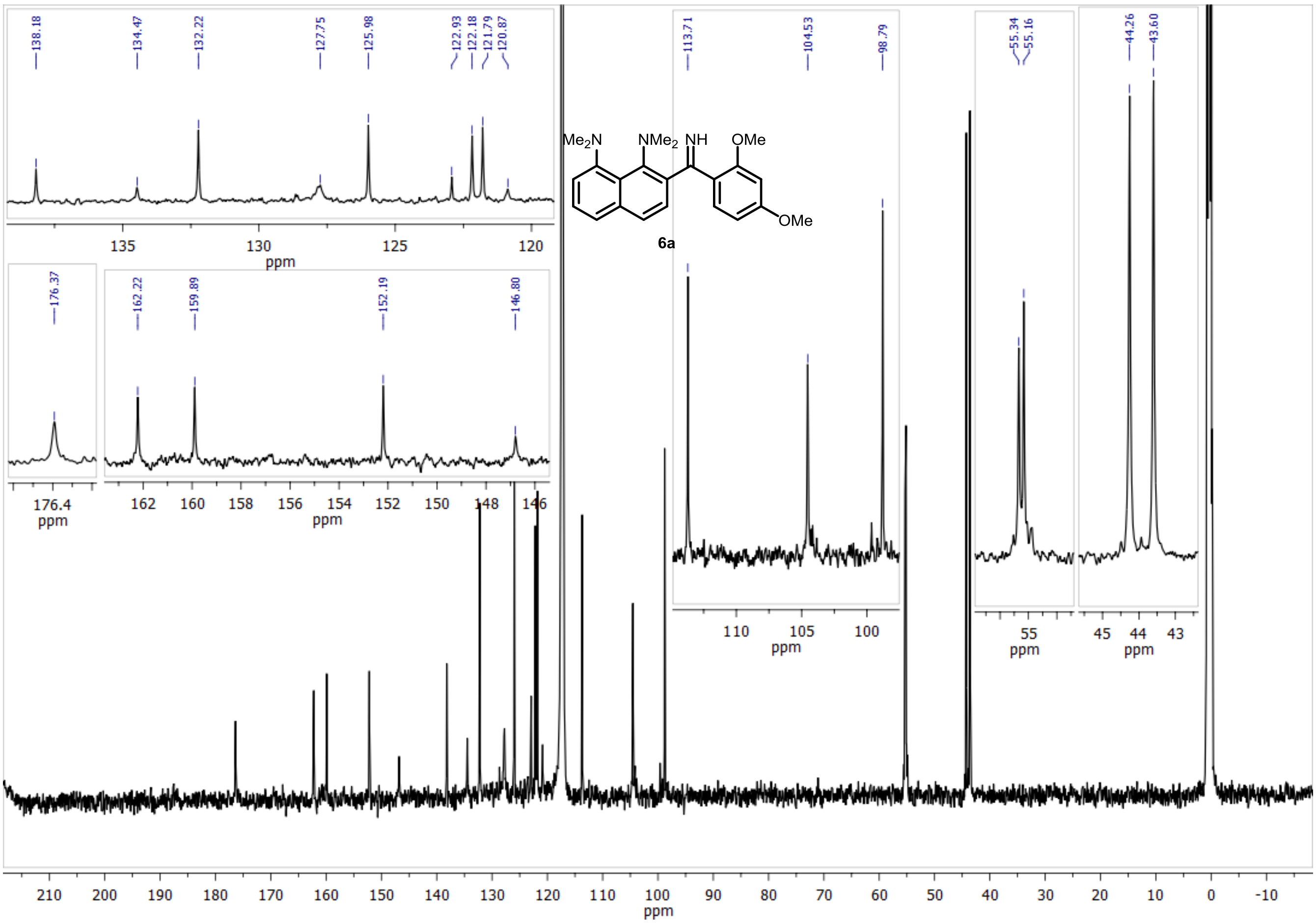

Figure S27. ^{13}C NMR spectrum of **6a**, CD_3CN, 126 MHz, 25 °C.

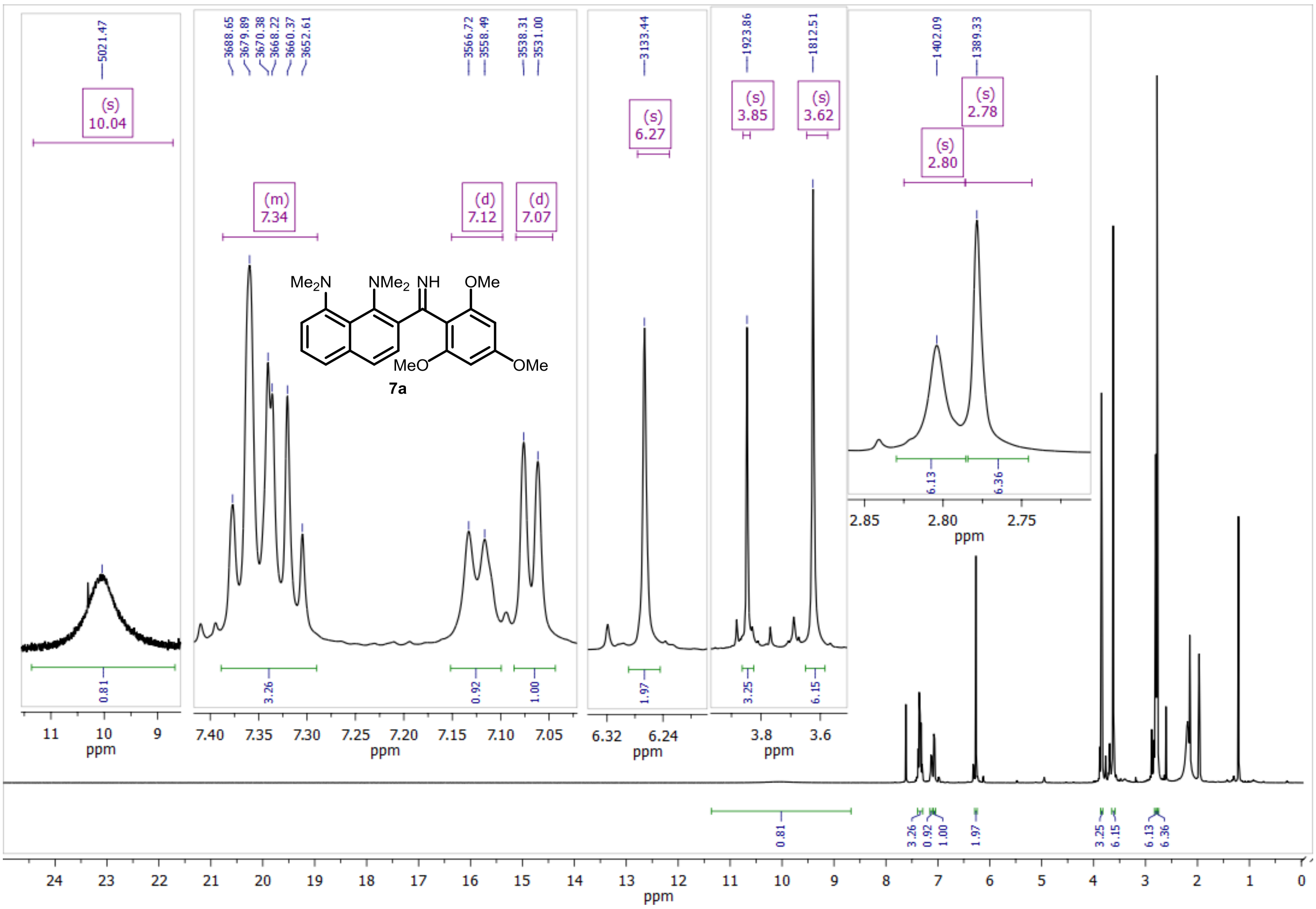

Figure S28. ^{1}H NMR spectrum of **7a**, CD_3CN, 500 MHz, 25 °C.

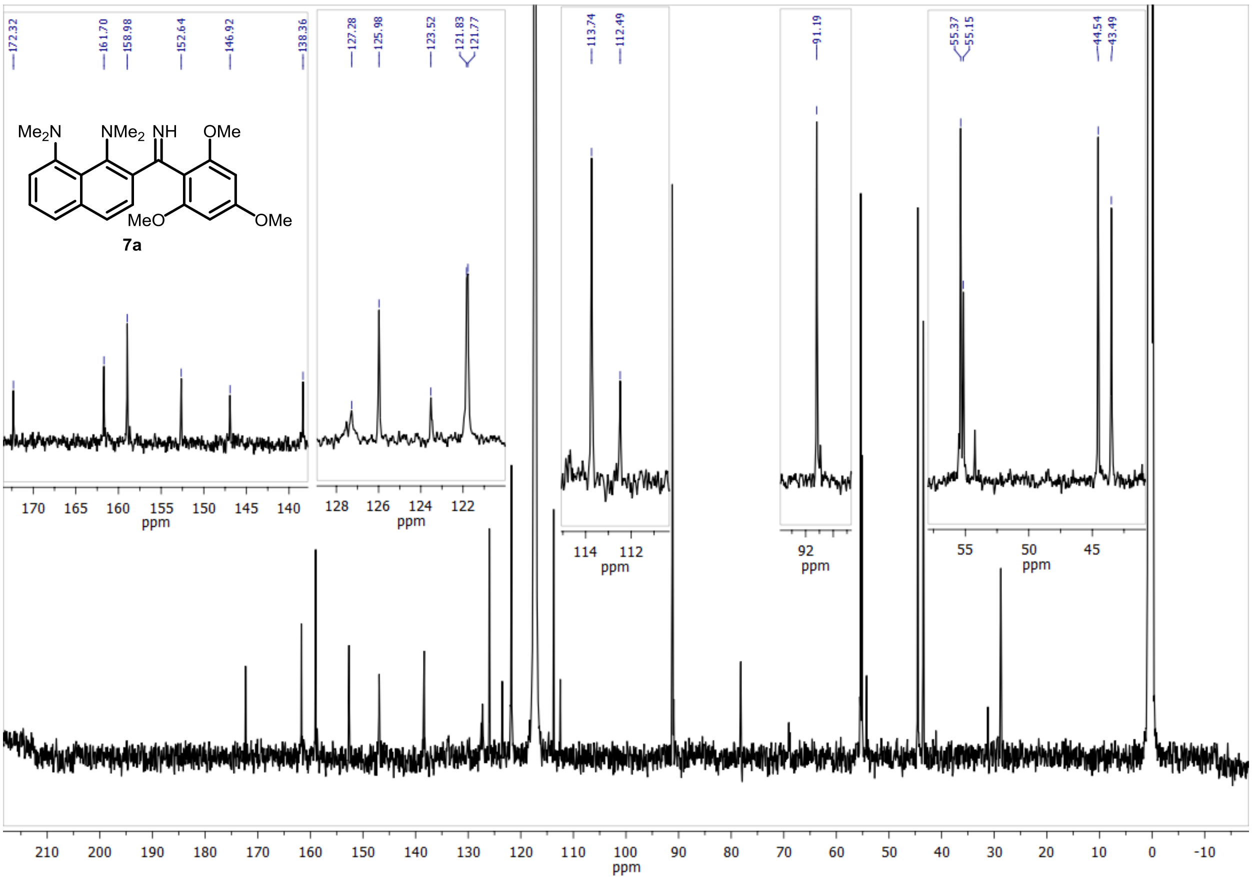

Figure S29. ^{13}C NMR spectrum of **7a**, CD_3CN, 126 MHz, 25 °C.

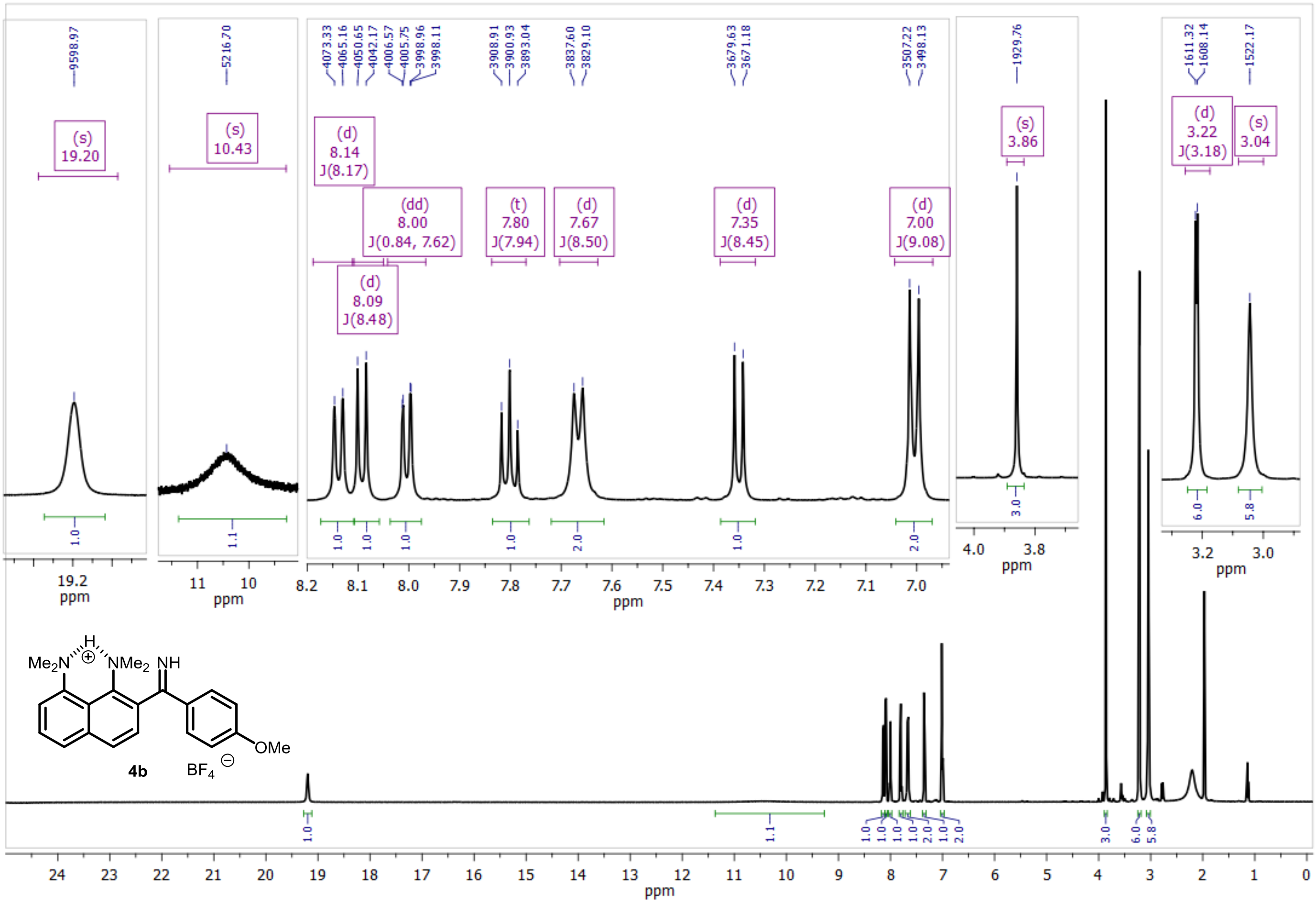

Figure S30. ^{1}H NMR spectrum of **4b**, CD_3CN, 500 MHz, 25 °C.

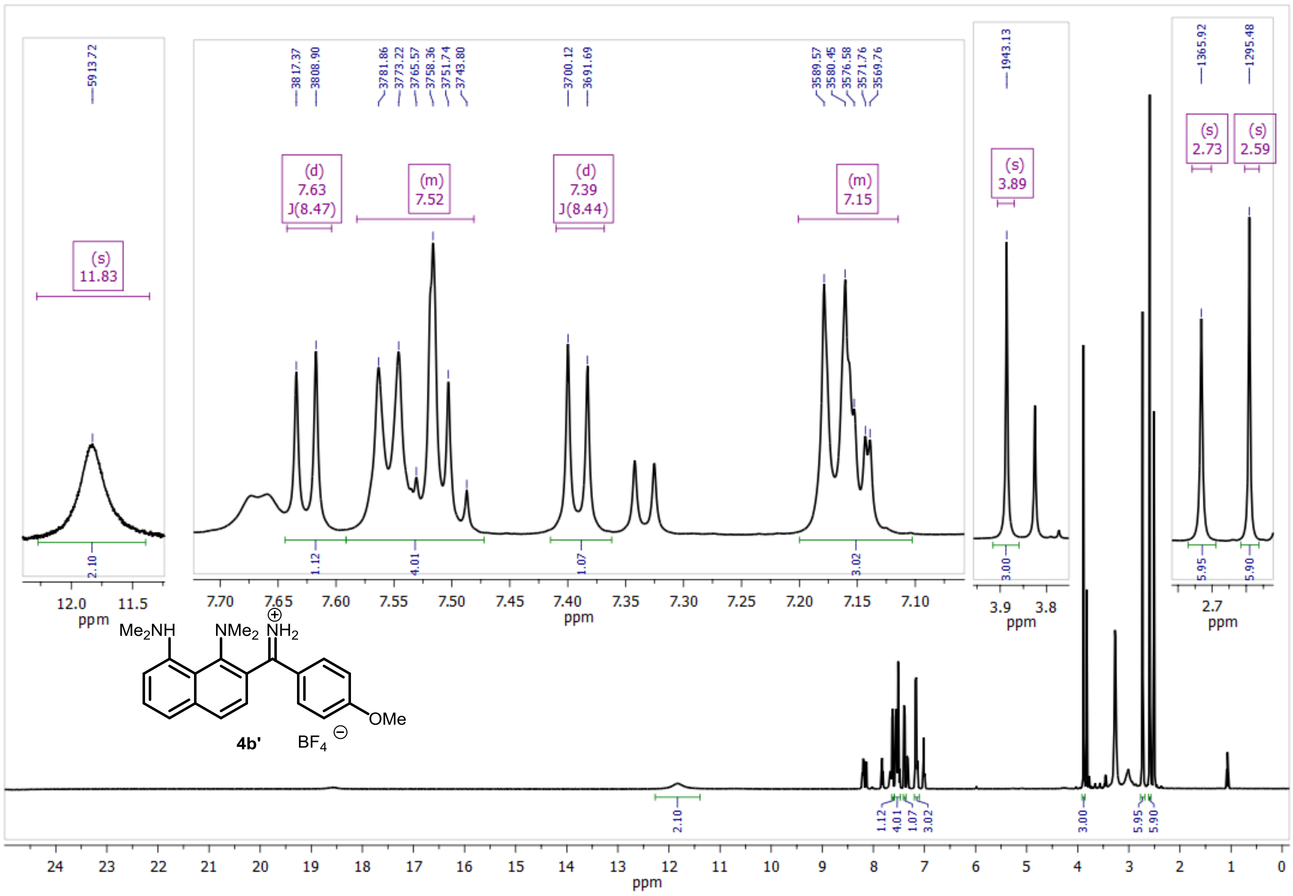

Figure S31. ^{1}H NMR spectrum of **4b⇌4b' mixture**, DMSO-d_6, 500 MHz, 25 °C (only signals of **4b'** are integrated).

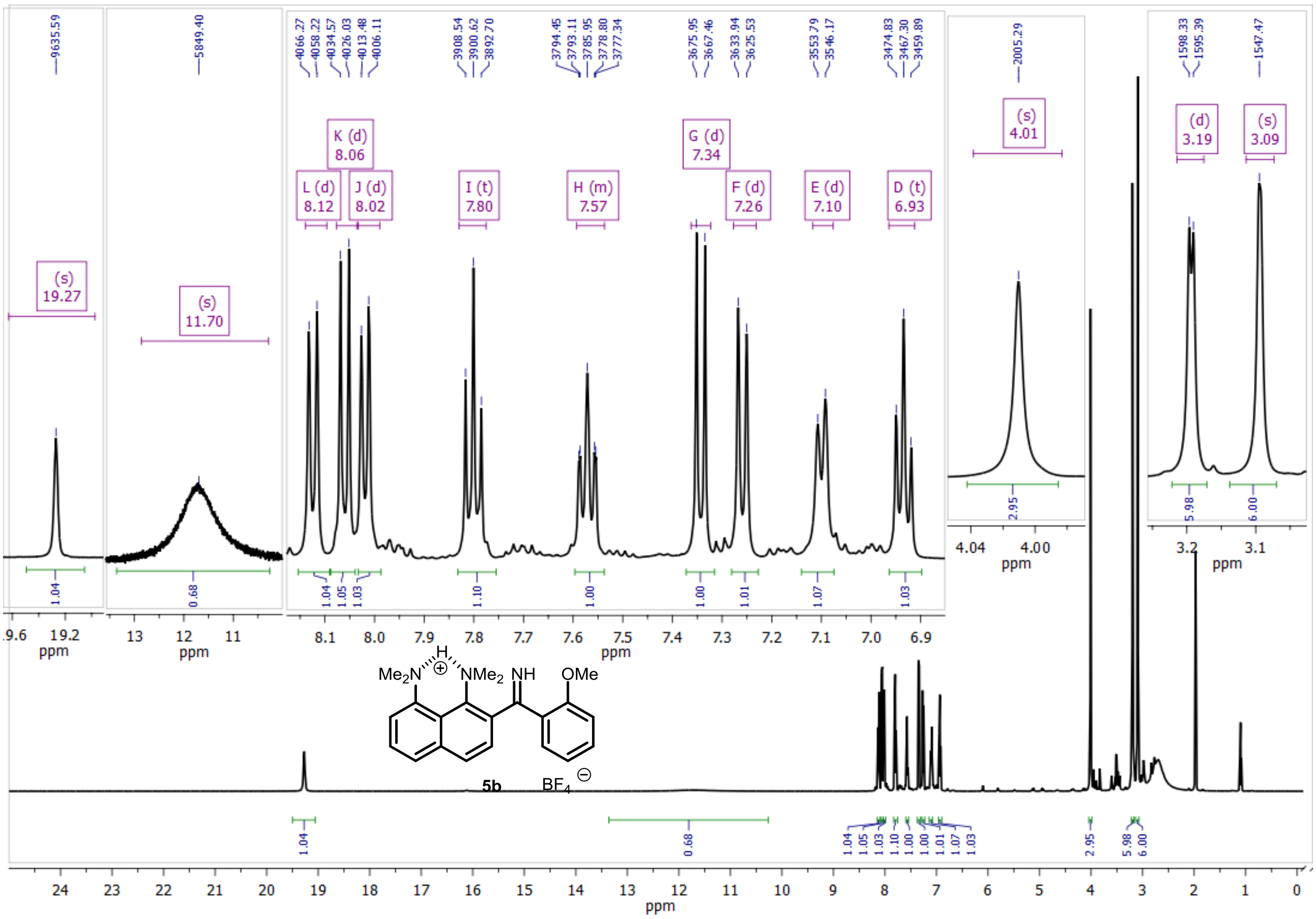

Figure S30. ^{1}H NMR spectrum of **5b**, CD_3CN, 500 MHz, −40 °C.

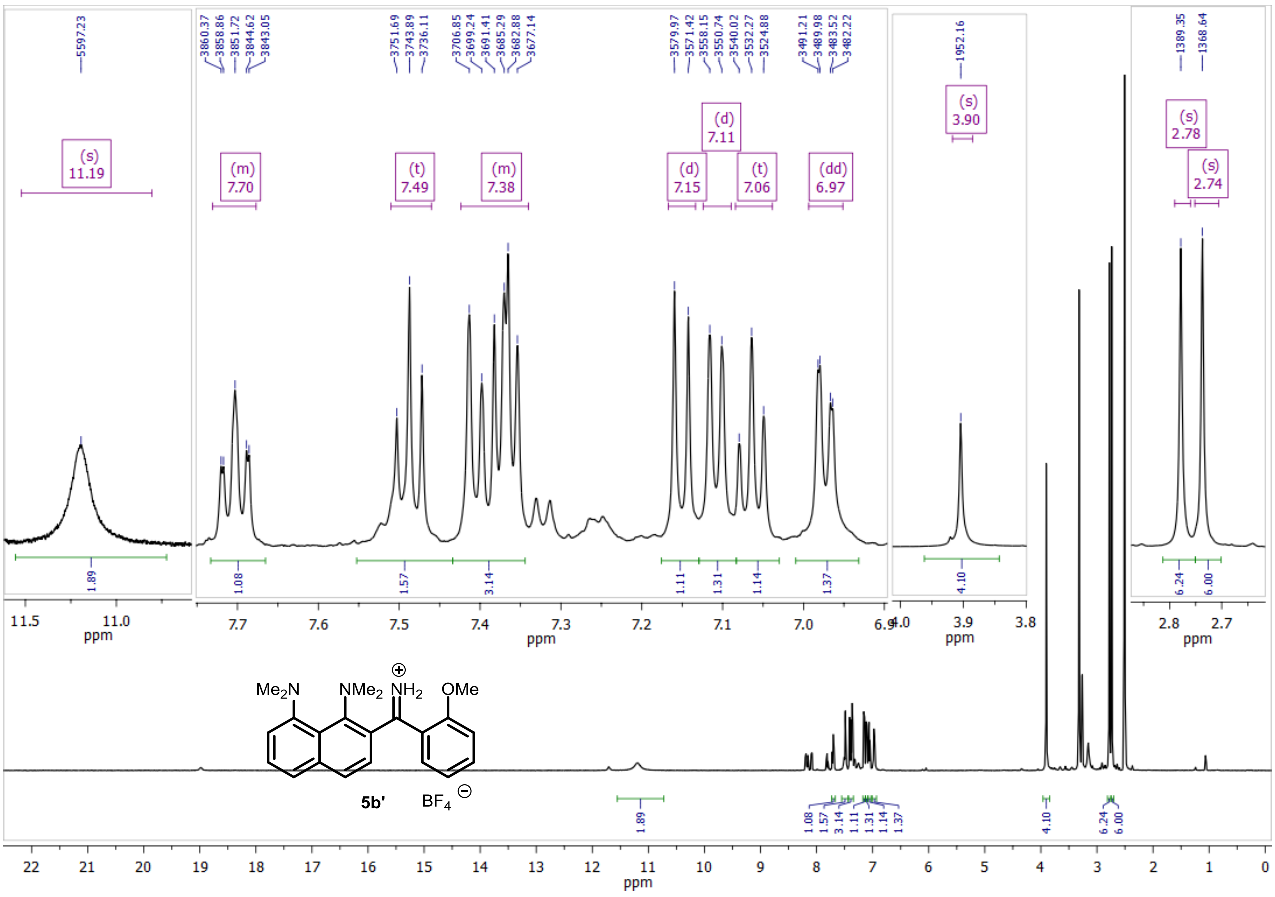

Figure S33. ^{1}H NMR spectrum of **5b⇌5b' mixture**, DMSO-d_6, 500 MHz, 25 °C (only signals of **5b'** are integrated).

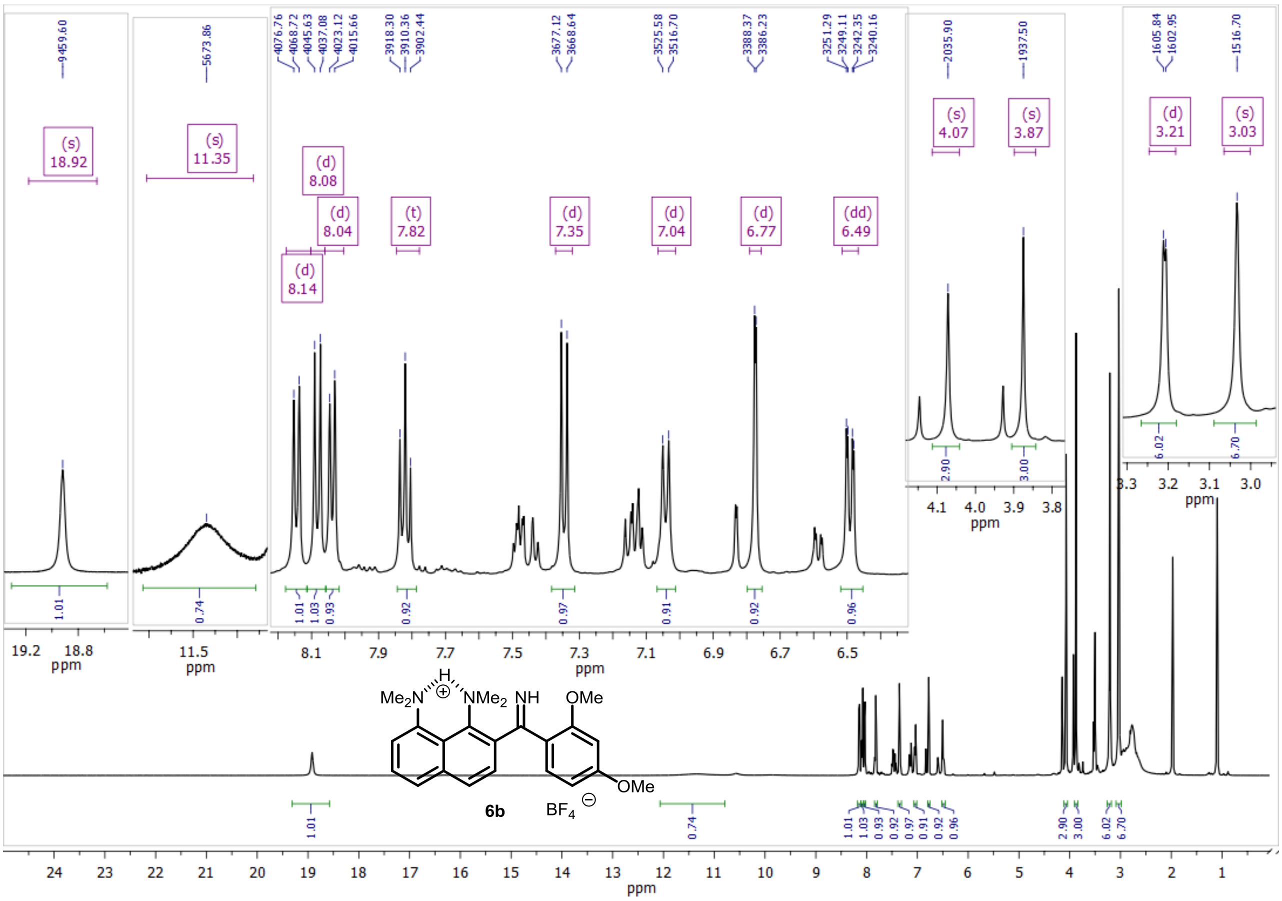

Figure S34. ^{1}H NMR spectrum of **6b⇌6b' mixture**, CD_3CN, 500 MHz, −40 °C (only signals of **6b** are integrated).

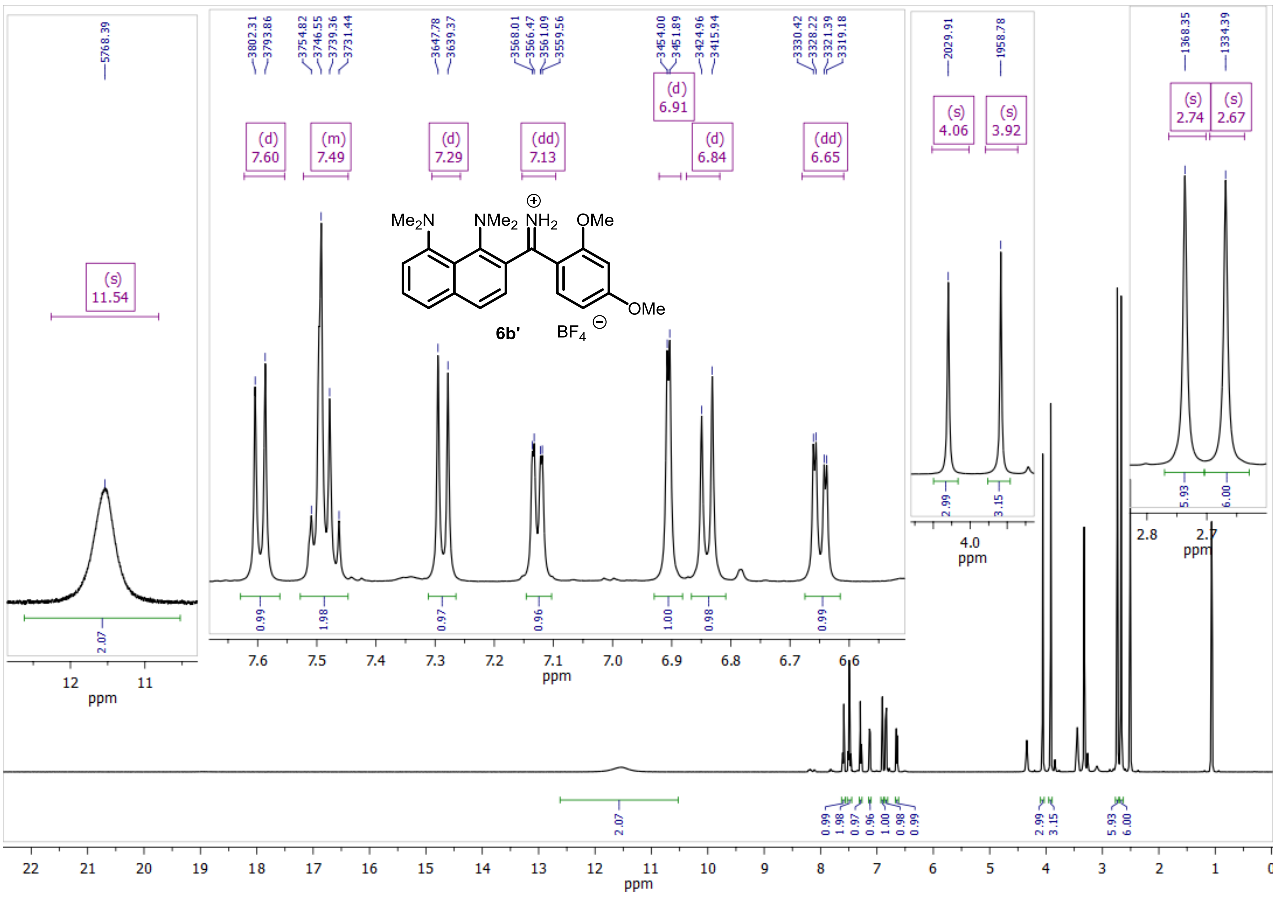

Figure S35. ^{1}H NMR spectrum of **6b⇌6b' mixture**, DMSO-d_6, 500 MHz, 25 °C (only signals of **6b'** are integrated).

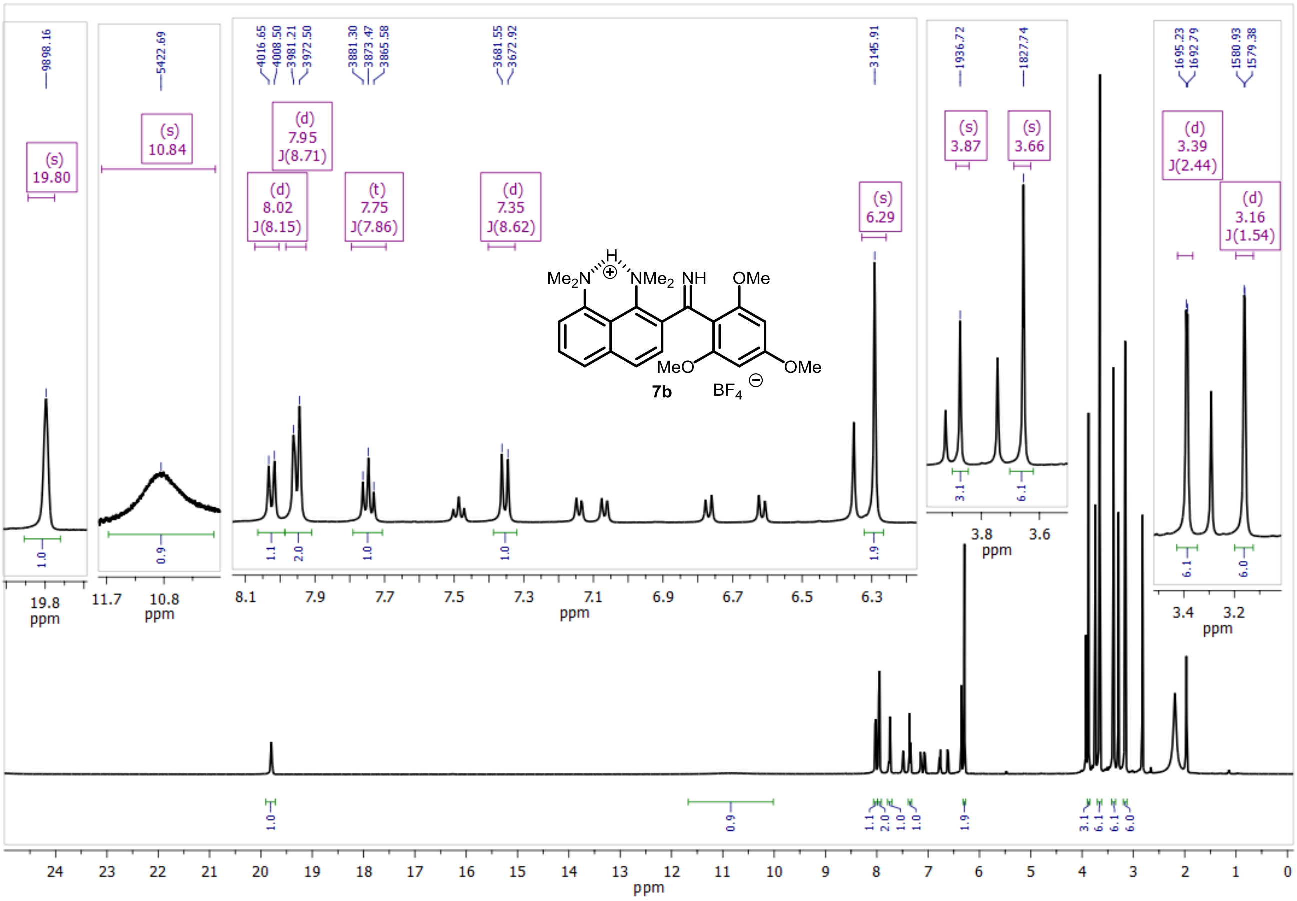

Figure S36. ^{1}H NMR spectrum of **7b⇌7b' mixture**, CD_3CN, 500 MHz, −40 °C (only signals of **7b** are integrated).

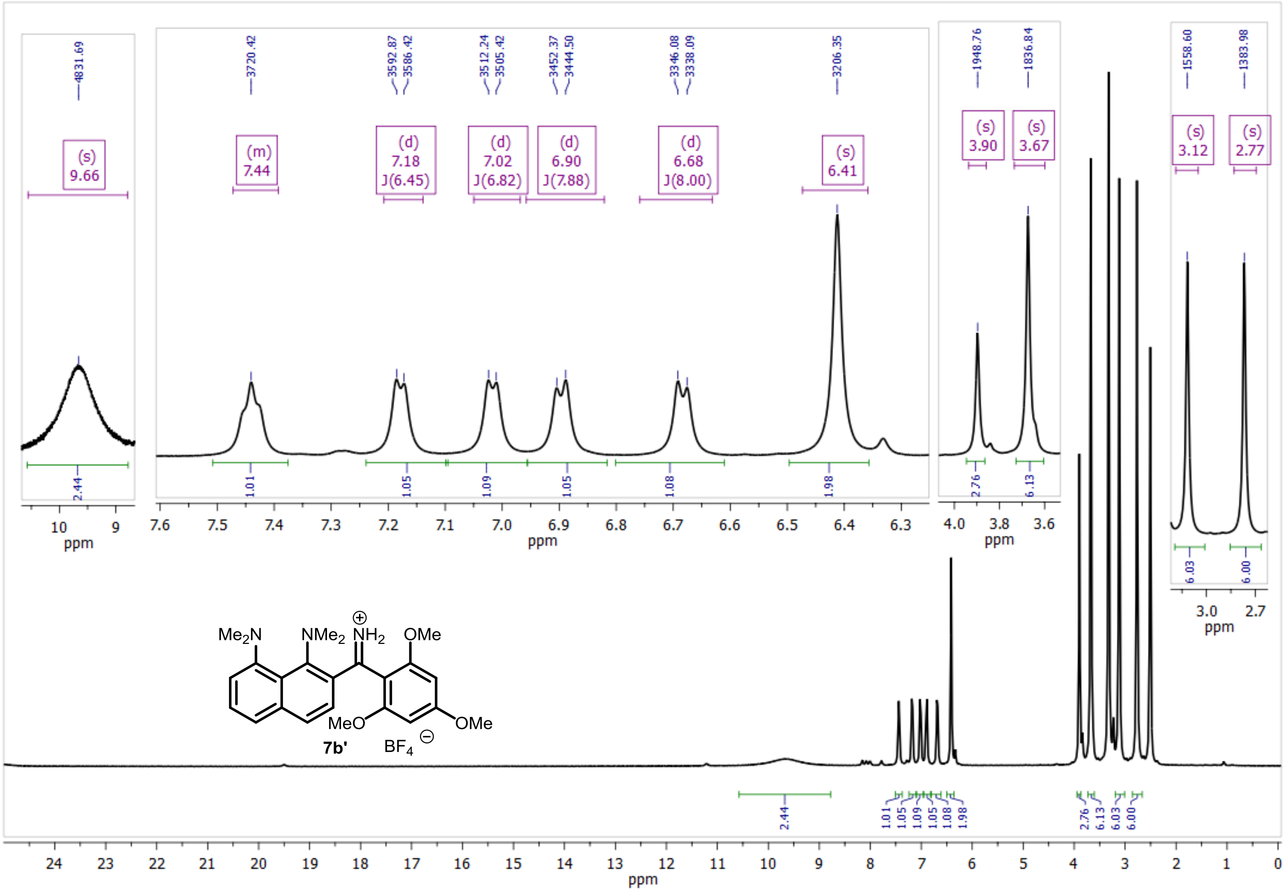

Figure S37. ^{1}H NMR spectrum of **7b**$\rightleftharpoons$**7b' mixture**, DMSO-d_6, 500 MHz, 25 °C (only signals of **7b'** are integrated).

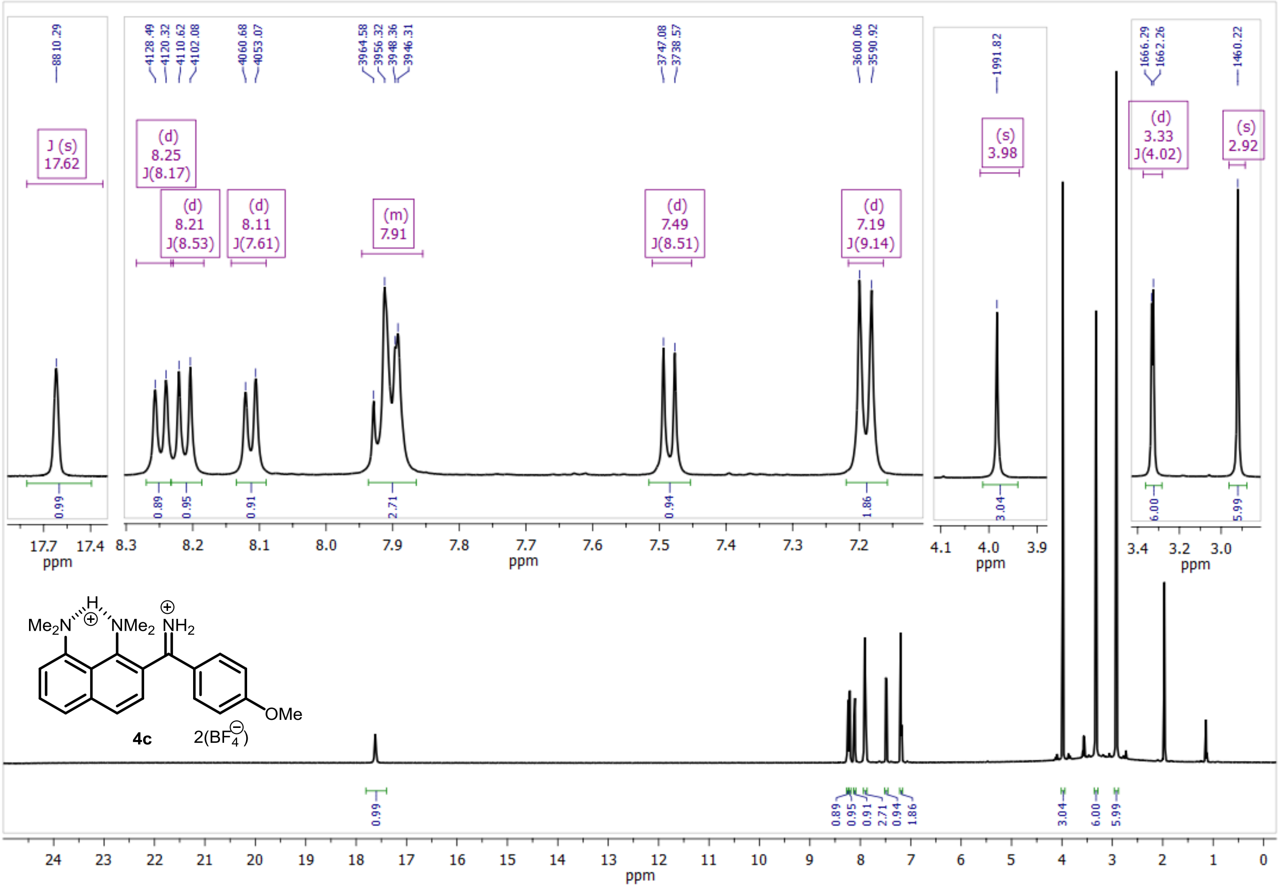

Figure S38. ^{1}H NMR spectrum of **4c**, CD_3CN, 500 MHz, 25 °C.

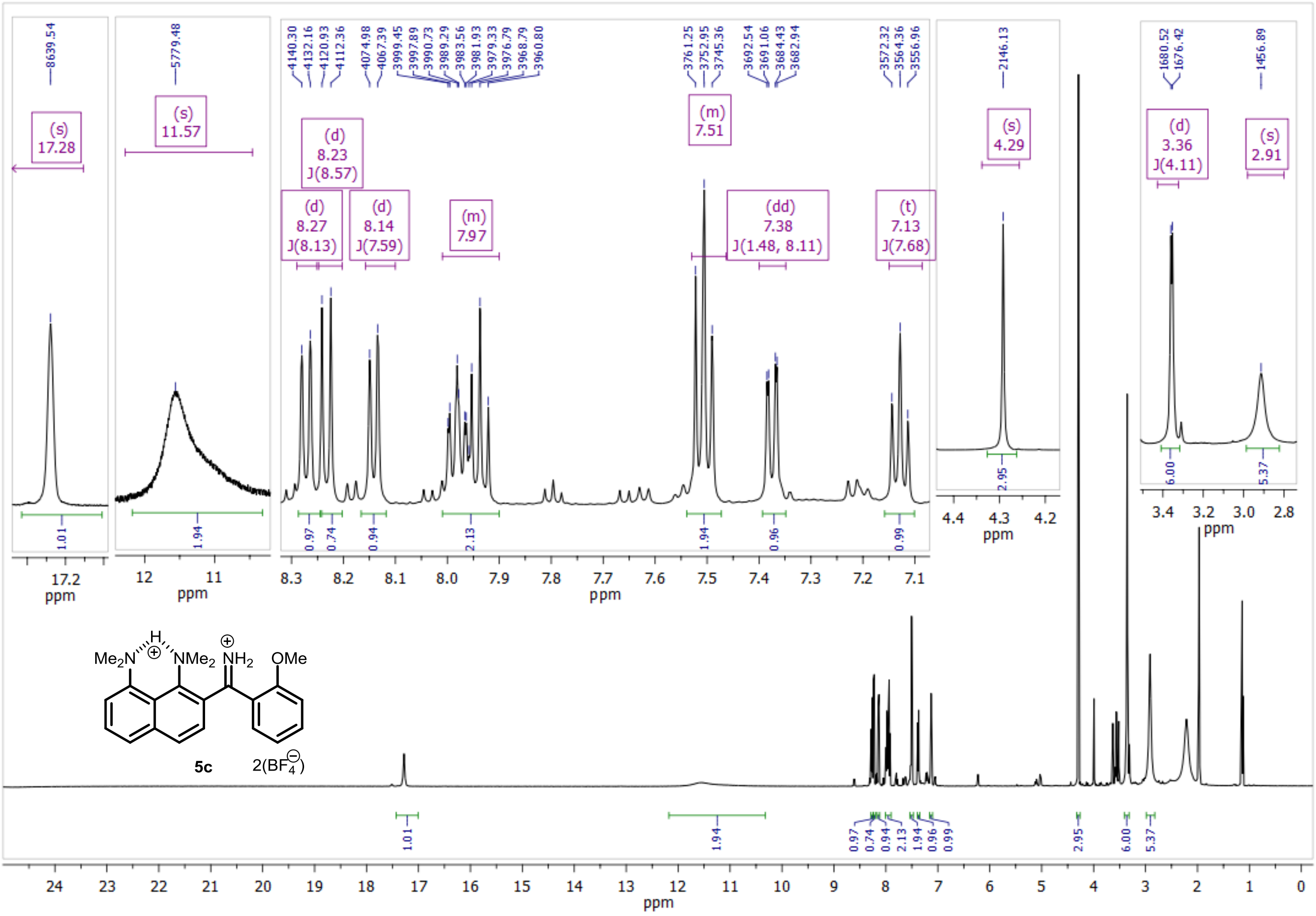

Figure S39. ^{1}H NMR spectrum of **5c**, CD_3CN, 500 MHz, 25 °C.

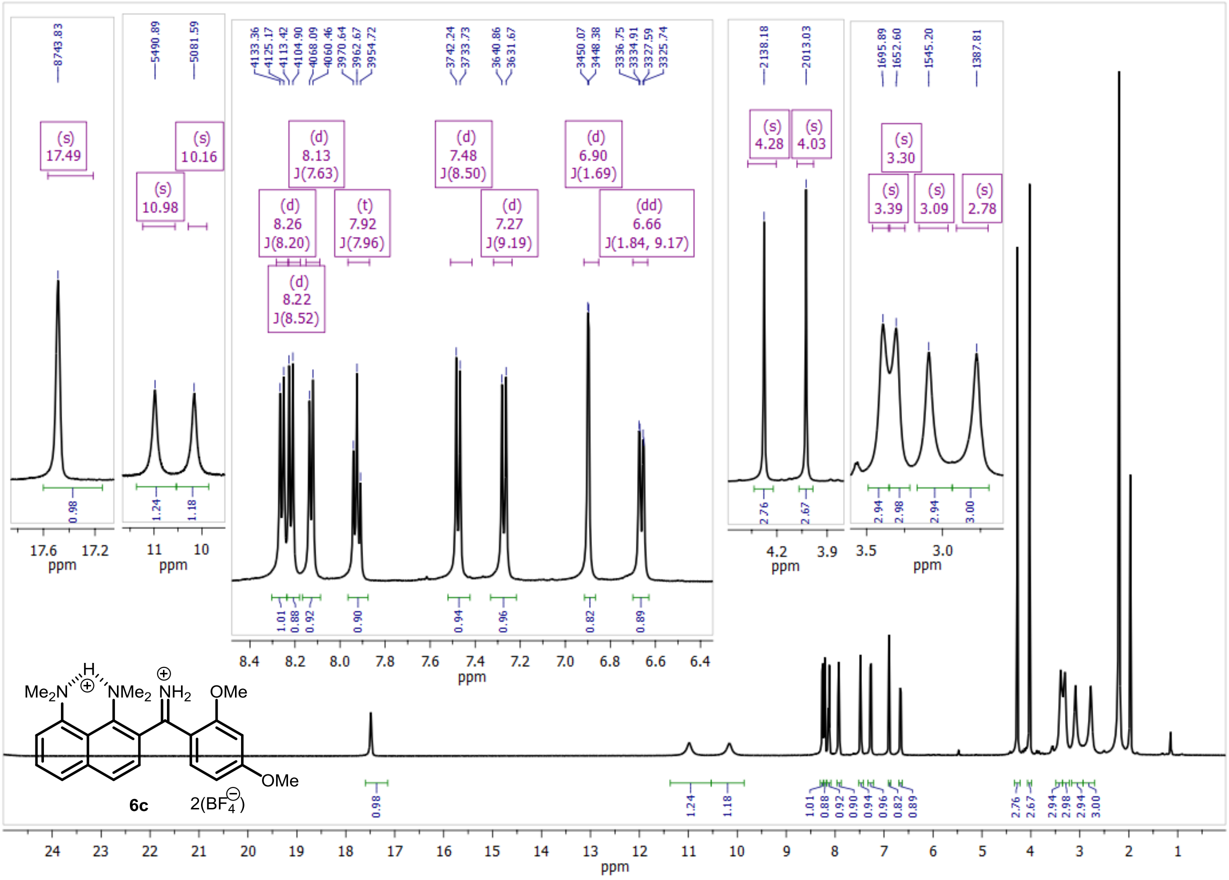

Figure S40. ^{1}H NMR spectrum of **6c**, CD_3CN, 500 MHz, 25 °C.

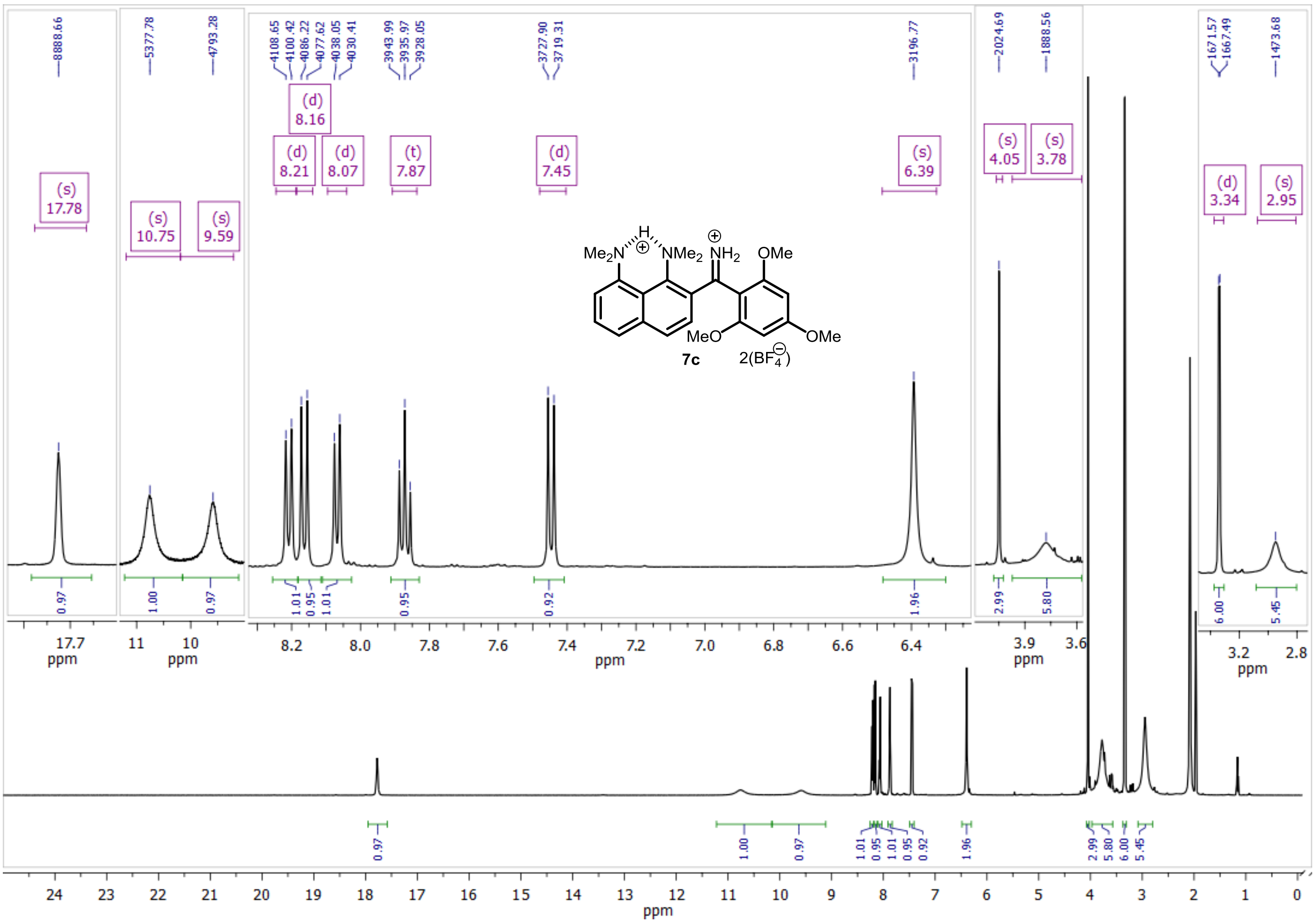

Figure S41. ^{1}H NMR spectrum of **7c**, CD_3CN, 500 MHz, 60 °C.

Table SI-1. Crystal data and structure refinement

**Parameter**	**6a**	**6b**	**6c**	**4a·$HClO_4$**
Empirical formula	$C_{23}H_{27}N_3O_2$	$C_{25}H_{34}BF_4N_3O_3$	$C_{23}H_{29}B_2F_8N_3O_2$	$C_{22}H_{26}ClN_3O_5$
Formula weight	377.47	511.36	553.11	447.91
Temperature/K	100(2)	100(2)	100(2)	120(2)
Crystal system	monoclinic	monoclinic	triclinic	monoclinic
Space group	$P2_1/n$	$P2_1/n$	P-1	$P2_1/c$
a/Å	13.5506(7)	7.61226(12)	7.6816(3)	7.7872(7)
b/Å	9.7144(3)	21.1202(4)	12.3759(5)	14.0217(12)
c/Å	15.9727(10)	15.7463(2)	15.8693(5)	20.1562(18)
α/°	90	90	112.684(3)	90.00
β/°	111.673(6)	91.5601(14)	93.269(3)	94.971(2)
γ/°	90	90	93.060(3)	90.00
Volume/Å3	1953.94(19)	2530.63(7)	1385.01(9)	2192.6(3)
Z	4	4	2	4
ρcalcg/cm3	1.283	1.342	1.326	1.357
μ/mm-1	0.083	0.906	1.051	0.213
F(000)	808.0	1080.0	572.0	944.0
Crystal size/mm3	0.2 × 0.2 × 0.2	0.22 × 0.21 × 0.15	0.29 × 0.24 × 0.2	0.28 × 0.18 × 0.12
Radiation	MoKα (λ = 0.71073)	CuKα (λ = 1.54184)	CuKα (λ = 1.54184)	MoKα (λ = 0.71073)
2Θ range for data collection/°	5.296 to 54.996	7.004 to 143.726	6.058 to 143.216	4.06 to 58.68
Index ranges	-17 ≤ h ≤ 17<br>-12 ≤ k ≤ 11<br>-20 ≤ l ≤ 17	-9 ≤ h ≤ 9<br>-25 ≤ k ≤ 25<br>-19 ≤ l ≤ 19	-9 ≤ h ≤ 9<br>-15 ≤ k ≤ 15<br>-19 ≤ l ≤ 19	-10 ≤ h ≤ 10<br>-19 ≤ k ≤ 19<br>-27 ≤ l ≤ 27
Reflections collected	11449	28374	27137	26618
Independent reflections	4497<br>R_{int} = 0.0250<br>R_{sigma} = 0.0306	4958<br>R_{in}t = 0.0343,<br>R_{sigma} = 0.0188	5370<br>R_{int} = 0.0364<br>R_{sigma} = 0.0193	5961<br>R_{int} = 0.0602<br>R_{sigma} = 0.0498
Data/restraints/parameters	4497/0/262	4958/0/336	5370/0/349	5961/1/302
Goodness-of-fit on F2	1.038	1.027	1.084	1.039
Final R indexes [I>=2σ (I)]	R_1 = 0.0562<br>wR_2 = 0.1418	R_1 = 0.0424<br>wR_2 = 0.1107	R_1 = 0.0482<br>wR_2 = 0.1371	R_1 = 0.0560<br>wR_2 = 0.1266
Final R indexes [all data]	R_1 = 0.0681<br>wR_2 = 0.1500	R_1 = 0.0435<br>wR_2 = 0.1117	R_1 = 0.0533<br>wR_2 = 0.1415	R_1 = 0.0826<br>wR_2 = 0.1389
Largest diff. peak/hole/e Å-3	1.85/-0.23	0.88/-0.38	0.42/-0.33	0.55/-0.39
**CCDC**	**1867639**	**1867640**	**1867641**	**1867642**
